# Preliminary Review of Indian Eumenophorinae (Araneae: Theraphosidae) with Description of a New Genus and Five New Species from the Western Ghats

**DOI:** 10.1371/journal.pone.0087928

**Published:** 2014-02-14

**Authors:** Zeeshan A. Mirza, Rajesh V. Sanap, Harshal Bhosale

**Affiliations:** 1 Post-Graduate Program in Wildlife Biology & Conservation, Wildlife Conservation Society-India Program, National Centre for Biological Sciences, Tata Institute of Fundamental Research, Bangalore, India; 2 Post-Graduate Program in Ecology and Environment, Indian Institute of Ecology and Environment, New Delhi, India; 3 Postgraduate Program in Environmental Sciences, Department of Environmental Science, Fergusson College, Pune, Maharashtra, India; CSIR- National institute of oceanography, India

## Abstract

The theraphosid spider genera *Heterophrictus* Pocock, 1900 and *Neoheterophrictus* Siliwal & Raven, 2012 are rediagnosed in this paper and a new genus, *Sahydroaraneus* **gen. nov.** is described from Southern Western Ghats. Four new species (two each of *Heterophrictus* and *Neoheterophrictus*) and one of *Sahydroaraneus* **gen. nov.** are described from the Western Ghats. *Plesiophrictus mahabaleshwari* Tikader, 1977 is removed from the synonymy of *Heterophrictus milleti* Pocock, 1900 and is treated as a junior synonym of *Heterophrictus blatteri* (Gravely, 1935). *Plesiophrictus bhori* Gravely, 1915 is transferred to the genus *Neoheterophrictus*, *Neoheterophrictus bhori* (Gravely, 1915) **new combination**. The genus, *Sahydroaraneus* **gen. nov.**, resembles tarantula belonging to the genus, *Neoheterophrictus* but differs with respect to structure of tibial apophysis and spermathecae. Detailed ultra-structure of setae type of the Indian Eumenophorinae is presented for the first time along with notes on their biogeography. Common elements among Africa, Madagascar and India like the Eumenophorinae and several other mygalomorph spiders advocate mygalomorphae as an important group for evolutionary investigation due to their inability for long distance dispersal rendering the members restrictive in distribution.

## Introduction

Theraphosidae Thorell, 1870 is the most speciose of all mygalomorph spider families with 946 species and 124 genera [1], [2]. In India, it is represented by eleven genera and fifty five species [3]–[5]. Major taxonomic work concerning the family was carried out about 100 years ago [see 6–8] with the exception of the generic revision by Raven [9]. Theraphosidae in India is represented by the following subfamilies: Eumenophorinae, Ischnocolinae, Poecilotheriinae, Selenocosmiinae, Selenogyrinae and Thrigmopoeinae. There have been some notable additions to the list of Indian theraphosid spider by Tikader [10]. However, there are erroneous implications which have recently been rectified [3], [11]–[13]. A new species of *Plesiophrictus* Pocock, 1899 was described from the Western Ghats [14] and two new species of *Poecilotheria* Simon, 1885 were also described [15], [16]. Recently, a new genus and three new species from the Western Ghats were also discovered [17] further highlighting the taxonomic richness and the lack of our knowledge of this family in India as a whole. Most species in India were described almost a century ago and lack valuable morphological details warranting redescription of most species [11–14).

The monotypic genus *Heterophrictus* Pocock, 1900 was described with *H. milleti* as its type species. The new genus was distinguished from *Plesiophrictus* by possessing procurved fovea in the former and straight in the later Pocock [6]. *Heterophrictus* was diagnosed by the presence of long setae above the suture of the prolateral face of the first coxae, the shape of thoracic fovea and the posterior sternal sigilla remote from sternal margin [18]. Doubts were raised on Pocock’s [6] and Simon’s [18] distinction, based on slight differences in shape of the fovea and setae in *Heterophrictus*/*Plesiophrictus* to be related to size and to be variable even among adults by Gravely [7]. *Heterophrictus* and *Ischnocollela* Strand, 1907 were synonymized with *Plesiophrictus* [9] since they share all characters of generic significance. Recently, *Heterophrictus* was resurrected from the synonymy of *Plesiophrictus* and placed in the subfamily Eumenophorinae, [19] adding a new subfamily to India. More details of the genus *Plesiophrictus* were added by Sanap & Mirza [12] which enables us to compare it with closely related genus *Heterophrictus* as well as *Neoheterophrictus* Siliwal & Raven, 2012 in Siliwal et al. [5].

The Western Ghats is a biodiversity hotspot and is known for its rich and endemic fauna [20]. With a few exceptions, however, its invertebrate fauna remains poorly known [13], [21]–[23]. While documenting the mygalomorph spiders of the Western Ghats several theraphosid spiders were collected. Four of the collected species could not be attributed to any of the known sub-family or genera from India following Pocock [6], Gravely [7], [8] and Smith & Kirk [24]. The collected material possessed type III stridulatory organ presented in Eumenophorinae [25] and Theraphosinae Thorell, 1869 as described by Vol [26]. The specimens differed from Theraphosinae because they lacked abdominal urticating setae and palp bulb keels [27] and were thus tentatively assigned to the sub-family Eumenophorinae. After comparison of the collected material with Raven [9], Guadanucci [5], [19] it was confirmed that the material belongs to the subfamily Eumenophorinae.

The Eumenophorinae, characterized by presence of stridulatory spike setae between coxae of leg I–IV and stiffened brush of setae on palp femur in most genera, are presently known by eleven genera [28] distributed throughout Africa, southwestern countries in Saudi Arabia, Madagascar and associated islands [25], [29]–[31]. In the recent past, a new genus and a new species of the sub-family Eumenophorinae was described from Mauritius [29] was described which lacks stridulatory setae. Even though valuable contributions were made through publications of Smith [30], [31] despite this, members of the sub-family remain poorly known.

After a detailed comparison of the specimens collected from the Western Ghats with Guadanucci [19], Raven [9] and Smith [30], [31], it was found that the specimens belonged to two different genera and four new species. Based on Guadanucci [19] and Siliwal et al. [5], two of the species were attributed to the genus *Heterophrictus* and the other two species were accommodated into the newly described genus *Neoheterophrictus* Siliwal & Raven, 2012. *Neoheterophrictus* was described with description of three new species from the Western Ghats of Karnataka [*17*] and it was mentioned that the new genus resembles *Plesiophrictus* and *Heterophrictus. Neoheterophrictus* differs from *Plesiophrictus* in many aspects, however its distinction from *Heterophrictus* has not been satisfactorily justified [12) and hence a rediagnosis of the genus is needed. Recent collection of specimens of *Heterophrictus* and *Neoheterophrictus* of both sexes shed light on taxonomy of these genera based on which we rediagnose both the genera in this paper adding two new species to each from the Western Ghats.

**Figure 1:**
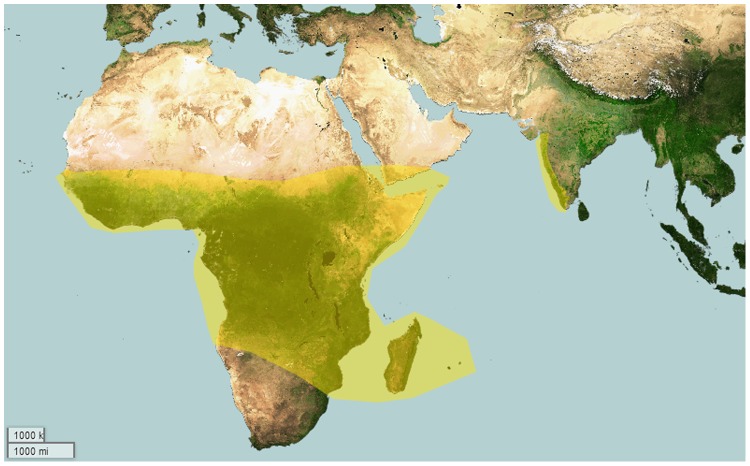
Possible Indian odysseys: different models for the position of India approximately 65 million years ago. a, The standard ‘biotic ferry’ model showing India isolated by large expanses of water. b, A limited ‘biotic (land) bridge’ model incorporating a narrow connection (Greater Somalia) with Africa. c, Another biotic bridge model assuming a different longitudinal position for India and showing connections with Madagascar, Africa and Asia (Hedges [42]).

Around 158–160 million years ago, Gondwana split and the Indo-Madagascan plate started separating from Africa. The Indian plate then broke away from Madagascar ca. 84–96 million years ago [32], drifted northward and collided with Eurasia between 55.5 and 66 million years ago. Thus, initially the Indo-Madagascan plate was isolated from Africa, and then the Indian plate was isolated from both Madagascar and Africa for extended periods of time. When India broke off from Africa and later from Madagascar, it carried away Gondwanan forms with it (Fig. 1a, b, c), most of which became extinct due to the late Cretaceous climatic conditions while others survived dispersing and colonizing suitable regions of India (Figure 1) [33]. Interestingly, majority of these Gondwanan forms are presently distributed in the Western Ghats and Sri Lanka, however more detailed surveys are necessary to confirm this. One such Gondwanan form is the frog genus *Nasikabatrachus*, which is a relic from the ancient Indo-Madagascan plate [34], [35]. Recently, the barychelid genus *Tigidia* Simon, 1892 was reported from the Western Ghats of India with description of three new species [36] which was until know known only from African region, further highlighting ancient biogeographic links.

As part of our study on Indian mygalomorphae, ZM & RS visited the Natural History Museum, London and examined types of material collected from India. Most material assigned to the genus *Plesiophrictus* was examined and were found to belong to the subfamily Eumenophorinae and have here been formally transferred to respective genera (Table 1). Hirst [37] described *Annandaliella travancorica* based on a female specimen and later Gravely [7] assigned a male collected from near Cochin to *A. travancorica.* We examined the male and female specimens in the collection of the Natural History Museum and based on characters like absence of inter-cheliceral peg setae in the male and presence of horizontally aligned modified setae on the coxa of legs (a character unique to the Eumenophorinae) clearly justifies its placement in the Eumenophorinae. After detailed comparison with *Heterophrictus* and *Neoheterophrictus,* we here describe the male attributed to *A. travancorica* by Gravely [7] as a new species and erect a new genus to embody it.

**Table 1:** Taxonomic history of members of the genus *Plesiophrictus* and their current status.

Sr. no	Original name	Present status	Comment	Reference
**1**	*Plesiophrictus bhori* Gravely, 1915	*Neoheterophrictus bhori*	-	Present work
**2**	*Plesiophrictus blatteri* Gravely, 1915	*Heterophrictus blatteri*	Material from type locality examined	Present work
**3**	*Plesiophrictus collinus* Pocock,1899	*Sahydroaraneus collinus*	Type examined	Present work
**4**	*Plesiophrictus fabrei* Simon, 1892	*Plesiophrictus fabrei*	Type not examined	Pocock [6]
**5**	*Plesiophrictus linteatus* Simom, 1891	*Plesiophrictus linteatus*	Type not examined	Pocock [6]
**6**	*Plesiophrictus madraspatanus* Gravely, 1935	*Neoheterophrictus madraspatanus*	–	Sanap & Mirza [12]
**7**	*Plesiophrictus mahabaleshwari* Gravely, 1977	*Heterophirctus blatteri*	New synonymy	Present work
**8**	*Plesiophrictus meghalyaensis* Tikader,1977	*Plesiophrictus meghalyaensis*	Likely not a *Plesiophrictus*	Tikader [10]
**9**	*Plesiophrictus millardi* Pocock,1899	*Plesiophrictus millardi*	Topotype examined	Sanap & Mirza [12]
**10**	*Plesiophrictus milleti* Pocock, 1900	*Heterophrictus milleti*	NHM material examined	Guadanucci [19]
**11**	*Plesiophrictus nilagiriensis* Siliwal, 2007	*Plesiophrictus nilagiriensis*	–	Siliwal et al. [14]
**12**	*Plesiophrictus raja* Gravely, 1915	*Sahydroaraneus raja*	Type examined	Present work
**13**	*Plesiophrictus sataraensis* Gravely, 1915	*Plesiophrictus satarensis*	Type examined	Gravely, [7]
**14**	*Plesiophrictus sericeus* Pocock, 1900	*Plesiophrictus sericeus*	Type examined	Pocock, [6]

## Methods

Specimens were collected during the surveys conducted in Aarey Milk Colony (Mumbai, Maharashtra) and on random visits to other collection areas. Specimens have been deposited at Zoological Survey of India/Western Regional Centre, Pune (ZSI/WRC) and Bombay Natural History Society, Mumbai, Maharashtra (BNHS). Comparative material for the study was examined from the collection of the Natural History Museum, London and diagrams of type specimens from University of Copenhagen, Zoological Museum (ZMUC, Denmark). Measurements of body parts were taken with an Aerospace Dial Caliper. Measurements of the chelicerae have being taken of the lateral aspect after dissection. All measurements are in mm. and total length excludes chelicerae length. Spermathecae were dissected and cleaned in clove oil using needles. Specimens were examined using Labomed CSM2 stereo-binocular microscope. Photographs of the specimens in India were taken with a Nikon D50 mounted with a Nikon 105 mm macro and extension tubes and those at the Natural History Museum (NHM, London) with an imaging system on Leica MZ12S. Descriptive style follows standardized descriptive style provided by Raven [38]. Specimens were coated with 50 mA of platinum for 50 seconds and were scanned using Joel JSM-7600F Field Emission Scanning Electron Microscope for analyzing ultra structure of stridulatory and tarsal setae. Abbreviations: AMC – Aarey Milk Colony, ALE – Anterior lateral Eyes; AME – Anterior Median Eyes; BMNH – Natural History Museum, London; BNHS – Bombay Natural History Society, Mumbai; d – dorsal; fe – femur; HB – Harshal Bhosale; mt – metatarsus; MOQ – Median ocular quadrate; p – Prolateral; pa – patella; PLE – Posterior lateral eyes; PLS – Posterior lateral spinnerets; PME – Posterior median eyes; PMS – Posterior median spinnerets; r – retrolateral; RS – Rajesh Sanap; ta – tarsus; ti – tibia; v – ventral; ZM – Zeeshan Mirza and ZSI/WRC – Zoological Survey of India/Western Regional Centre.

### Ethics Statement

All specimens were collected from areas outside Protected Areas and did not involve any endangered or protected species; hence collection and/or study permits were not required which complied with all relevant regulations. Permission to use data, diagrams and image of examined material was obtained from curators of museums for this study.

### Nomenclature Acts

The electronic edition of this article conforms to the requirements of the amended International Code of Zoological Nomenclature, and hence the new names contained herein are available under that Code from the electronic edition of this article. This published work and the nomenclatural acts it contains have been registered in ZooBank, the online registration system for the ICZN. The ZooBank LSIDs (Life Science Identifiers) can be resolved and the associated information viewed through any standard web browser by appending the LSID to the prefix “http://zoobank.org/”. The LSID for this publication is: urn:lsid:zoobank.org:pub: urn:lsid:zoobank.org:pub:856F44CD-CFA3-42CB-A728-5454157264B6. The electronic edition of this work was published in a journal with an ISSN, and has been archived and is available from the following digital repositories: PubMed Central, LOCKSS, Academia.edu and ResearchGate.

### Material Examined

*Annandaliella travancorica* Hirst, 1909 holotype female (BMNH 16.5.2.13) Travancore, Kerala, India.

*Batesiella crinita* Pocock, 1903 holotype female (BMNH 1903.6.30.23) Cameroona, Africa.

*Eumenophorus murphyi* Smith, 1990 female (BMNH 4859) Sierra Leone.

*Euphrictus spinosus* Hirst, 1909 holotype male *(*BMNH 1908.8.11.2), River Ja, Cemeroonsm Africa.

*Heterophrictus milleti* female, Nashik District, Maharashtra, India.

*Heterophrictus blatteri* female (BMNH 16.5.2.15) Satara district, Maharashtra, India.

*Hysterocrates crassipes* Pocock, 1897 female (BMNH 79.49).

*Monocentropus longimanus* Pocock, 1903 female (BMNH 1903.9.2.29).

*Plesiophrictus bhori* Gravely, 1915 female (BMNH 16.5.2.16) Parambikulam, Kerala, India.

*Plesiophrictus collinus* holotype female (BMNH 19.16.29) Yercaud in Shevaroy Hills, Tamil Nadu, India.

*Plesiophrictus millardi* male (BMNH 99.11 2.234) Matheran, Raighad District, Maharashtra, India; topotype male BNHS SP-62, Matheran, Raighad District, Maharashtra, India; BNHS SP-64 female, Aarey Milk Colony, Mumbai, Maharashtra, India.

*Plesiophrictus sataraensis* holotype male (BMNH 22.05.17), Medha, Yenna Valley, Satara District, Maharashtra, India.

*Plesiophrictus sericeus* Pocock, 1900 holotype female (BMNH 99.9.21.161) Eastern Pune, Pune District, Maharasshtra, India.

*Selenogyrus austinius* Smith, 1990 holotype female (BMNH 1896.11.15.5), Sierra Leone, Africa.

*Selenogyrus caeruleus* Pocock, 1897 holotype female (BMNH 1896.12.20.21) Sierra Leone, Africa.

## Results

### Taxonomic Treatment

#### Family Theraphosidae Thorell 1870

### Sub-family Eumenophorinae Pocock, 1897

Eumenophorinae Pocock 1897∶772; Raven 1985∶117; Smith 1995∶18.

Phoneyuseae Simon 1903∶918 & 948.

**Diagnosis:** Prolateral coxa I with a long paddle or spike-shaped seta aligned along the coxa and acting against numerous short transverse spiniform setae series on retrolateral surface of maxillae; also lyra similar to that (usually) on interface of coxae I and II or I–IV (Figure 13); palpal femora with a distinct brush of setae on retrolateral face (in most genera) and two distinct mounds at labio-sternal grove (Raven 1985).

**Figure 2:**
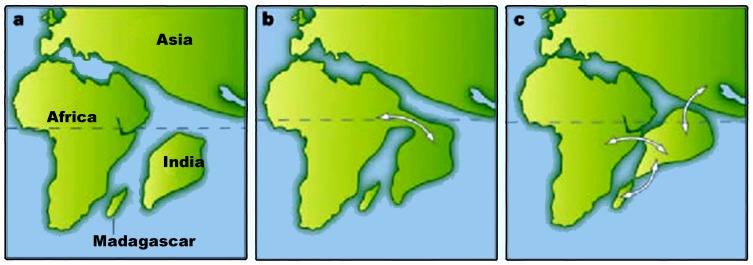
Map depicting global distribution of genera of Eumenophorinae.

**Figure 3:**
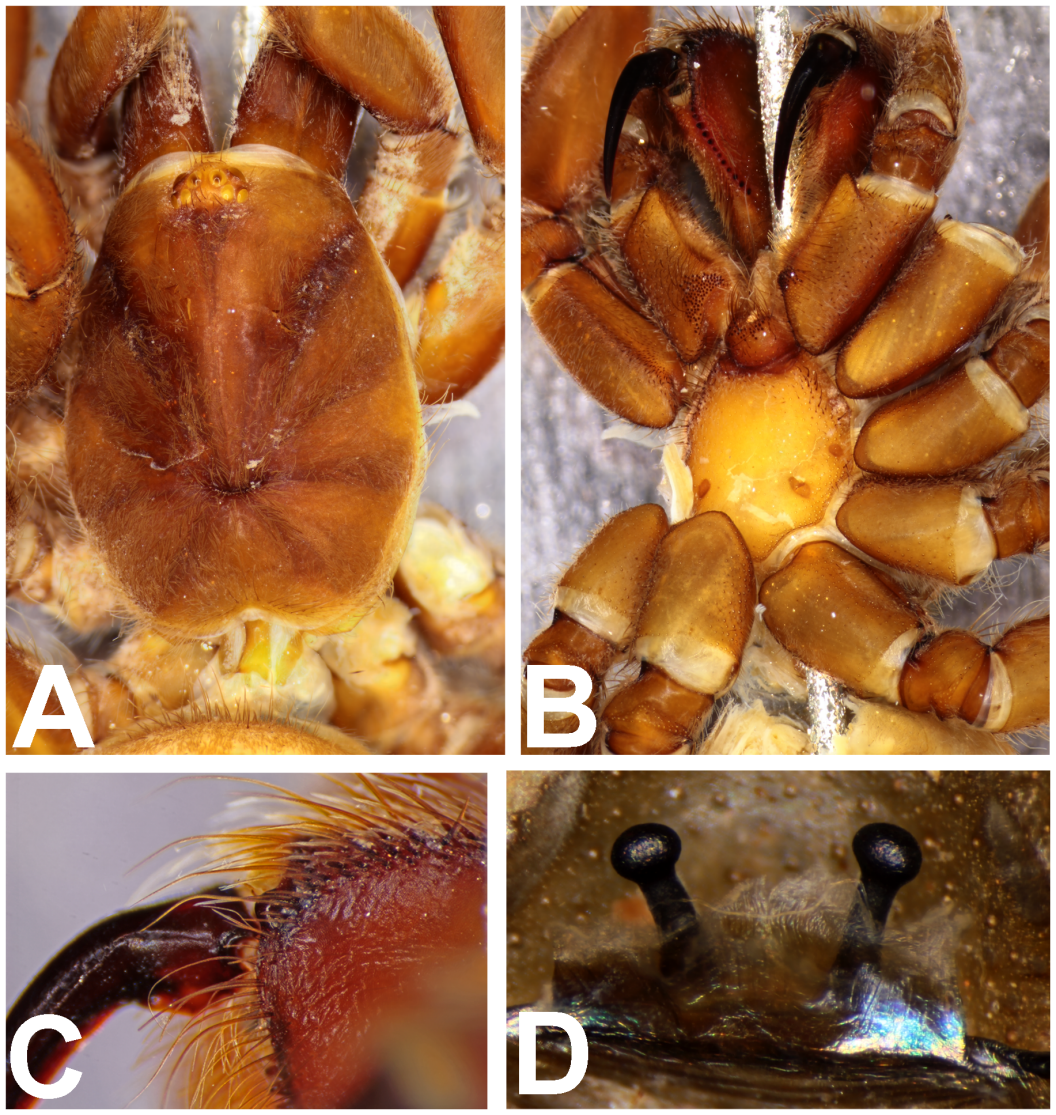
*Heterophrictus millet*. A. Cephalothorax; b. Sternum, labium and maxillae; C. Chelicerae prolateral view; D. spermathecae.

**Figure 4:**
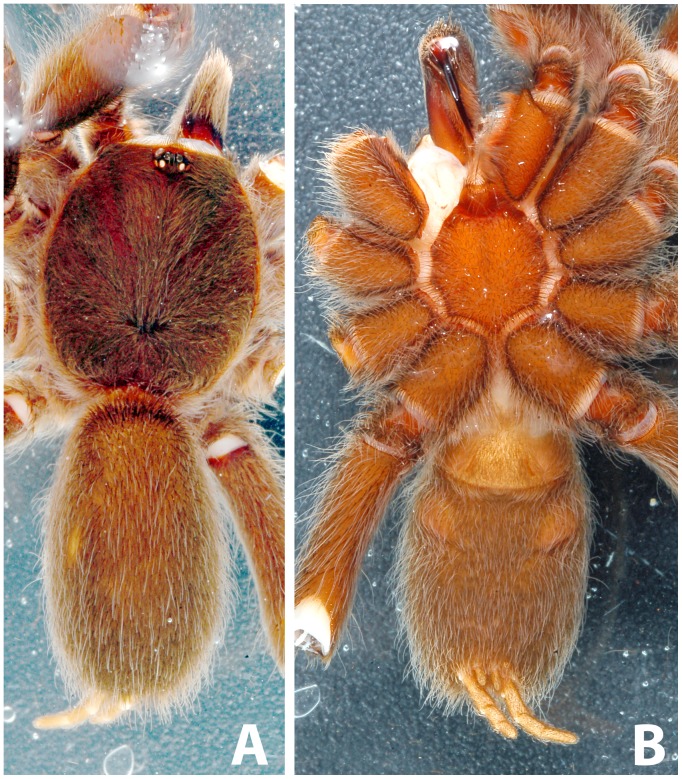
*Heterophrictus raveni* sp. nov. male holotype (ZSI/WRC/AR/418). A. Cephalothorax and abdomen dorsal view; B. Sternum, labium, maxillae and abdomen ventral view.

**Figure 5:**
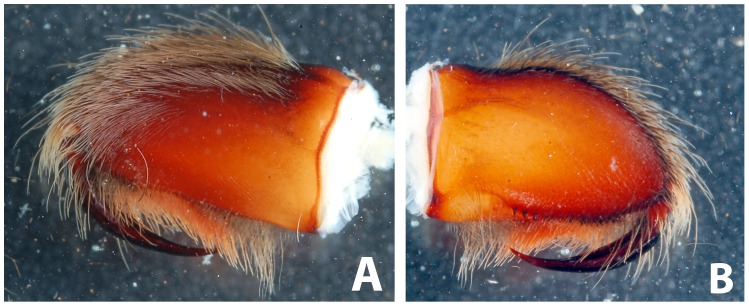
*Heterophrictus raveni* sp. nov. male holotype (ZSI/WRC/AR/418). A. Chelicerae retrolateral view; B. Chelicerae prolateral view.

**Figure 6:**
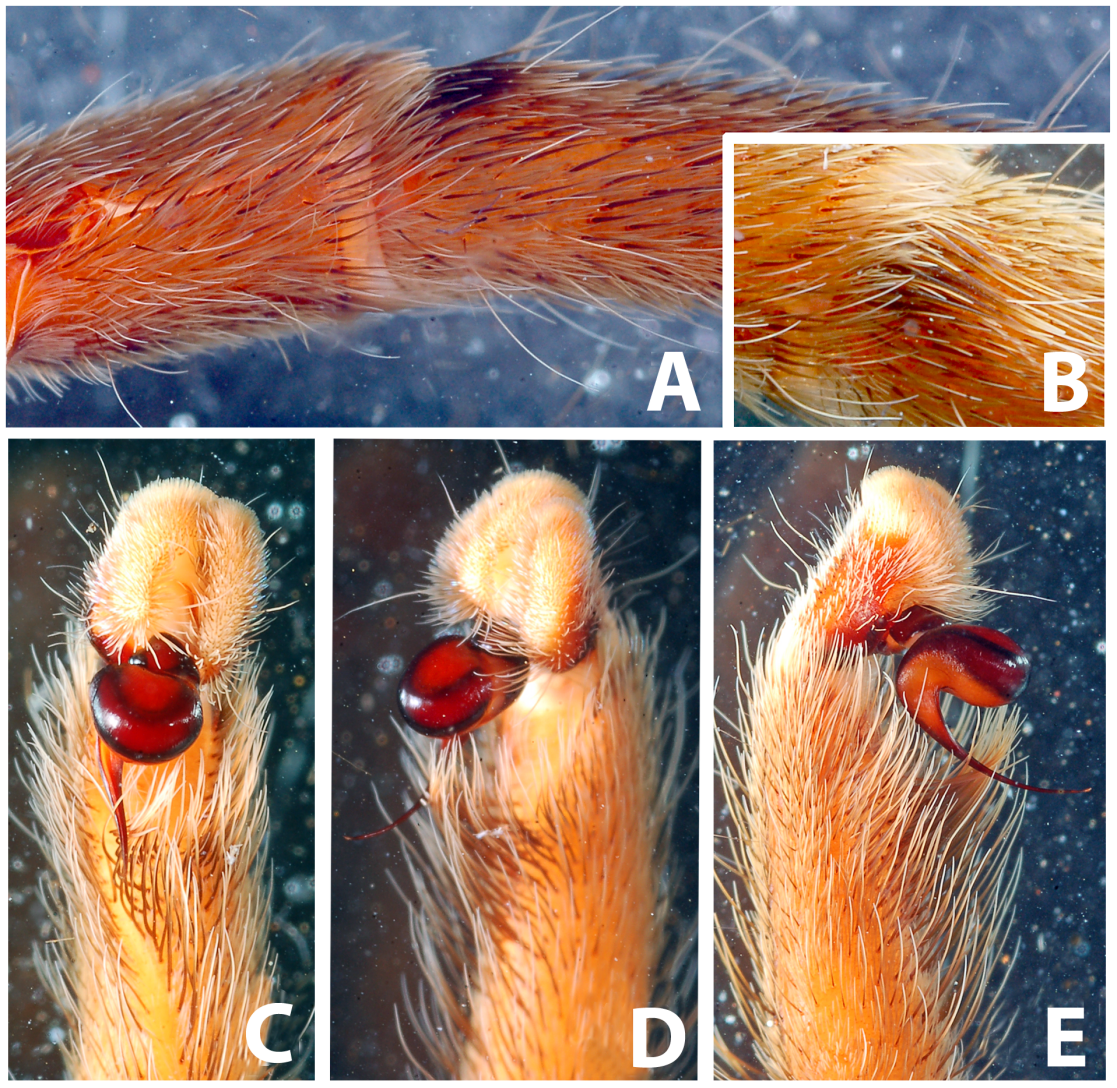
*Heterophrictus raveni* sp. nov. male holotype (ZSI/WRC/AR/418). A. Cluster of spiniform setae on basal region of tibia I, retrolateral view; B. Cluster of spiniform setae tibia I dorsal view; C. Palp bulb dorsal view; D. Palp bulb prolateral view; E. Palp bulb retrolateral view.

**Figure 7:**
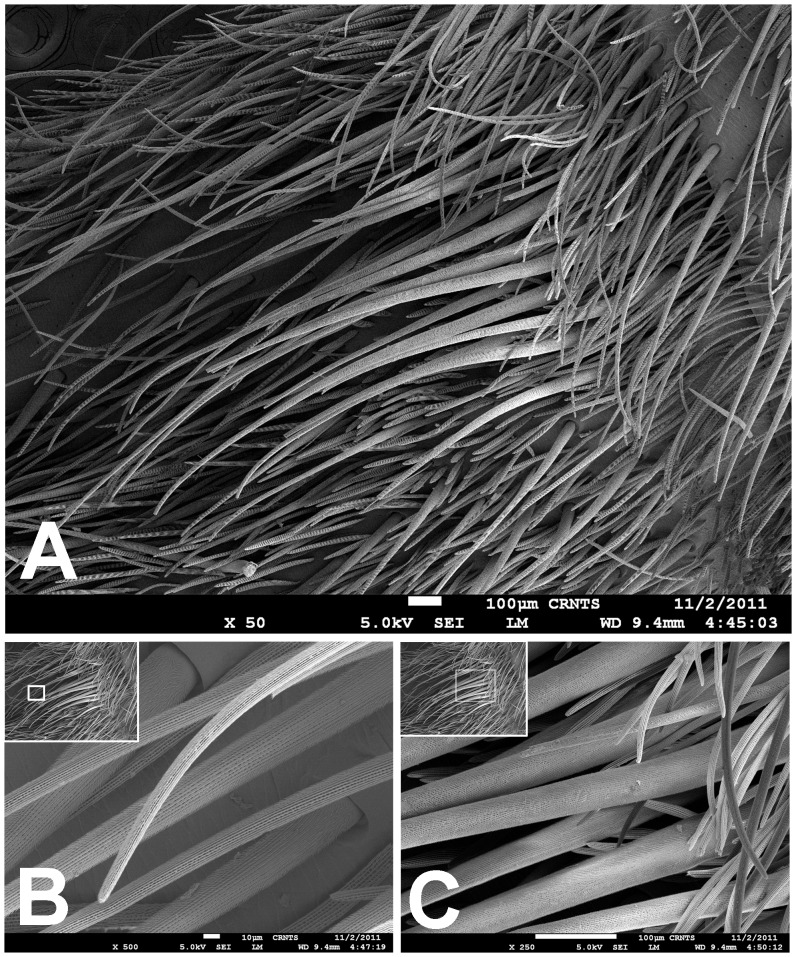
Scanning electron micrograph of *Heterophrictus raveni* sp. nov. male holotype tibia I (ZSI/WRC/AR/418). A. Ultra-structure of spiniform setae and normal setae, dorsal view; B. Tip of spiniform setae; C. Surface texture of spiniform setae.

**Figure 8:**
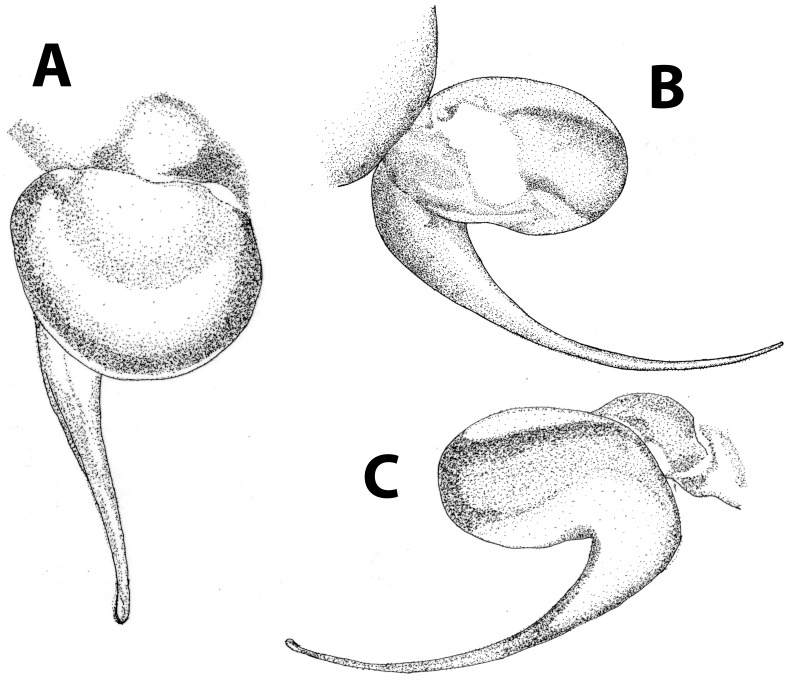
*Heterophrictus raveni* sp. nov. male holotype (ZSI/WRC/AR/418). A. Palp bulb dorsal view; B. Palp bulb prolateral view; C. Palp bulb retrolateral view.

**Figure 9:**
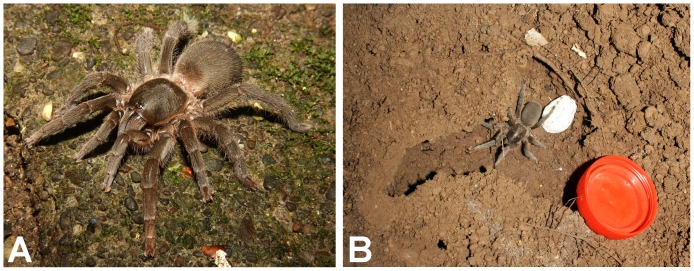
(A. & B.) *Heterophrictus raveni* **sp. nov.** female paratype (ZSI/WRC/AR/419) in life, photo by Zeeshan Mirza.

**Figure 10:**
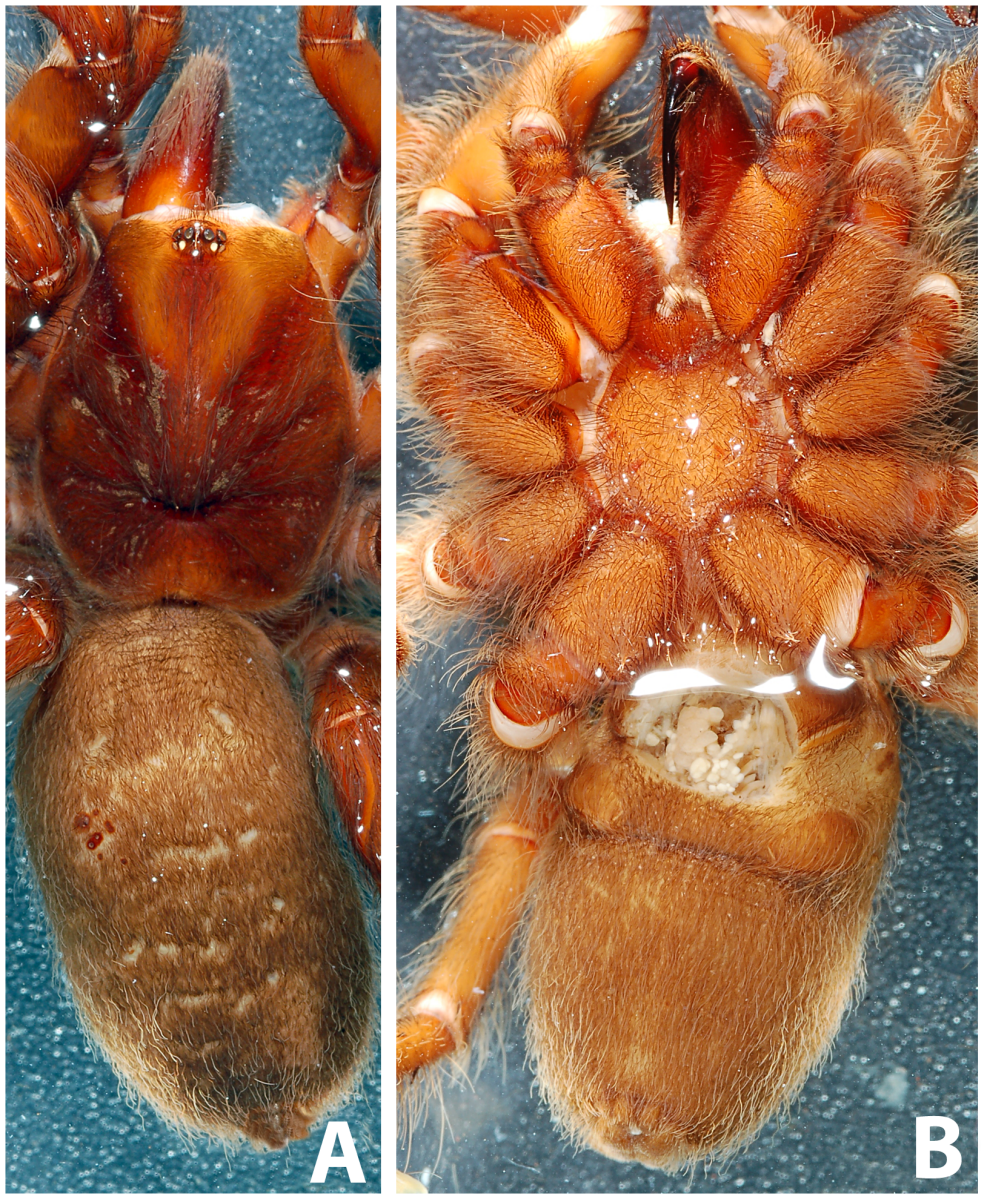
*Heterophrictus raveni* sp. nov. female paratype (ZSI/WRC/AR/419). A. Cephalothorax and abdomen, dorsal view; B. Sternum, labium, maxilla and abdomen, ventral view.

**Figure 11:**
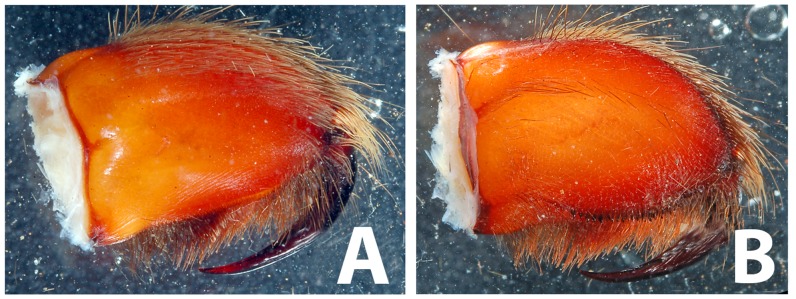
*Heterophrictus raveni* sp. nov. female paratype (ZSI/WRC/AR/419). A. Chelicerae retrolateral view; B. Chelicerae prolateral view.

**Figure 12:**
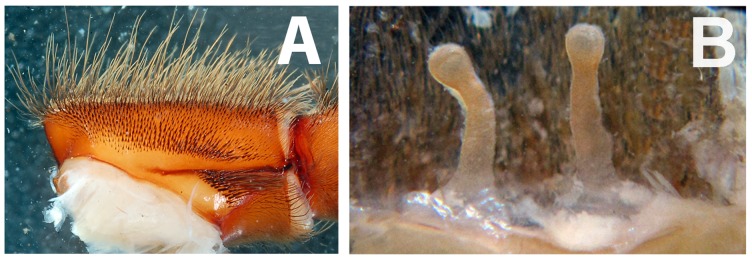
*Heterophrictus raveni* sp. nov. female paratype (ZSI/WRC/AR/419). A. Coxa of leg II prolateral view showing stridulatory setae; B. Spermathecae.

**Figure 13:**
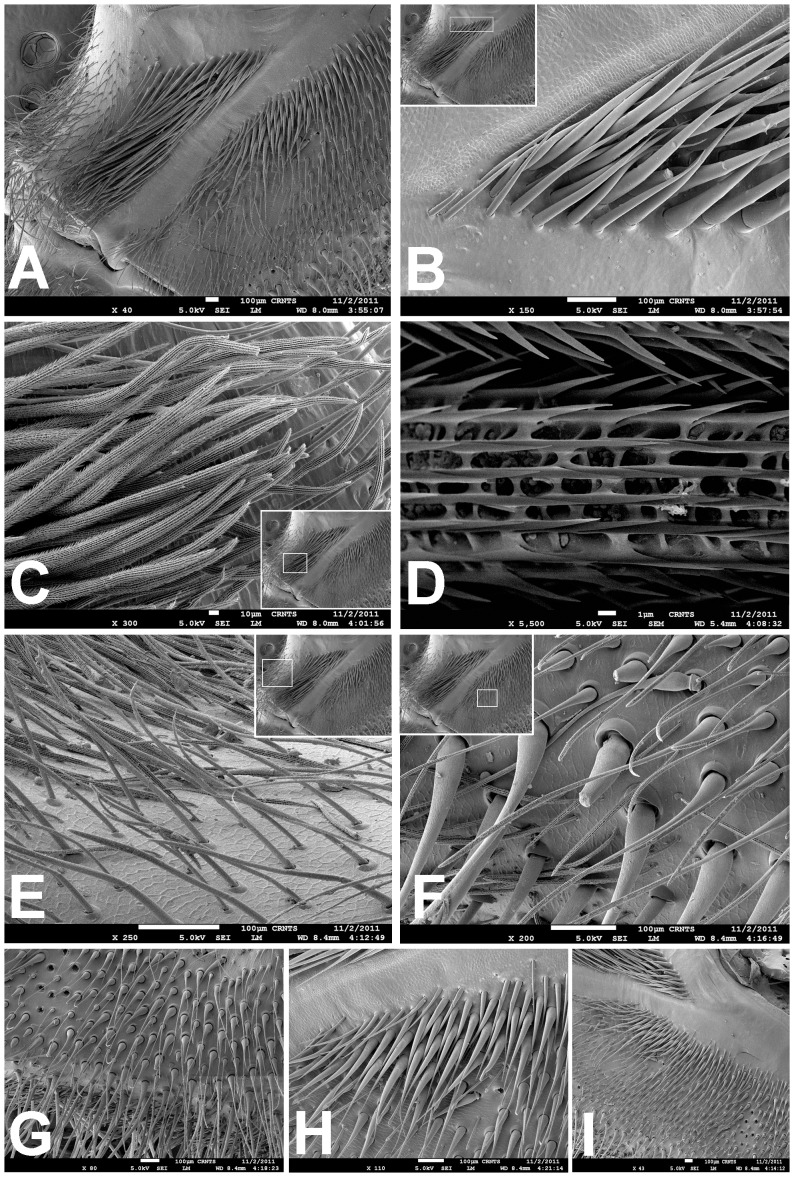
Scanning electron micrograph of *Heterophrictus raveni* **sp. nov.** female paratype (ZSI/WRC/AR/419), coxa II: A. Coxa of leg II prolateral view showing stridulatory setae; B. Basal half of horizontally aligned long pilose setae below coxal suture; C. Distal half of horizontally aligned long pilose setae; D. Ultra-structure of the surface texture of long pilose setae; D. Short pilose setae in posterior distal region of coxa of leg II; 31. Vertically aligned pyriform setae above coxal suture of leg II; E. Vertically aligned pyriform setae above coxal suture of leg II with curved tips; F. Vertically aligned pyriform setae above coxal suture of leg II basal region; G. Junction of coxal suture of leg II.

**Distribution:** Africa- Angola, Congo, Democratic Republic, Ethiopia, Gabon, Guinea, Kenya, Nigeria, Tanzania; Madagascar, Mauritius; United Arab Emirates- Yemen and associated gulf islands and India (Figure 2) [31].

#### *Heterophrictus* Pocock, 1900

*Heterophrictus* Pocock 1900∶180, type species *Heterophrictus milleti* Pocock 1900, by monotypy; Guadanucci 2011∶524.

*Plesiophrictus* Pocock 1899∶749 (In part: *P. blatteri* Gravely 1935).

*Neochilobrachys* Gravely 1935∶83.

**Type species:** *Heterophrictus milleti* Pocock 1900.

**Species included:** *Heterophrictus aareyensis* **sp. nov.**, *H. blatteri* **new combination**, *H. milleti*, *H. raveni* **sp. nov.**

**Diagnosis:** males and females of *Heterophrictus* differ from those of other Theraphosidae genera (except for the Eumenophorinae & Theraphosinae Thorell, 1870) by possessing stridulatory setae between the coxae of legs I–II or I–IV (Fig. 13). It differs from Eumenophorinae genera by having the scopulae of tarsi of leg III and IV divided by a band of short spike setae instead of normal setae (Fig. 35) and possessing pilose as well as pyriform setae on prolateral coxa (Fig. 13). Differs from Theraphosinae in lacking urticating setae. Females differ from all other Eumenophorinae genera (except for *Neoheterophrictus* and *Sahydroaraneus* **gen. nov.**) by possessing rastellum on the prolateral border of chelicerae. Differs from *Neoheterophrictus* and *Sahydroaraneus* **gen. nov.** by lacking tibial spur, by having the tarsal scopulae of leg III–IV divided by a band of short spike setae and in the males possessing a cluster of spiniform setae on the retrolateral basal region of tibia I (Fig. 7). Females differ from *Neoheterophrictus* and *Sahydroaraneus* **gen. nov.** in possessing spermathecal stalks with equal width (diameter) throughout with a single lobe on each stalk (Fig. 3D) (vs. multi lobbed in *Neoheterophrictus*; spermathecal stalk broader at base, tapering at distal end bearing a bud like single lobe).

**Figure 14:**
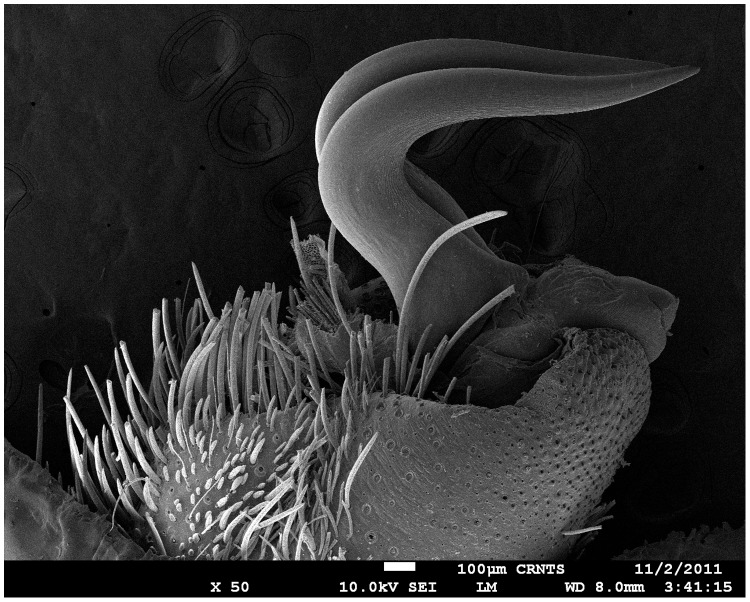
Scanning electron micrograph showing tarsal claws on leg IV of paratype female *Heterophrictus raveni* sp. nov. (ZSI/WRC/AR/419).

**Figure 15:**
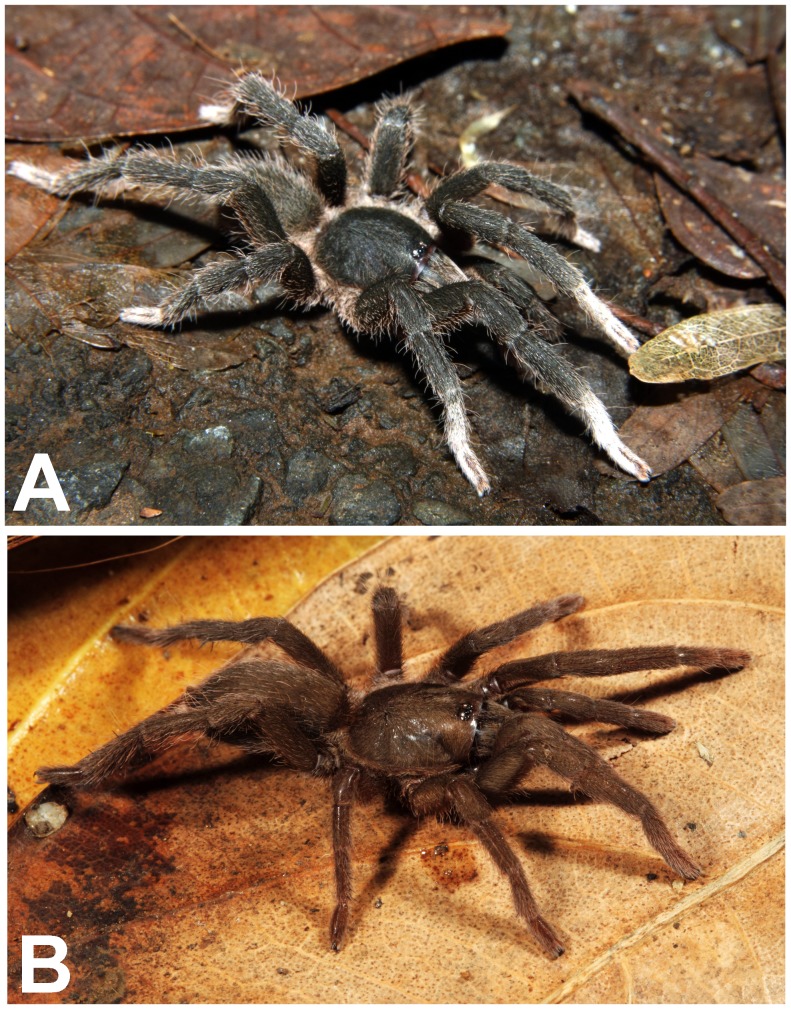
*Heterophrictus aareyensis* **sp. nov.** A. male holotype (ZSI/WRC/AR/420) in life, photo by Rajesh Sanap; B. female paratype BNHS Sp- 85 in life, photo by Zeeshan Mirza.

**Figure 16:**
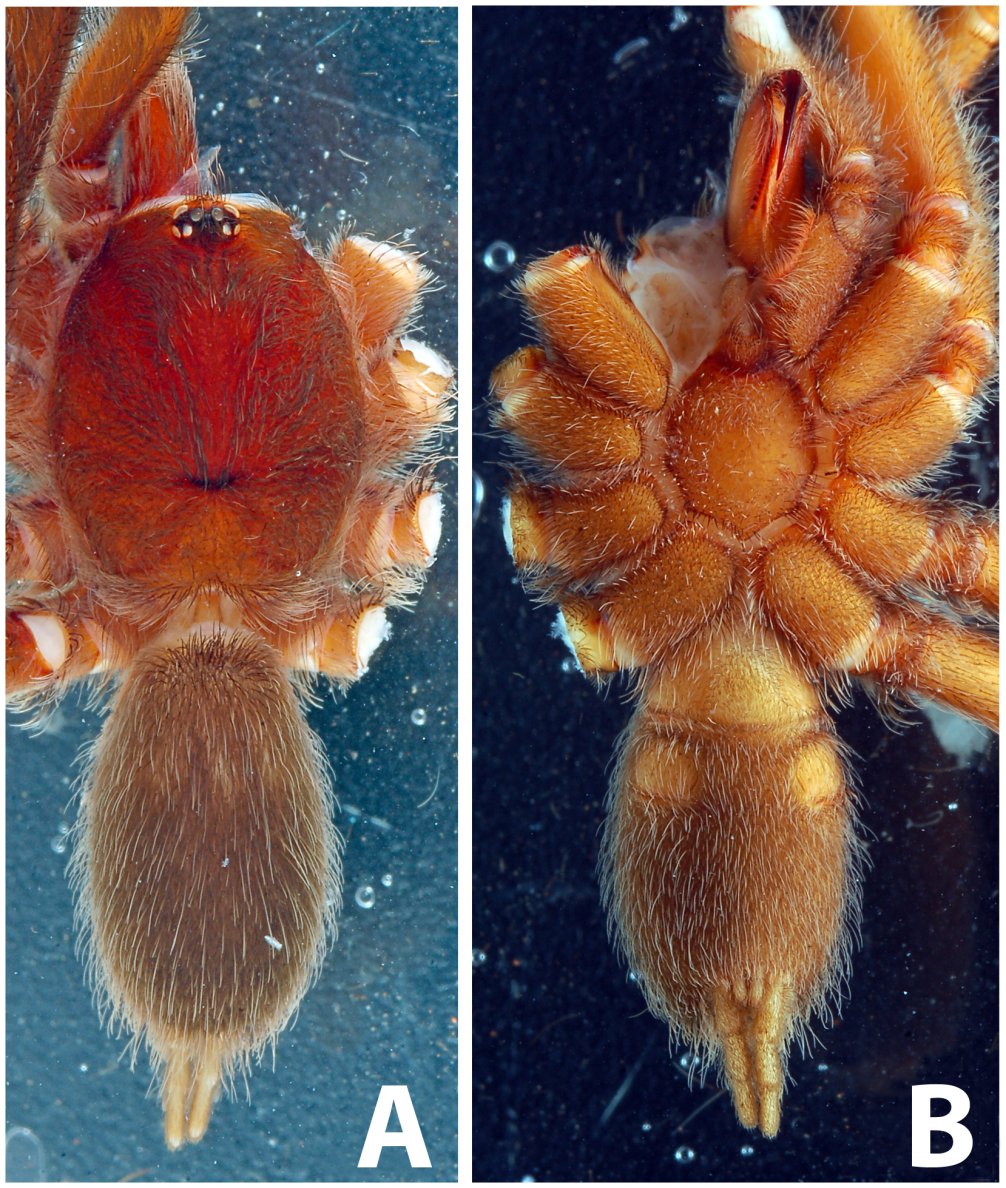
*Heterophrictus aareyensis* sp. nov. male holotype (ZSI/WRC/AR/420). A. Cephalothorax and abdomen, dorsal view; B. Sternum, labium, maxillae, abdomen and chelicerae, ventral view.

**Figure 17:**
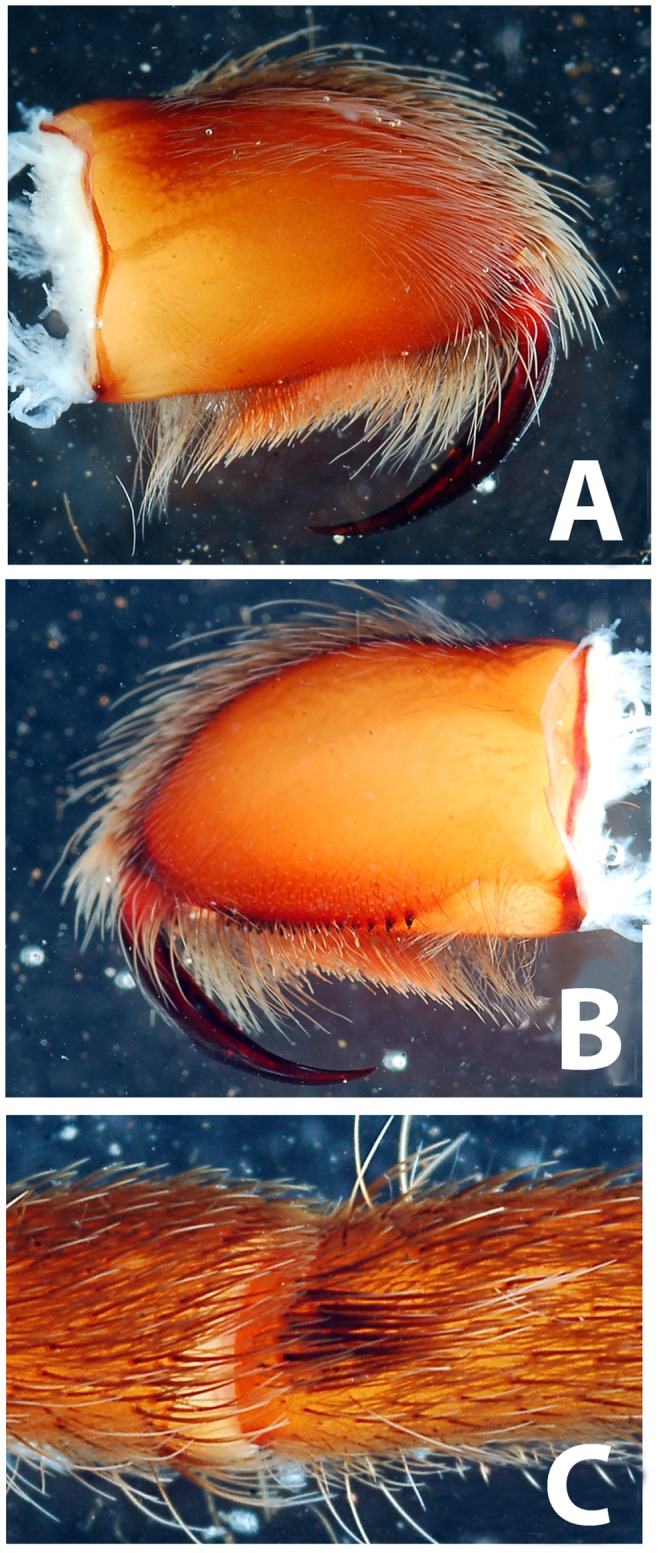
*Heterophrictus aareyensis* sp. nov. male holotype (ZSI/WRC/AR/420). A. Chelicerae retrolateral view; B. Chelicerae prolateral view; C. Cluster of spiniform setae tibia I.

**Figure 18:**
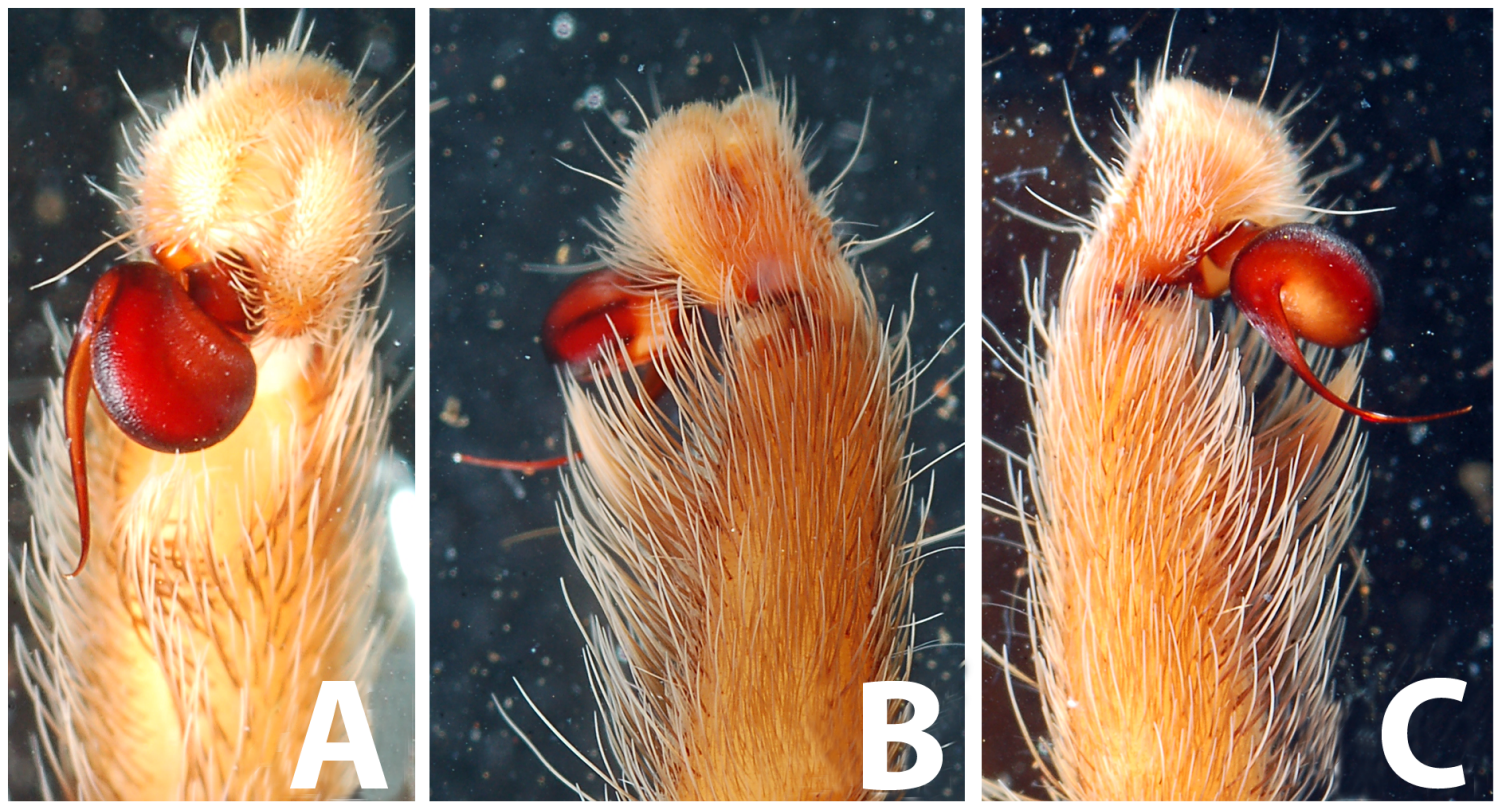
*Heterophrictus aareyensis* sp. nov. male holotype (ZSI/WRC/AR/420). A. palp bulb dorsal view; B. palp bulb prolateral view; C. palp bulb retrolateral view.

**Figure 19:**
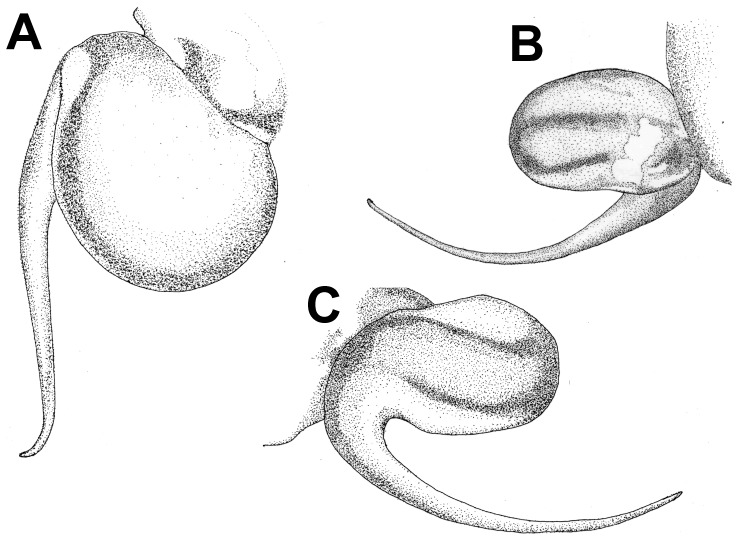
*Heterophrictus aareyensis* sp. nov. male holotype (ZSI/WRC/AR/420). A. Palp bulb dorsal view; B. Palp bulb prolateral view; C. Palp bulb retrolateral view.

**Figure 20:**
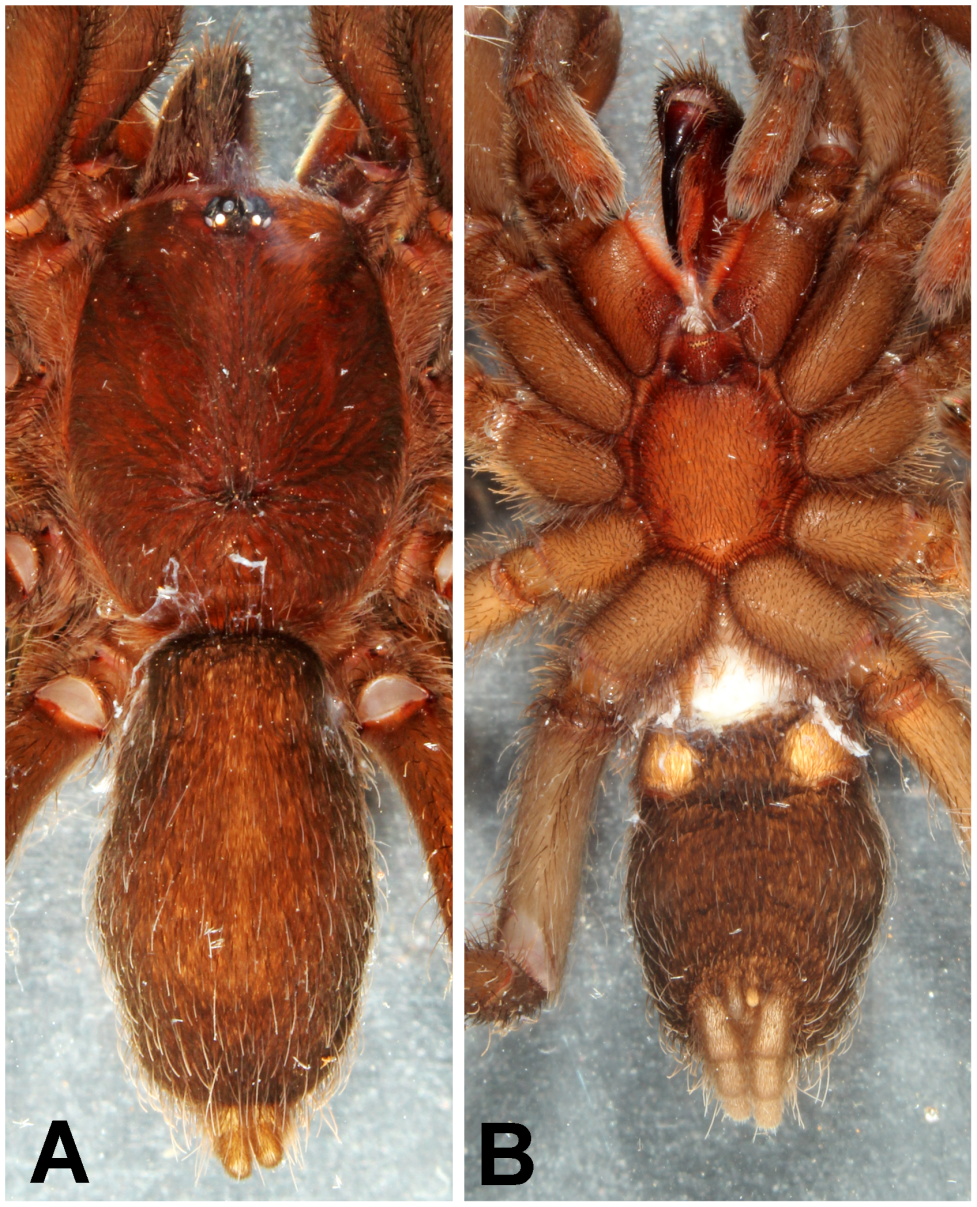
*Heterophrictus aareyensis* sp. nov. female paratype (BNHS SP-85). A. Cephalothorax and abdomen, dorsal view; B. Sternum, labium, maxillae, abdomen and chelicerae, ventral view.

**Figure 21:**
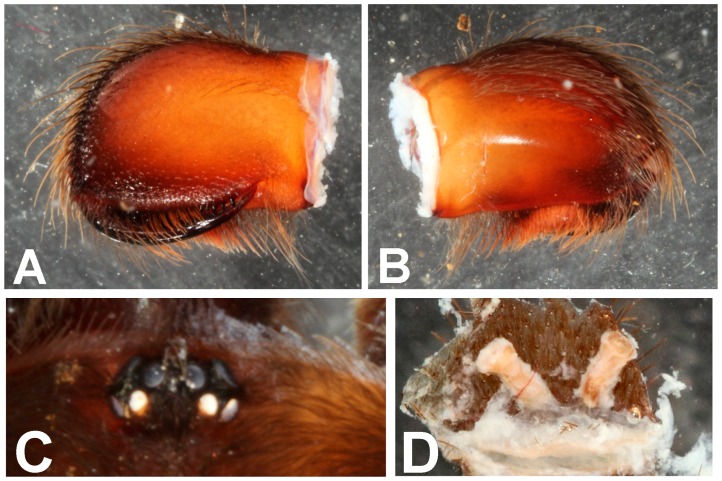
*Heterophrictus aareyensis* sp. nov. female paratype (BNHS SP-85). A. Chelicerae prolateral view; B. chelicerae retrolateral view; C. eye; D. spermathecae.

**Figure 22:**
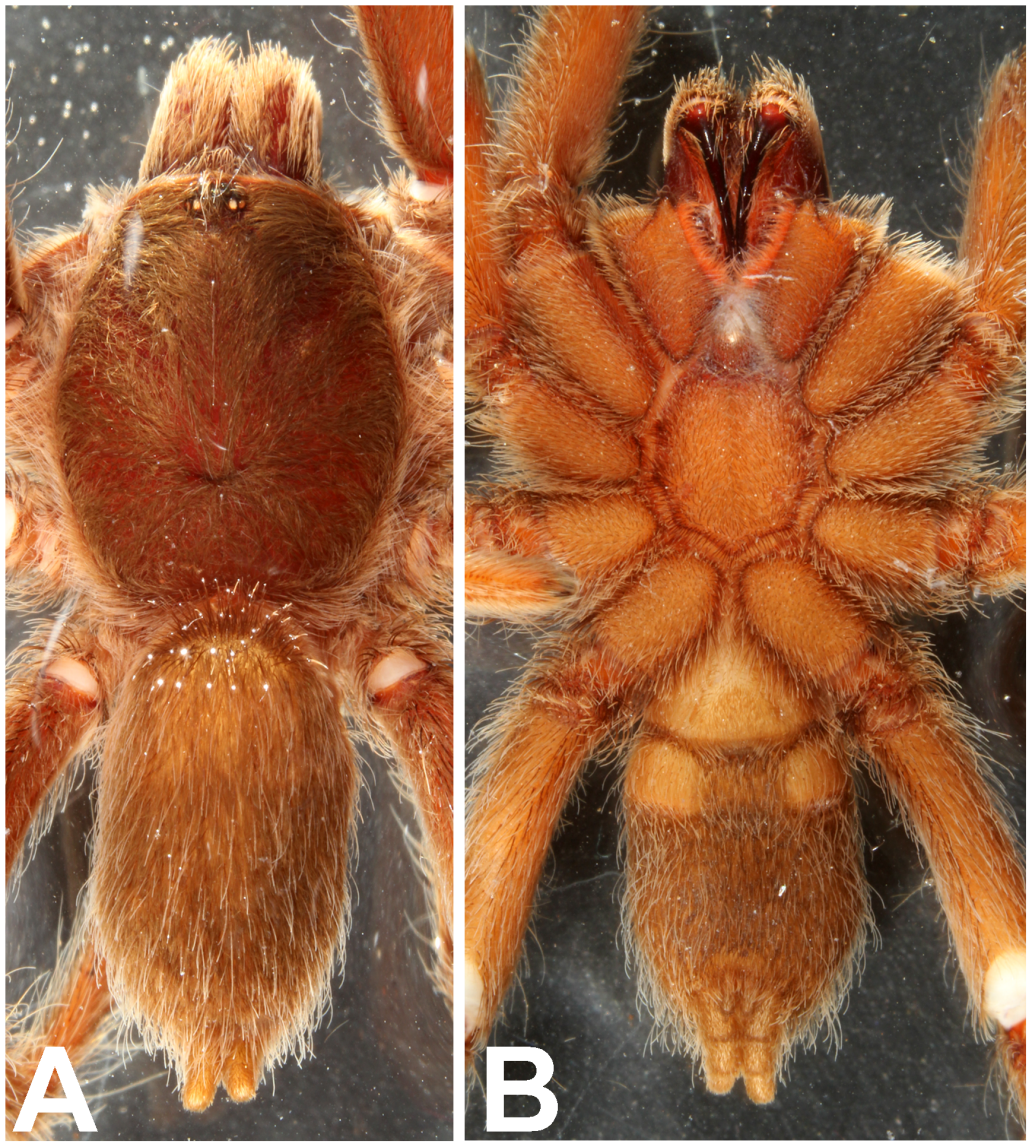
*Heterophrictus blatteri*, male (BNHS SP-86). A. Cephalothorax and abdomen, dorsal view; B. Sternum, labium, maxillae, abdomen and chelicerae, ventral view.

**Figure 23:**
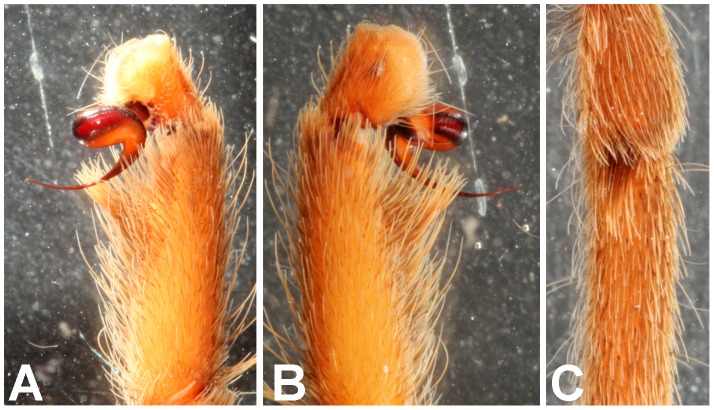
*Heterophrictus blatteri*, male (BNHS SP-86). A. palp bulb prolateral view; B. palp bulb retrolateral view; C. spike setae on tibia I.

**Figure 24:**
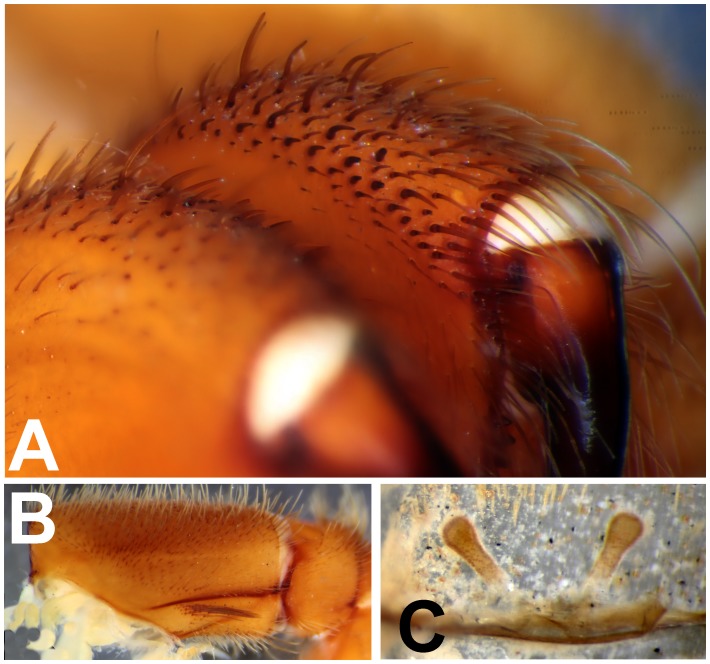
*Heterophrictus blatteri*, female (BMNH 16.5.2.15). A. Chelicerae prodorsal view; A. coxa leg II, prolateral view; B. Spermathecae.

**Figure 25:**
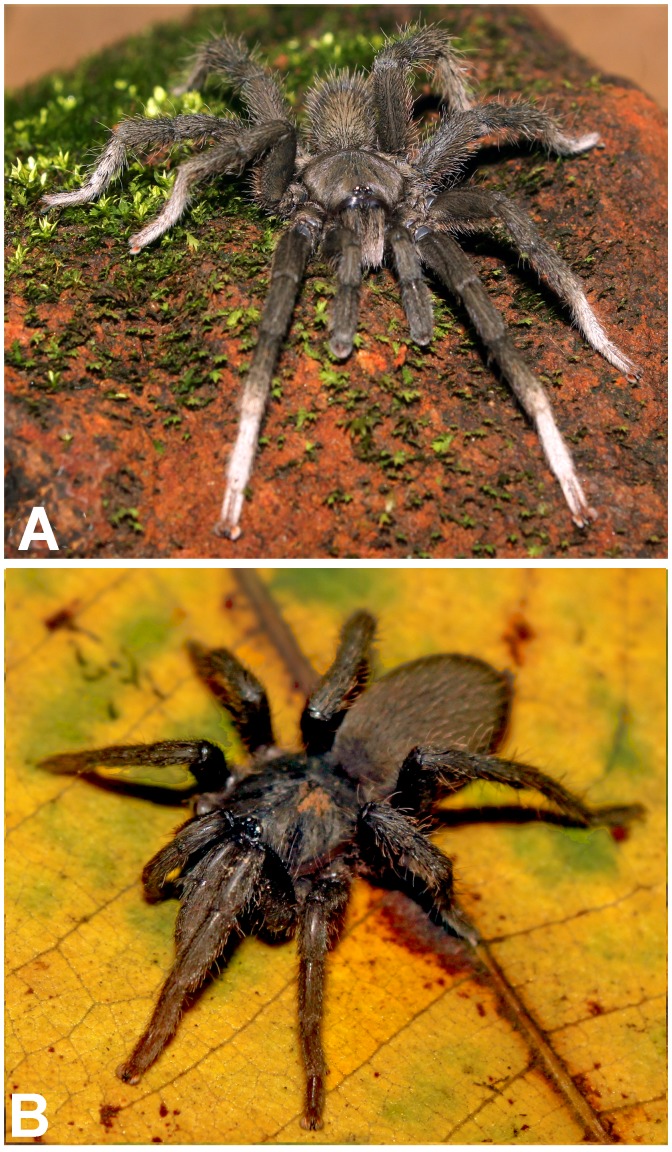
*Neoheterophrictus smithi* sp. nov. male holotype (ZSI/WRC/AR/421). A. *Neoheterophrictus smithi* **sp. nov** male holotype in life; B. *Neoheterophrictus smithi* **sp. nov** female paratype in life, photos by Harshal Bhosale.

**Figure 26:**
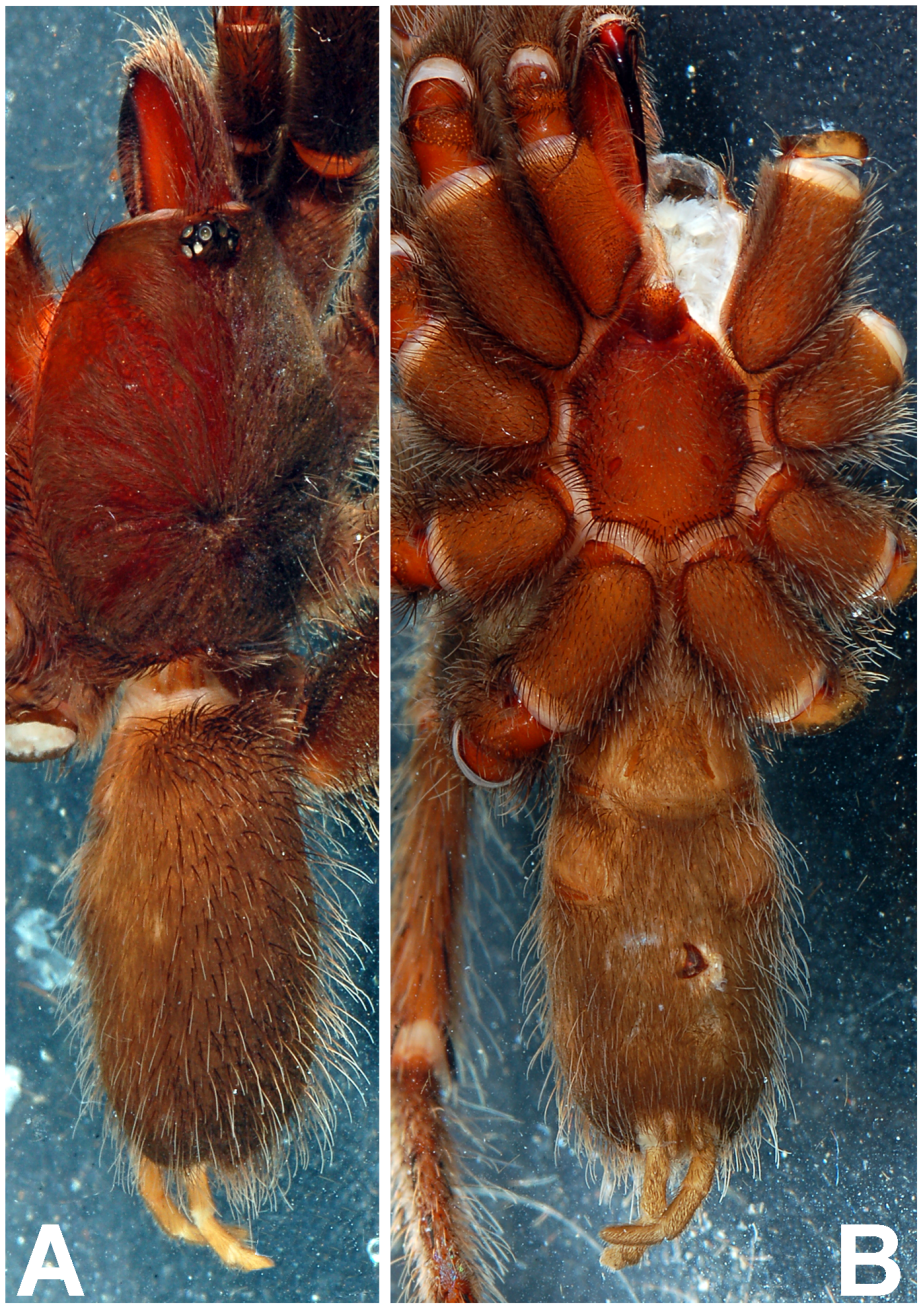
*Neoheterophrictus smithi* sp. nov. male holotype (ZSI/WRC/AR/421). A. Cephalothorax and abdomen, dorsal view; B. Sternum, labium, maxillae, abdomen and chelicerae, ventral view.

**Figure 27:**
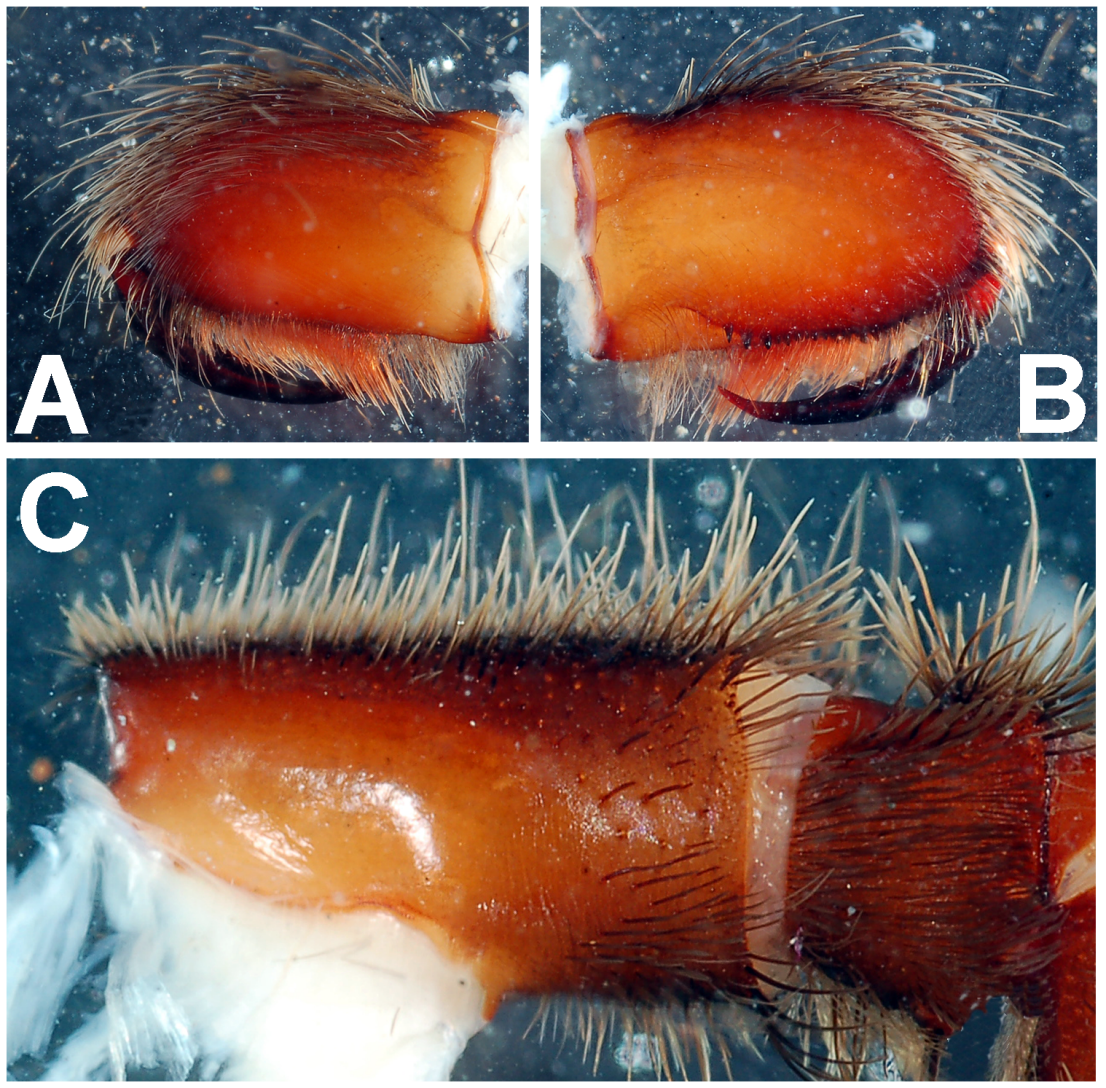
*Neoheterophrictus smithi* sp. nov. male holotype (ZSI/WRC/AR/421). A. Chelicerae retrolateral view; B. Chelicerae prolateral view; C. Retrolateral view of maxilla showing stridulatory setae aligned in a dorso-ventral series.

**Figure 28:**
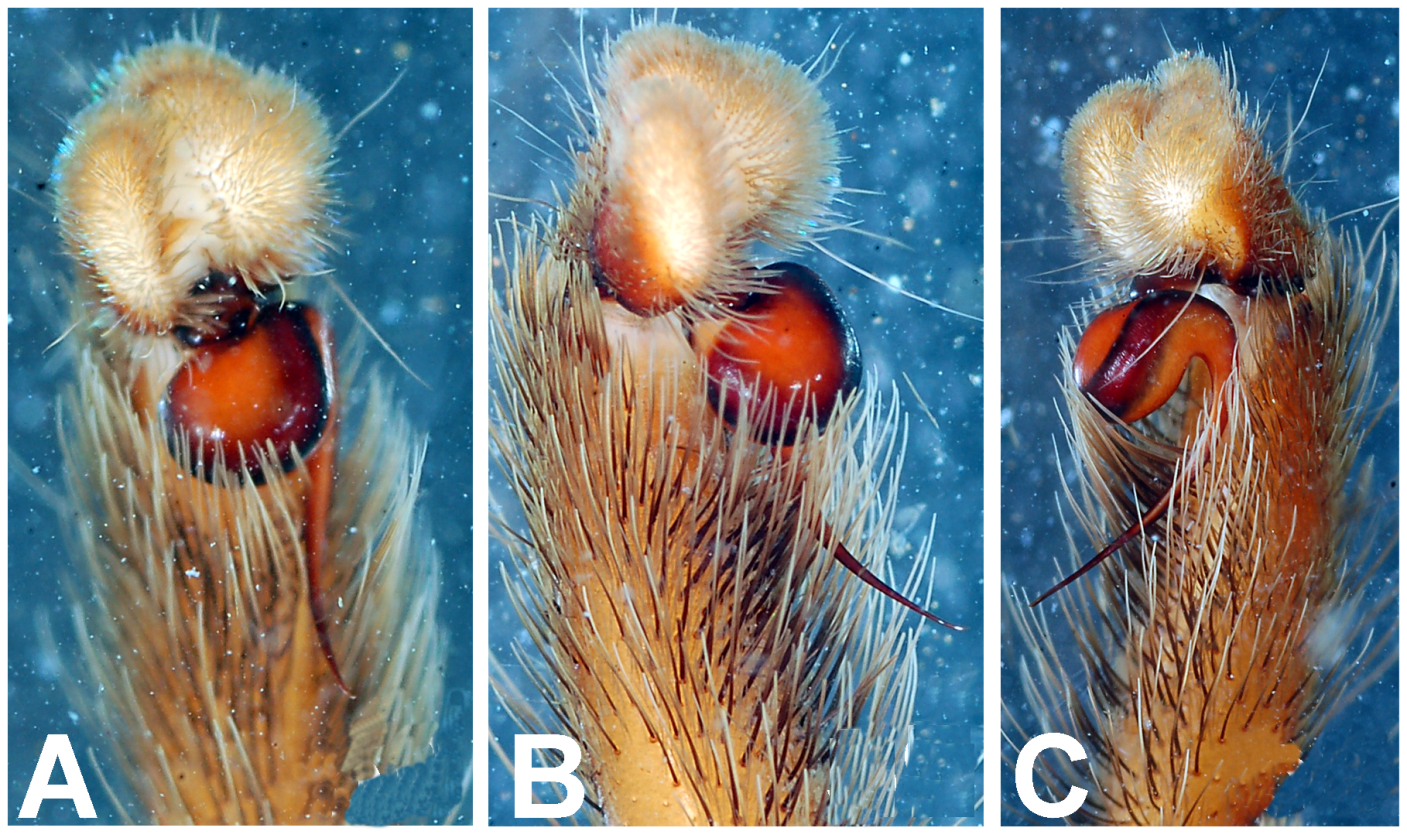
*Neoheterophrictus smithi* sp. nov. male holotype (ZSI/WRC/AR/421). A. Palp bulb dorsal view; B. Palp bulb prolateral view; C. palp bulb retrolateral view.

**Figure 29:**
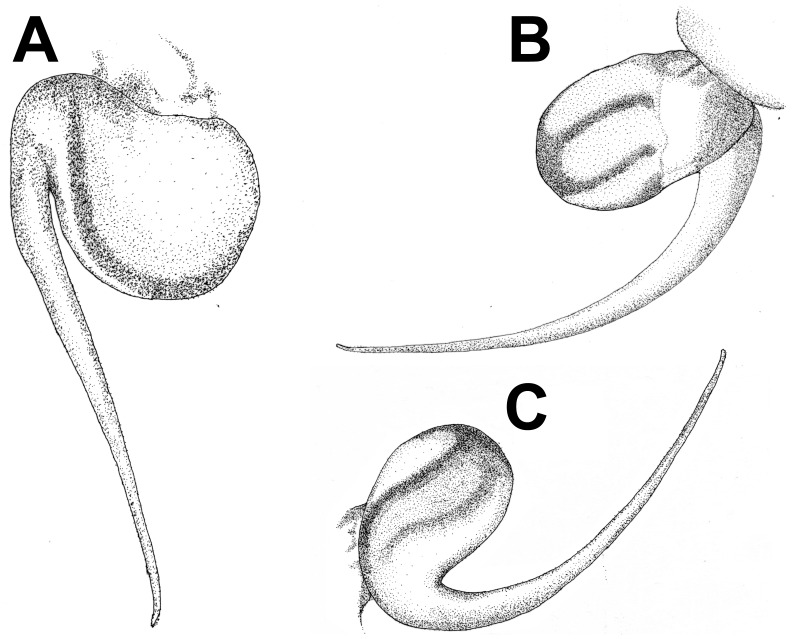
*Neoheterophrictus smithi* sp. nov. male holotype (ZSI/WRC/AR/421). A. Palp bulb dorsal view; B. Palp bulb retrolateral view; C. Palp bulb prolateral view.

**Figure 30:**
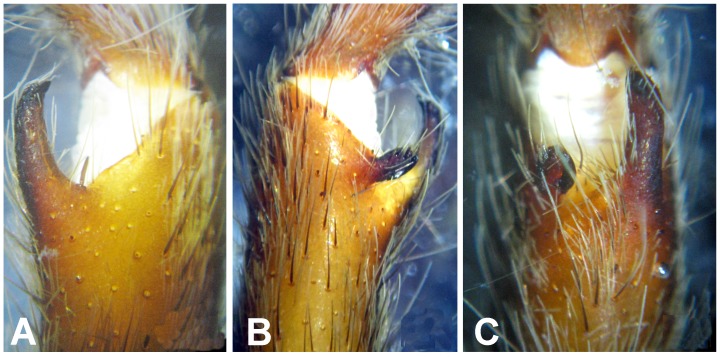
*Neoheterophrictus smithi* sp. nov. male holotype (ZSI/WRC/AR/421). A. Tibial apophysis retrolateral view; B. Tibial apophysis prolateral view; C. Tibial apophysis ventral view.

**Figure 31:**
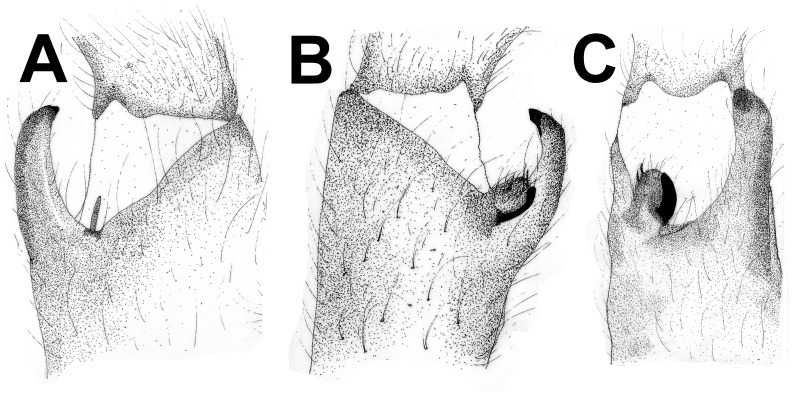
*Neoheterophrictus smithi* sp. nov. male holotype (ZSI/WRC/AR/421). A. Tibial apophysis retrolateral view; B. Tibial apophysis prolateral view; C. Tibial apophysis ventral view.

**Figure 32:**
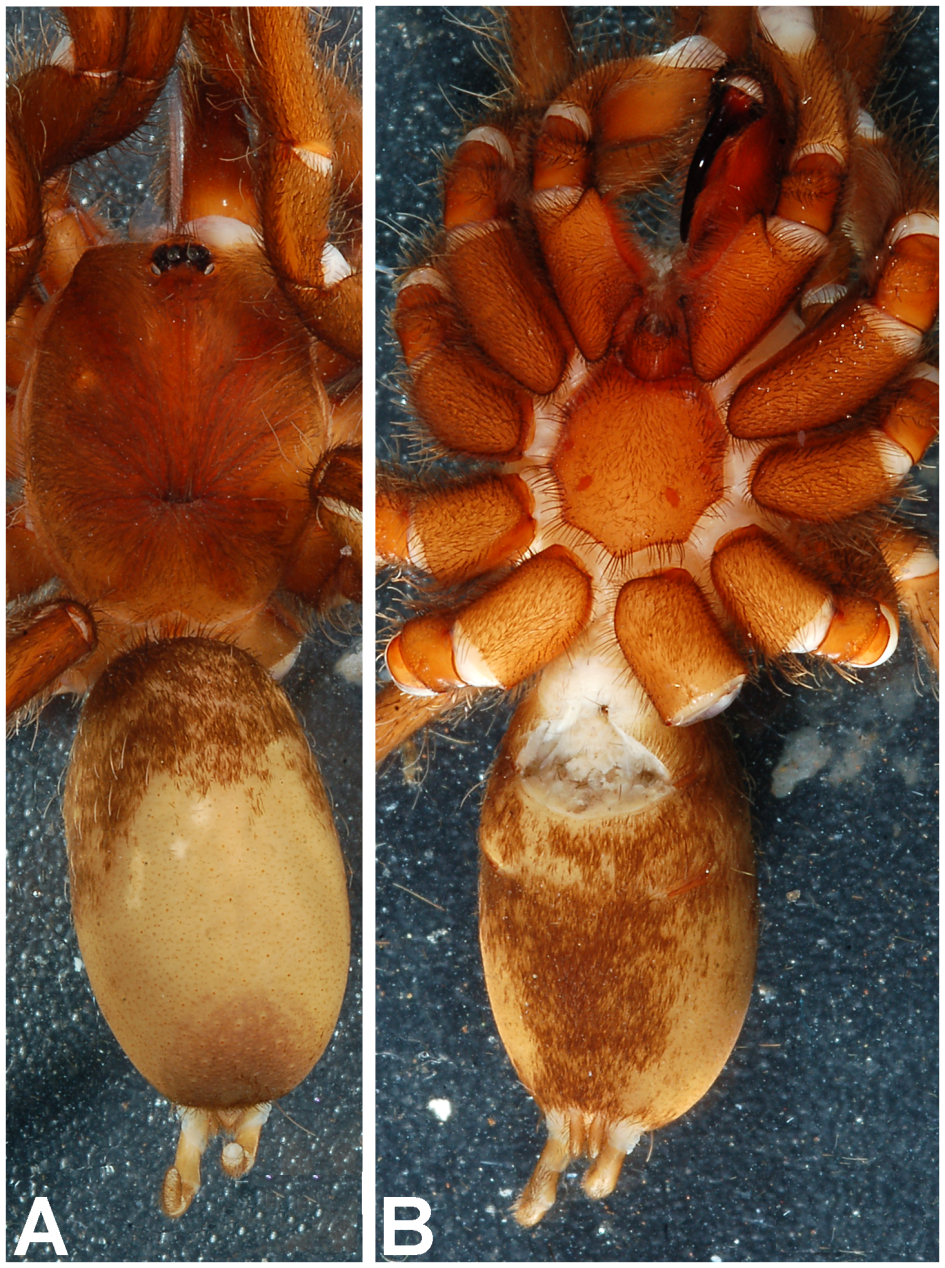
*Neoheterophrictus smithi* sp. nov. female (ZSI/WRC/AR/422). A. Cephalothorax and abdomen, dorsal view; B. Sternum, labium, maxilla, chelicerae and abdomen ventral view.

**Figure 33:**
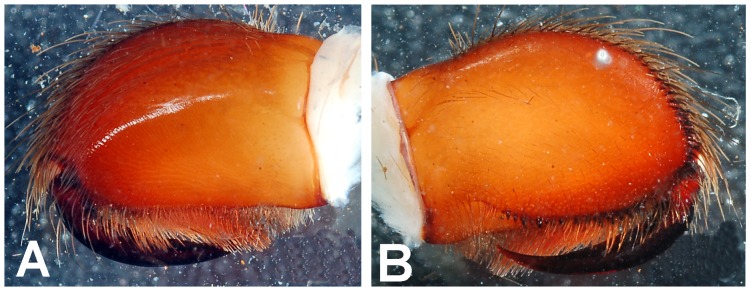
*Neoheterophrictus smithi* sp. nov. female (ZSI/WRC/AR/422). A. Chelicerae retrolateral view; B. Chelicerae prolateral view.

**Figure 34:**
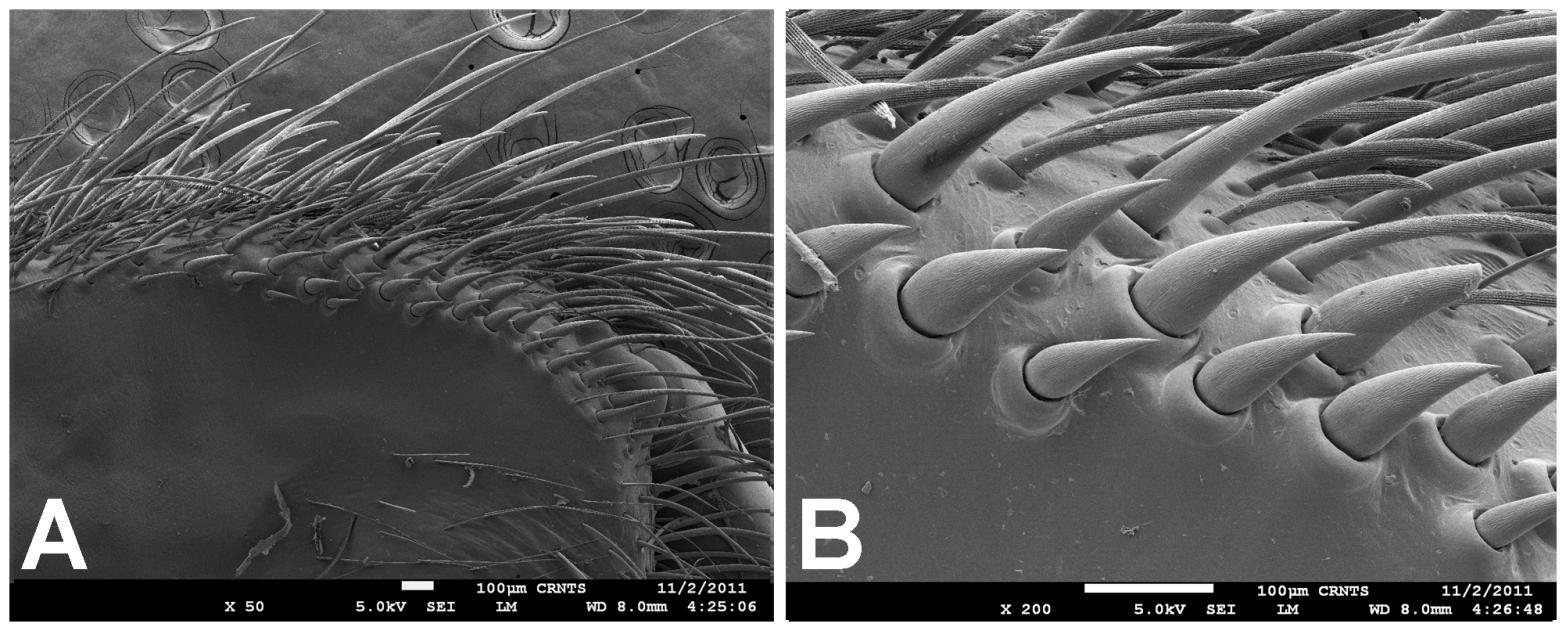
Scanning electron micrograph of *Neoheterophrictus smithi* sp. nov. female (ZSI/WRC/AR/422). A. Cheliceral prolateral broader showing rastellum inter-mixed with normal setae; B. Prolateral cheliceral border showing stout spines.

**Figure 35:**
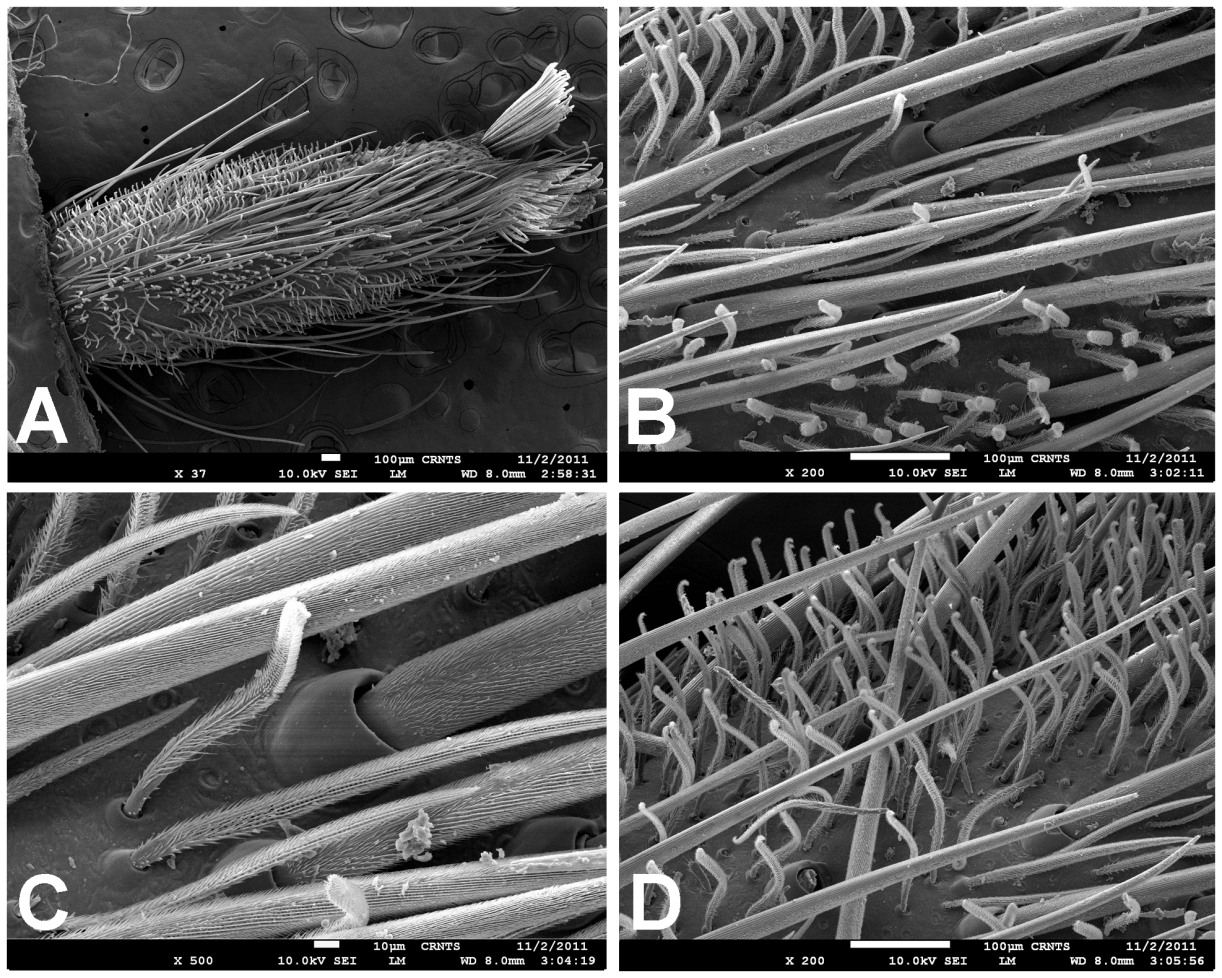
Scanning electron micrograph of *Neoheterophrictus smithi* sp. nov. female (ZSI/WRC/AR/422). A. Ventral view of tarsus showing dividing spike setae; B. Base of spike setae; C. Base of spike setae; D. Spike setae inter-mixed with scopulae setae.

**Description:** Medium sized spiders 14–26. Carapace ovate, hirsute, with two clear (setae-less) bands on both sides of the caput. Caput low. Fovea slightly procurved. Eye group sub-quadrate to wider than long, ocular tubercle well defined. Clypeus narrow. Chelicerae normal, with 16–21 teeth on promargin of furrow, basomesally 24–50 small teeth. Rastellum in the form of small stout spines on the prolateral cheliceral border in females. Labium wider than long. Labiosternal grove shallow with two distinct mounds. Cuspules 25–30 in the sub-apical region of the labium. Maxillae longer than wide, overall setose, prolateral anterior angle distinctly produced, 100–150 cuspules distributed along proximal prolateral angle. Serrula absent. Sternum as long as wide, sigilla small oval, submarginal. Stridulatory setae below coxal suture with horizontally aligned thick pilose setae. Base of setae with fine lines on the surface eventually turns pilose in nature (Fig. 13A–I). Below these are sparsely arranged thin pilose setae. Bellow suture with numerous vertically aligned pyriform setae (Fig. 31–34). Pyriform setae gradually traosform into filiform setae ending in a curve (Fig. 13F–G). Several short setae with scoop like tips intermixed with pyriform setae. Legs moderately stout, hirsute, spines present except on femora & coxa. Paired claws on legs without dentition (Fig. 14) and claw tufts well-developed. All tarsi with scopula, metatarsi with ¼ scopulate. Abdomen hirsute, without pattern. PMS well-developed; PLS, apical segment digitiform. Males lack tibial apophysis. A cluster of spiniform setae present on the retrolateral basal region of the tibia I (Fig. 7A–C). Palpal organ pyriform, with filiform embolus, ending in a scoop with a sharp tip, lacking keels. Spermathecae in the form of twin seminal receptacles with the apical end slightly bulbous and rounded.

#### *Heterophrictus milleti* Pocock 1900

*Plesiophrictus milleti* Raven 1985∶154.

Fig. 3A–D

**Material examined:** three female specimens from type locality collected by Millet from Nashik in the collection of NHM. Registration numbers not available. (Ex-dry specimens).

**Remark:** Detailed description provided by Guadanucci [19].

#### *Heterophrictus raveni* Mirza & Sanap, sp. nov

 urn:lsid:zoobank.org:act:E6E2B6C7-0E6C-40EC-8128-6FCB4F4DF13B.

Fig. 4–14, 45 A & B

**Figure 36:**
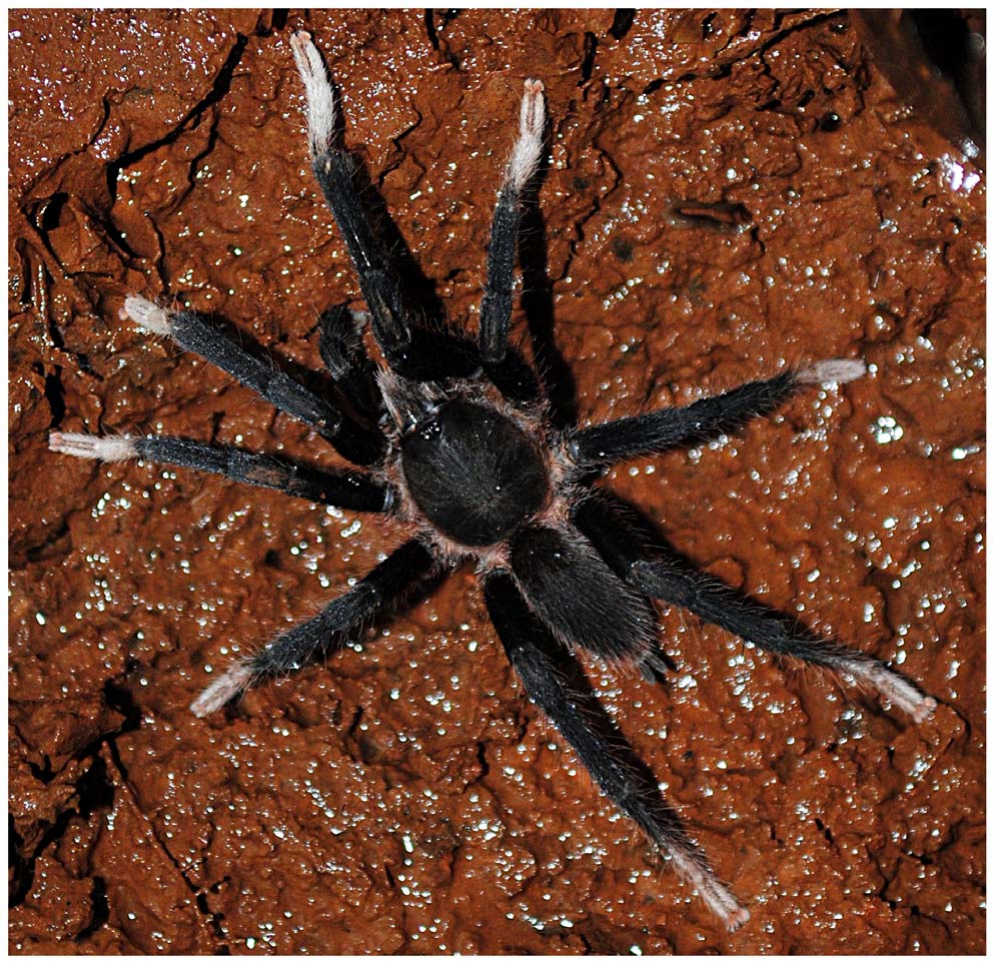
*Neoheterophrictus amboli* sp. nov. male holotype (ZSI/WRC/AR/423) in life. Photo by Aditya Malgaonkar.

**Figure 37:**
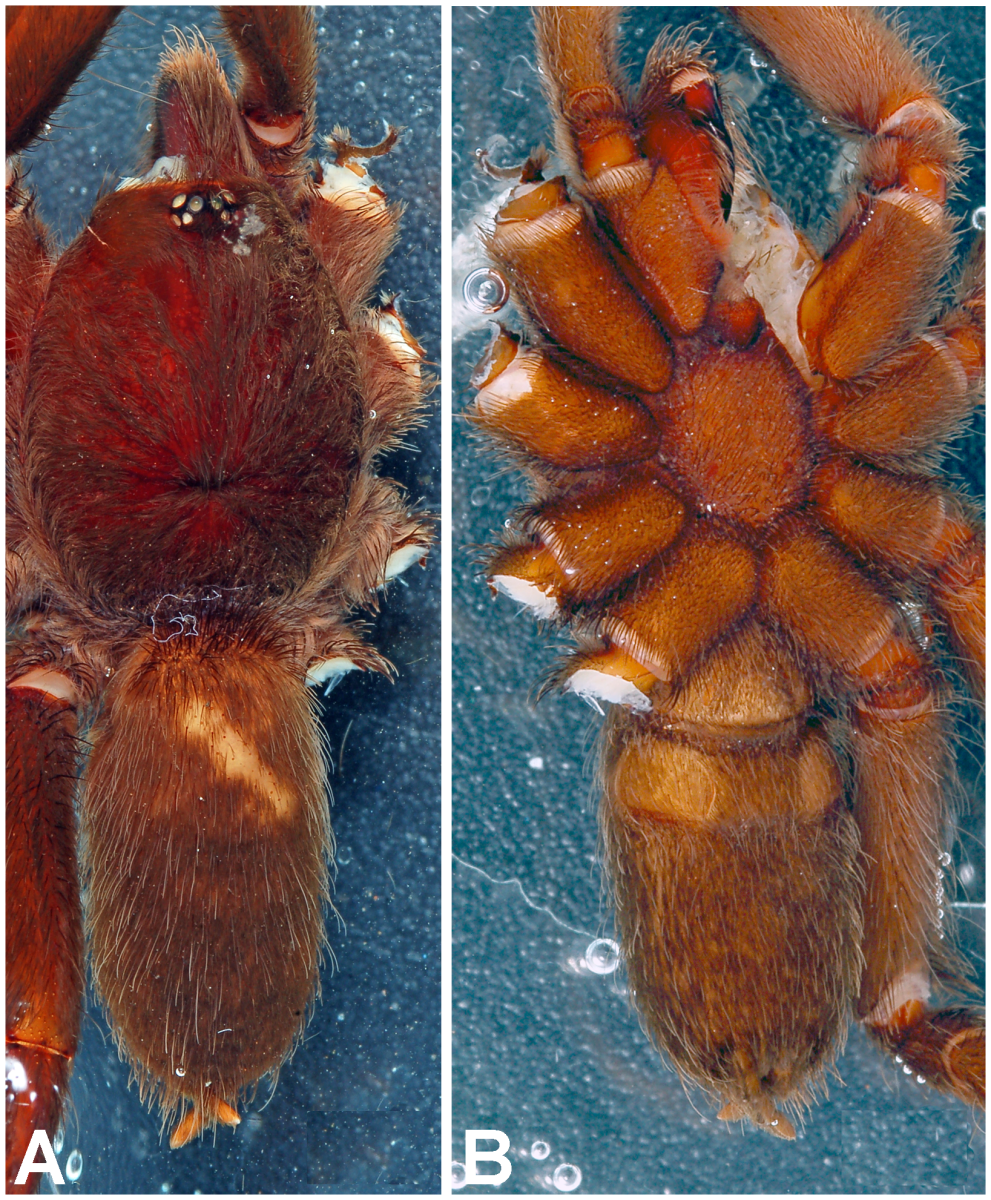
*Neoheterophrictus amboli* sp. nov. male holotype (ZSI/WRC/AR/423). A. Cephalothorax and abdomen, dorsal view; B. Sternum, labium, maxillae, abdomen and chelicerae, ventral view.

**Figure 38:**
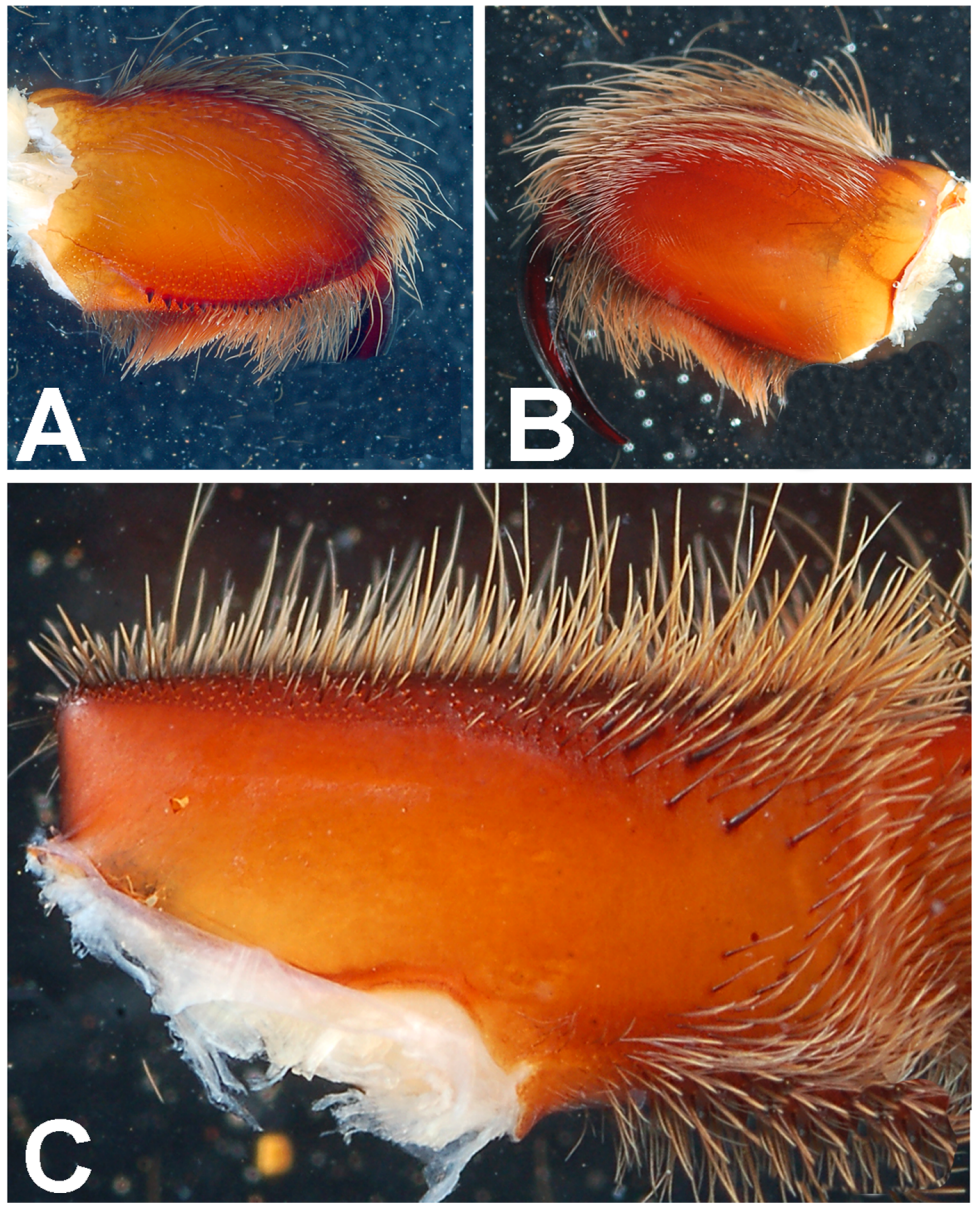
*Neoheterophrictus amboli* sp. nov. male holotype (ZSI/WRC/AR/423). A. Chelicerae prolateral view; B. Chelicerae retrolateral view; C. Maxilla retrolateral view showing stridulatory setae intermixed with normal setae between palp-I on the retrolateral basal region.

**Figure 39:**
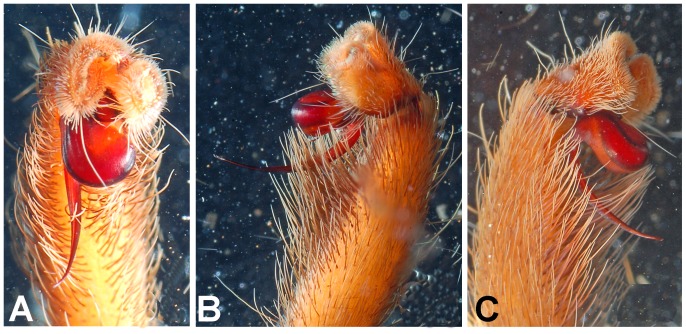
*Neoheterophrictus amboli* sp. nov. male holotype (ZSI/WRC/AR/423) palp bulb. A. dorsal view; B. Prolateral view; C. Rretrolateral view.

**Figure 40:**
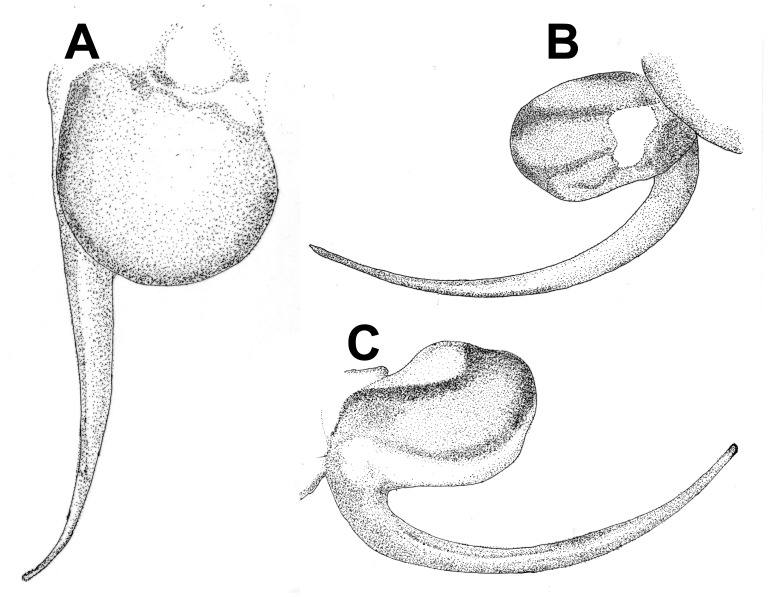
*Neoheterophrictus amboli* sp. nov. male holotype (ZSI/WRC/AR/423) palp bulb. A. dorsal view; B. Prolateral view; C. Retrolateral view.

**Figure 41:**
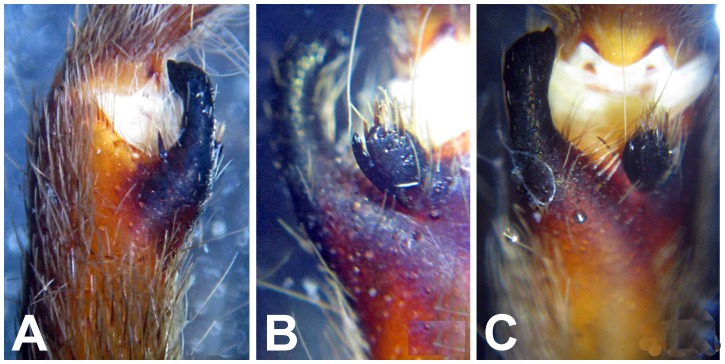
*Neoheterophrictus amboli* sp. nov. male holotype (ZSI/WRC/AR/423) tibial apophysis. A. retrolateral view; B. prolateral view; C. Ventral view.

**Figure 42:**
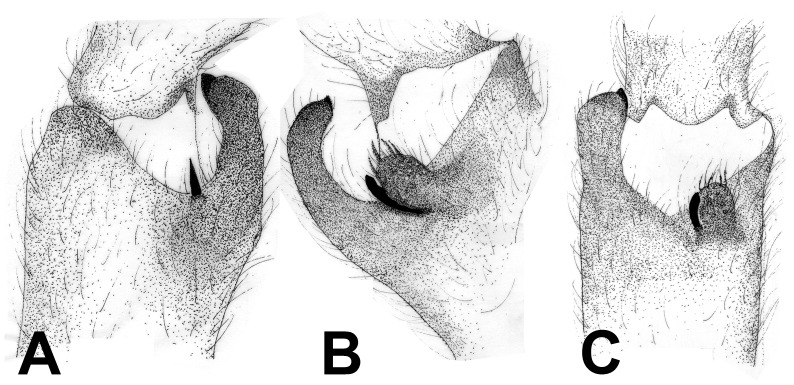
*Neoheterophrictus amboli* sp. nov. male holotype (ZSI/WRC/AR/423) tibial apophysis. A. Retrolateral view; B. Prolateral view; C. Ventral view.

**Figure 43:**
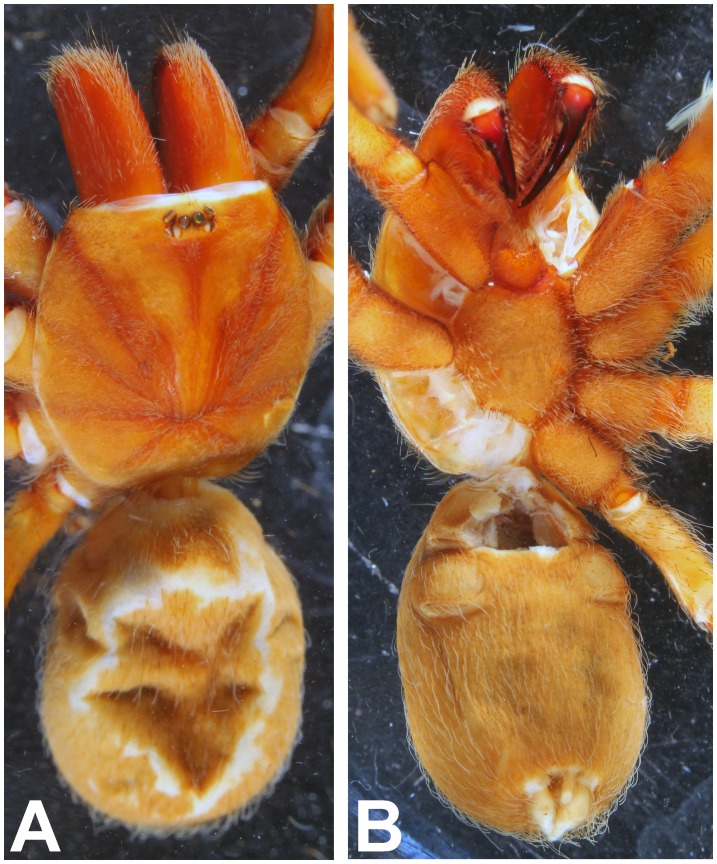
*Neoheterophrictus bhori* female (Type BMNH 16.5.2.16). A. Cephalothorax and abdomen, dorsal view; B. Sternum, labium, maxillae, abdomen and chelicerae, ventral view.

**Figure 44:**
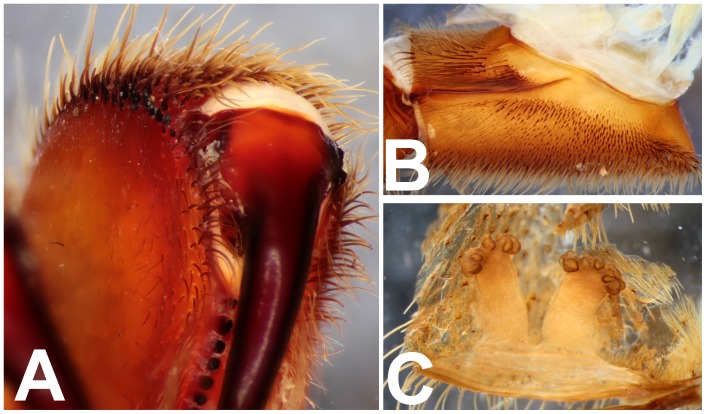
*Neoheterophrictus bhori* female (Type BMNH 16.5.2.16). A. Chelicerae pro-dorsal view B. coxa leg III, prolateral view; C. spermathecae.

**Figure 45:**
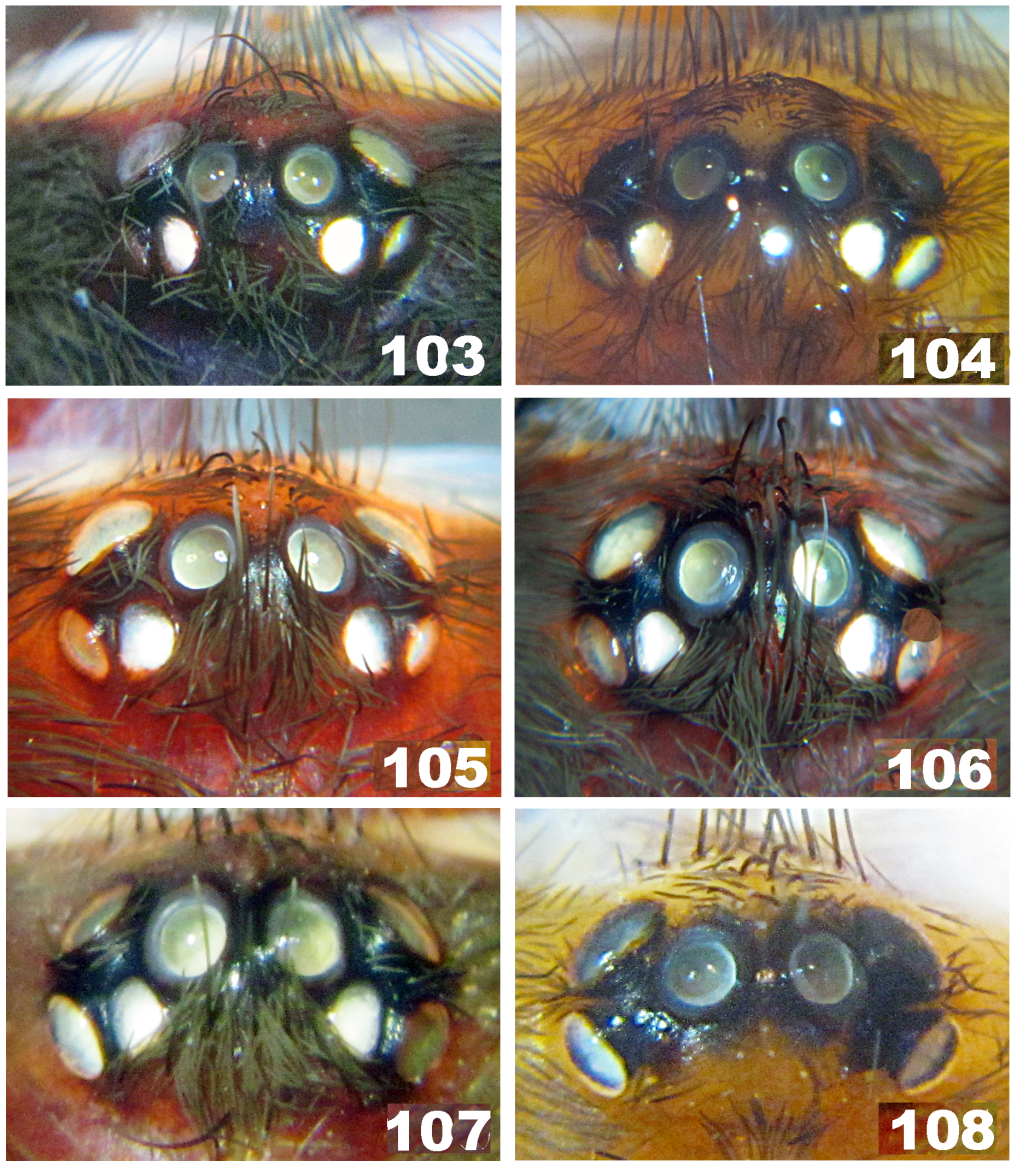
Eyes in *Heterophrictus* and *Neoheterophrictus*. A. *Heterophrictus raveni* **sp. nov.** holotype male; B. *Heterophrictus raveni* **sp. nov.** paratype female; C. *Heterophrictus aareyensis* **sp. nov.** holotype male; D. *Neoheterophrictus amboli* **sp. nov.** holotype male; E. *Neoheterophrictus smithi* **sp. nov.** holotype male; F. *Neoheterophrictus smithi* **sp. nov.** female.

**Type specimens: INDIA–** holotype, male, 15.viii.2011, elevation 800 m, Phalkewadi near Bhimashankar Wildlife Sanctuary, Pune District, Maharashtra (19° 3′43.47″N, 73°34′3.63″E), coll. Sushil Chikane & Harshal Bhosale, ZSI/WRC/AR/418. Paratype, female, 10.v.2011, coll. Rajesh Sanap, Ashish Jadhav & Zeeshan Mirza, ZSI/WRC/AR/419, same data as holotype.

**Diagnosis:** *Heterophrictus raveni* **sp. nov.** males differ from *Heterophrictus aareyensis* **sp. nov.** by being larger in total length, by possessing stridulatory setae on coxae of all legs and having the same more elaborate in *H. raveni* **sp. nov.** The new species possesses more basosomal teeth as competed to *H. aareyensis* **sp. nov.** and PLE are placed in advance of the posterior border of PME giving it a procurved appearance (Fig. 45A).

**Etymology:** The new species is named in honor of Dr. Robert Raven from Queensland Museum, Australia for his immense contribution to the study of mygalomorph spiders of the world.

#### Description of holotype male ZSI/WRC/AR/4187

total length 20.82, carapace 9.8 long, 8.3 wide, chelicerae 5.9 long after dissection. Sternum 3.48 long, 3.68 wide. Abdomen 11.02 long, 6.68 wide. Spinnerets: PMS, 0.52 long, 0.2 wide, 0.32 apart; PLS, 1.06 basal, 0.8 mid, 0.76 distal, total length 2.62; midwidths 0.58, 0.54, 0.4 respectively, apart 0.84.

Carapace (Fig. 5A & B): length to width ratio 1.18; uniform reddish brown, covered with short grey setae, more concentrated towards margins and along interstitial ridges radiating from fovea. Two clear setae-less bands along sides of caput. Fovea equal to ocular width, slightly procurved.

Eyes (Fig. 45A): width to length ratio of ocular group 2.51. Eye diameter: ALE, 0.32; AME, 0.2; PLE, 0.16; PME, 0.18. Distance between eyes: AME–AME, 0.18; PME–PLE, 0.1; AME–ALE, 0.1; PME–PME, 0.46; ALE–PLE, 0.08. Ocular quadrate, 0.58 long, 1.46 wide. MOQ: length, 0.4; front width, 0.64; back width, 0.8. Clypeus narrow and eye tubercle distinct.

Maxilla (Fig. 4B): prolateral face smooth with a few short black setae, retro-face yellowish-red, smooth, glabrous. Front length 2.6, back length 3.4 and 1.74 wide. Cuspules more than 100 in anterior corner in roughly triangular region.

Labium (Fig. 4B): 0.98 long and 1.24 wide with ca. 27 cuspules in band for ¼ of anterior length; labiosternal groove concave with two distinct mounds at labio-sternal grove.

Chelicerae (Figs. 5A–B): prolateral teeth 19 and ca. 40 basosomal teeth; rastellum absent.

Sternum (Fig. 4B): longer than wide, high in center, slopping gradually, covered with short black setae. Posterior edge pointed but not separating coxa IV. Long and short bristles radiate from margin. Pedicel not clearly seen.

Sigilla (Fig. 4B): three pairs, posterior 0.28 diameter, 1.38 apart; 0.34 distance from margin; middle 0.14 diameter, 2.9 apart, 0.16 distance from margin; anterior 0.08 diameter, 2.84 apart, 0.08 distance from margin.

Legs: formula 4123, prograde; morphometry (femur, patella, tibia, metatarsus, tarsus, total): I: 8.2, 4.16, 7.16, 3.94, 3, 26.46. II: 7.92, 3.66, 4.32, 3.88, 2.98, 22.76. III: 7, 3.3, 4.66, 5.02, 2, 21.98. IV: 7.94, 4.18, 6.94, 7.66, 3.66, 30.38. Palp: 4.8, 3.08, 3.94, –, 1.3, 13.12. Midwidths: femora I – IV = 2.78, 2.34, 2.66, 1.92, palp = 1.4; tibiae I–IV = 1.66, 1.26, 1.68, 1.3, palp = 1.48.

Spines: leg I - mt v1, ti v 1; Leg II - mt v 3, ti v 2, p 1; Leg III - mt p 1, r 2, v 5, ti p 1, r 1, v 2, pa p 1; Leg IV - mt p 2, r 2, v 5, ti v 5, r 2. Absent elsewhere.

Spike setae (Fig. 6A & B, 7A–C: a cluster of 16–18 spike setae present on basal region of retrolateral aspect of tibia of leg I.

Leg coxae: prolateral coxa of all legs with a few horizontally aligned pilose spike below coxal suture; above with vertically aligned pyriform setae. Retrolateral face with long black bristles on dorsal retrolateral border. Coxal bases dorsally easily seen from above. Coxae, I–II sloping forward, III–IV sloping backward. Coxa IV widest.

Scopulae: entire on tarsi I & II, undivided; tarsi III divided by a band of 4–5 rows of short spike setae, tarsi IV divided by a band of 7–8 rows of short spike setae. Metatarsi I–IV undivided and ¼ scopulate.

Trichobothria: tarsi I & II 20 clavate and 11–12 long and short filiform; tarsi III 15–16 clavate and 11–13 long and short filiform; tarsi IV 20–21 clavate and 12–14 long and short filiform; palp tarsi with 18 clavate. Trichobothria in two rows throughout tarsi. Metatarsi I–II with 8–10 long and short filiform, metatarsi III & IV with 10–15 long and short filiform.

Abdomen (Figs. 3–4): cuticle not exposed dorsally and ventrally; covered with fine layer of dark brown setae intermixed with long silver setaes.

Palp bulb (Figs. 6C–E & 8A–C): smooth, lacking keels, pyriform. Embolus filiform, stout at base gradually tapering in to a fine point. Tip directed forward when viewed at rest.

**Description of female paratype ZSI/WRC/AR/419 (Fig. 9A):** total length 26.22, carapace 11.68 long, 8.98 wide, chelicerae 6.96 long after dissection. Sternum 4.4 long, 4.2 wide. Abdomen 14.54 long, 10.3 wide. Spinnerets: PMS, 0.7 long, 0.46 wide, 0.3 apart; PLS, 1.24 basal, 0.86 middle, 1.28 distal, total length 3.38; midwidths 0.8, 0.7, 0.42 respectively, apart 1.02.

Carapace (Fig. 10A): length to width 1.3; uniform reddish brown, covered with short brown setae, more concentrated towards margins and along interstitial ridges radiating from fovea. Fovea equal to ocular width, slightly procurved.

Eyes (Fig. 45B): width to length ratio of ocular group 2.6. Eye diameter: ALE, 0.34; AME, 0.28; PLE, 0.2; PME, 0.22. Distance between eyes: AME–AME, 0.18; PME–PLE, 0.1; AME–ALE, 0.14; PME–PME, 0.54. Ocular Quadrate, 0.58 long, 1.28 wide. MOQ: length, 0.5; front width, 0.46; back width, 0.86. Clypeus narrow and eye tubercle distinct.

Maxilla (Fig. 10B): prolateral face smooth with a few short black setae, retro-face reddish yellow, smooth, glabrous. Front length 2.86, back length 3.98 and 2.3 wide. Cuspules ca. 118 in anterior corner in roughly triangular region.

Labium (Fig. 10B): 1.08 long and 1.76 wide with ca. 30 cuspules in band for ¼ of anterior length; labiosternal groove concave, collar like rise on sternum on lateral sides of groove.

Chelicerae (Fig. 11A–B): prolateral teeth 21 and 45–50 basosomal teeth. A row of spiniform bristles on prolateral cheliceral border functioning as rastellum.

Sternum (Fig. 10B): longer than wide, high in center, slopping gradually, covered with short black setae. Posterior edge pointed but not separating coxa IV. Long and short bristles radiate from margin. Pedicel not clearly seen.

Sigilla (Fig. 10B): three pairs, posterior 0.18 diameter, 1.58 apart; 0.24 distance from margin; middle 0.12 diameter, 2.98 apart, 0.08 distance from margin; anterior 0.08 diameter, 2.86 apart, 0.04 distance from margin.

Legs: formula 4123, prograde; morphometry (femur, patella, tibia, metatarsus, tarsus, total): I: 7.78, 5.9, 4.8, 2.96, 2.16, 23.6. II: 6.72, 4.82, 3.72, 3.04, 2.18, 20.48. III: 6.04, 4.22, 3.4, 4.3, 2.42, 20.38. IV: 6.76, 4.74, 4.5, 6.74, 2.3, 25.04. Palp: 5.04, 3.8, 3.36, –, 2.94, 15.14. Midwidths: femora I – IV = 2.92, 2.58, 1.42, 1.84, palp = 1.4; tibiae I–IV = 2.16, 1.26, 2.16, 1.42, palp = 1.5.

Spines: Leg I - mt v1; Leg II - mt v 2; Leg III - mt p 2, r 2, v 5, ti p 2, r 1, pa p 1; leg IV - mt p 2, r 2, v 5, ti p 1, r 2. Absent elsewhere.

Leg coxae (Fig. 10B, 12A, 13A–I): prolateral coxa of all legs with numerous horizontally aligned pilose setae above coxal suture (Fig. 13A–D); bellow with vertically aligned pyriform setae (Fig. 13A, F–I). Retrolateral face with long black bristles on dorsal retrolateral border. Coxal bases dorsally easily seen from above. Coxae, I–II sloping forward, III–IV sloping backward. Coxa IV widest, I and II almost equal, III thinnest.

Scopulae: entire on tarsi I & II undivided, tarsi III divided by a band of 4–5 rows of short spike setae, tarsi IV divided by a band of 7–8 rows of short spike setae. Metatarsi I–IV undivided and ¼ scopulate.

Trichobothria: tarsi I 18–20 clavate and 8–9 long and short filiform; tarsi II 30–31 clavate and 15–16 long and short filiform; tarsi III 26–27 clavate and 13–14 long and short filiform; tarsi IV 23–24 clavate and 10–11 long and short filiform; palp tarsi with 17–18 clavate. Trichobothria in two rows throughout tarsi. Metatarsi I with 3–4 long and short filiform, metatarsi II–IV with 6–7 long and short filiform; tibia with 2–4 long and short filiform.

Abdomen (Fig. 10A & B): cuticle not exposed dorsally and ventrally; covered with fine layer of dark brown setae intermixed with long silver and black setae.

Spermathecae (Fig. 12B): twin seminal receptacles, each with a rounded globular apex.

Natural history and distribution: A single male was found under a boulder during monsoon. Several female were found under boulders along a dry stream bed in a shallow depression without any webbing. Two females were found holding an egg cocoon each under the chelicerae in the month of May (Fig. 9B). The habitat at the type locality is of evergreen type and is contagious with the forests of Bhimashankar Wildlife Sanctuary. Sympatric theraphosid species observed include *Plesiophrictus millardi*.

#### *Heterophrictus aareyensis* Mirza & Sanap, sp. nov

urn:lsid:zoobank.org:act:99774D5D-5E37-4F57-A6DB-166930FD408F.

Fig. 15–21, 45C.

**Type specimens: INDIA–**holotype, male, 11.viii.2010, elevation 100 m, Aarey Milk Colony, Mumbai, Maharashtra, coll. Rajesh Sanap & Zeeshan Mirza, ZSI/WRC/AR/420. Paratype female, 12.vi.2013, elevation 100 m, Aarey Milk Colony, Mumbai, Maharashtra, coll. Zeeshan Mirza & Rajesh Sanap, BNHS-SP85.

**Diagnosis:** *Heterophrictus aareyensis* **sp. nov.** differs from *H. raveni* **sp. nov.** by being smaller in total length, by possessing stridulatory spiniform setae on the coxae of leg I–II only, in fewer basosomal teeth and by having posterior border of PLE & PME on the same plane (Fig. 39, 105). Females differ from *H. raveni* **sp. nov.** in having the spermathecal stalk wider in diameter and in be short (Fig. 21D).

**Etymology:** The new species is named after Aarey where the type specimens was collected and also to seek attention of locals for conservation of the type locality.

**Description holotype male ZSI/WRC/AR/420 (Fig. 37):** total length 14.28, carapace 6.94 long, 6.52 wide, chelicerae 4.16 long after dissection. Sternum 3.26 long, 3.12 wide. Abdomen 7.34 long, 4.6 wide. Spinnerets: PMS, 0.74 long, 0.2 wide, 0.3 apart; PLS, 1.2 basal, 0.9 middle, 1.06 distal, total length 3.16; midwidths 0.48, 0.44, 0.3 respectively, apart 0.56.

Carapace (Fig. 16A): length to width 1.22; uniform reddish–brown, covered with short brown setae, more concentrated towards margins and along interstitial ridges radiating from fovea. Fovea slightly procurved, slightly smaller than ocular width.

Eyes (Fig. 38 & 105): width to length ratio of ocular group 2.6. Eye diameter: ALE, 0.3; AME, 0.28; PLE, 0.1; PME, 0.12. Distance between eyes: AME–AME, 0.18; PME–PLE, 0.06; AME–ALE, 0.08; PME–PME, 0.56. Ocular Quadrate, 0.5 long, 1.3 wide. MOQ: length, 0.28; front width, 0.48; back width, 0.68. Clypeus narrow and eye tubercle distinct.

Maxilla (Fig. 16B): posterior ventral edge gently rounded and long; retro–face yellowish–red, smooth, glabrous. Front length 2.1, back length 2.38 and 1.2 wide. Cuspules ca 150 in anterior corner in roughly triangular region. Posterior edge concave and heel distinctly pronounced.

Labium (Fig. 16B): over ca. 30 cuspules in band for ¼ of anterior length; Basal groove shallow, distinct. Labiosternal groove with two distinct mounds.

Chelicerae (Fig. 17A–B): prolateral teeth 16 and 24 basosomal teeth, rastellum absent.

Sternum (Fig. 16B): longer than wide, high in center, slopping gradually, covered with short black setae. Posterior edge pointed but not separating coxa IV. Long and short bristles radiate from margin. Pedicel not clearly seen.

Sigilla (Fig. 16B): three pairs, posterior 0.18 diameter, 1.38 apart; 0.2 diameter from margin; middle 0.12 diameter; 0.08 distance from margin, apart 2.06; anterior 0.06 diameter, 2.08 apart, 0.04 distance from margin.

Legs: formula 4123, prograde; morphometry (femur, patella, tibia, metatarsus, tarsus, total): I: 6.02, 3.04, 4.72, 3.04, 2.3, 19.12. II: 5.68, 2.8, 4.04, 3.1, 2.44, 18.06. III: 4.84, 2.34, 3.3, 3.66, 2.14, 16.28. IV: 6.3, 2.56, 5.18, 6.16, 3.02, 23.22. Palp: 4.14, 2.54, 3.22, –, 1.02, 10.92. Midwidths: femora I–IV = 1.3, 1.42, 1.48, 1.48, palp = 0.98; tibiae I–IV = 1.28, 1.04, 1.18, 0.98, palp = 1.26.

Spines: Leg I - mt v1, ti v 2; Leg II - mt v 3, ti v 3, Leg III - mt p 2, r 1, v 5, ti p 1, r 1, v 2, pa p 1; Leg IV - mt p 2, r 2, v 5, ti v 5, r 2. Absent elsewhere.

Spike setae (Fig. 17C): a cluster of 10–12 spike setae present on basal region of retrolateral aspect of tibia of leg I.

Leg coxae: prolateral coxa of leg I & II with few horizontally aligned pilose setae below coxal suture; above with vertically aligned pyriform setae. Retrolateral face with long black bristles on dorsal retrolateral border. Coxal bases dorsally easily seen from above. Coxae, I–II sloping forward, III–IV sloping backward.

Scopulae: entire on tarsi I–II, tarsi III entire, divided by a band of 2–3 rows of spike setae; tarsi IV divided by a band of short spike setae.

Trichobothria: tarsi I 12 clavate and 13–14 long and short filiform; tarsi II 24 clavate and 13–14 long and short filiform; tarsi III 18–39 clavate and 12–13 long and short filiform; tarsi IV 18–19 clavate and 13–14 long and short filiform; palp tarsi with 22 clavate and 15–16 long and short filiform. Trichobothria in two rows throughout tarsi. Metatarsi I–II with 7–9, metatarsi III–IV with 6–7 long and short filiform. Tibia I–II with 6–8 and tibia III–IV with 4–6 long and short filiform. Palp tarsi with 18–19 clavate.

Abdomen (Fig. 16A & B): cuticle not exposed dorsally and ventrally; covered with fine layer of dark brown setae intermixed with long silver setaes.

Palp bulb (Fig. 18A–C, 19A–C): smooth, lacking keels, pyriform. Embolus filiform, stout at base gradually tapering in to a fine point.

**Description of female paratype BNHS-SP85 (Fig. 18):** total length 26.22, carapace 11.68 long, 8.98 wide, chelicerae 6.96 long after dissection. Sternum 4.4 long, 4.2 wide. Abdomen 14.54 long, 10.3 wide. Spinnerets: PMS, 0.7 long, 0.46 wide, 0.3 apart; PLS, 1.24 basal, 0.86 middle, 1.28 distal, total length 3.38; midwidths 0.8, 0.7, 0.42 respectively, apart 1.02.

Carapace (Fig. 20A): length to width 1.3; uniform reddish brown, covered with short brown setae, more concentrated towards margins and along interstitial ridges radiating from fovea. Fovea equal to ocular width, slightly procurved.

Eyes (Fig. 19 & 104): width to length ratio of ocular group 2.6. Eye diameter: ALE, 0.34; AME, 0.28; PLE, 0.2; PME, 0.22. Distance between eyes: AME–AME, 0.18; PME–PLE, 0.1; AME–ALE, 0.14; PME–PME, 0.54. Ocular Quadrate, 0.58 long, 1.28 wide. MOQ: length, 0.5; front width, 0.46; back width, 0.86. Clypeus narrow and eye tubercle distinct.

Maxilla (Fig. 20B): prolateral face smooth with a few short black setae, retro-face reddish yellow, smooth, glabrous. Front length 2.86, back length 3.98 and 2.3 wide. Cuspules ca. 118 in anterior corner in roughly triangular region.

Labium (Fig. 20B): 1.08 long and 1.76 wide with ca. 30 cuspules in band for ¼ of anterior length; labiosternal groove concave, collar like rise on sternum on lateral sides of groove.

Chelicerae (Fig. 22A–B): prolateral teeth 21 and 45–50 basosomal teeth. A row of spiniform bristles on prolateral cheliceral border functioning as rastellum.

Sternum (Fig. 20B): longer than wide, high in center, slopping gradually, covered with short black setae. Posterior edge pointed but not separating coxa IV. Long and short bristles radiate from margin. Pedicel not clearly seen.

Sigilla (Fig. 20B): three pairs, posterior 0.18 diameter, 1.58 apart; 0.24 distance from margin; middle 0.12 diameter, 2.98 apart, 0.08 distance from margin; anterior 0.08 diameter, 2.86 apart, 0.04 distance from margin.

Legs: formula 4123, prograde; morphometry (femur, patella, tibia, metatarsus, tarsus, total): I: 7.78, 5.9, 4.8, 2.96, 2.16, 23.6. II: 6.72, 4.82, 3.72, 3.04, 2.18, 20.48. III: 6.04, 4.22, 3.4, 4.3, 2.42, 20.38. IV: 6.76, 4.74, 4.5, 6.74, 2.3, 25.04. Palp: 5.04, 3.8, 3.36, –, 2.94, 15.14. Midwidths: femora I – IV = 2.92, 2.58, 1.42, 1.84, palp = 1.4; tibiae I–IV = 2.16, 1.26, 2.16, 1.42, palp = 1.5.

Spines: Leg I - mt v1; Leg II - mt v 2; Leg III - mt p 2, r 2, v 5, ti p 2, r 1, pa p 1; leg IV - mt p 2, r 2, v 5, ti p 1, r 2. Absent elsewhere.

Leg coxae (Fig. 21B): prolateral coxa of all legs with numerous horizontally aligned pilose setae above coxal suture; bellow with vertically aligned pyriform setae (Fig. 31–34). Retrolateral face with long black bristles on dorsal retrolateral border. Coxal bases dorsally easily seen from above. Coxae, I–II sloping forward, III–IV sloping backward. Coxa IV widest, I and II almost equal, III thinnest.

Scopulae: entire on tarsi I & II undivided, tarsi III divided by a band of 4–5 rows of short spike setae, tarsi IV divided by a band of 7–8 rows of short spike setae. Metatarsi I–IV undivided and ¼ scopulate.

Trichobothria: tarsi I 18–20 clavate and 8–9 long and short filiform; tarsi II 30–31 clavate and 15–16 long and short filiform; tarsi III 26–27 clavate and 13–14 long and short filiform; tarsi IV 23–24 clavate and 10–11 long and short filiform; palp tarsi with 17–18 clavate. Trichobothria in two rows throughout tarsi. Metatarsi I with 3–4 long and short filiform, metatarsi II–IV with 6–7 long and short filiform; tibia with 2–4 long and short filiform.

Abdomen (Fig. 20A & B): cuticle not exposed dorsally and ventrally; covered with fine layer of dark brown setae intermixed with long silver and black setae.

Spermathecae (Fig. 21D): twin seminal receptacles, each with a rounded globular apex.

Natural history: The male holotype was found under a boulder close to human settlement in Aarey Milk Colony. The habitat at the collection site is highly degraded and is dominated by deciduous and exotic flora. The region is rich in its biodiversity but numerous threats from various anthropogenic activities are resulting in great loss to the habitat.

#### *Heterophrictus blatteri* Gravely, 1935

*Plesiophrictus blatteri* Gravely, 1935∶76.

*Plesiophrictus sataraensis* Gravely, 1915∶274 (in part).

*Plesiophrictus mahabaleshwari* Tikader, 1977 **new synonymy.**

*Heterophrictus milleti* Siliwal et al. 2012∶3253 (in part).

Fig. 22–24.

**Material examined:** (*Plesiophrictus sataraensis*) paratype female BMNH 16.5.2.15, Helvak, Koyna valley, Satara district, Maharashtra (Fig. 24A–C); male BNHS SP- 86, 17.vi.2012, Chalakewadi, Satara District, Maharashtra (17°34′40″ N, 73°49′28″ E), collected by Zeeshan Mirza, Nilesh Mane, Ashish Jadhav & Vishal Deshpande. Images of female *Plesiophrictus blatteri* (Type ZSI 1491/18), female *Plesiophrictus sataraensis* (Type ZSI 2207/17), *Plesiophrictus mahabaleshwari* (holotype, no registration number) depicted by Siliwal et al. [14] and Siliwal et al. [5].

**Remark:** Gravely (1935) described *Plesiophrictus blatteri* from Satara district based on specimens of both sexes. Based on fresh collection from Satara district and also supported by Gravely’s [8] description, *P. blatteri* males appear to possess cluster of spike setae on tibia of leg I, females possess rastellum on porolateral dorsal edge of the chelicerae and characteristic stridulatory setae between coxa of legs. This leads us to transfer *P. blatteri* to the genus *Heterophrictus*. Gravely [7] described *P. sataraensis* from the same district based on one male and several females. The male specimen BMNH 2205/17 shows all characteristic of the genus *Plesiophrictus* as prescribed by Sanap & Mirza [12] whereas female specimen (BMNH 16.5.2.15) from the type series possess rastellum (Fig. 24A) and possess characters seen in *Heterophrictus blatteri*. Hereby we conclude that only the male specimens of *P. sataraensis* be the representative of the species and the females of the type series be attributed to *H. blatteri*. Tikader [10] described *P. mahabaleshwari* from the same district which Siliwal et al. (2012) synonymized with *H. milleti*. Based on examined material and images depicted by Siliwal et al. [5], [14] we here remove *H. mahabaleshwari* from the synonymy of *H. milleti* place *it* in the synonymy of *H. blatteri* as a junior synonym based on shape of spermathecae and type locality.

***Neoheterophrictus* Siliwal & Raven, 2012.** *Neoheterophrictus* Siliwal & Raven, 2012 in Siliwal et al. 2012∶3234.

*Plesiophrictus* Gravely 1915∶277 (In part *N. bhori*, In part *N. madraspatanus* Gravely, 1935∶77).

**Type species:** *Neoheterophrictus crurofulvus* Siliwal *et al.* 2012.

**Species included:** *N. amboli* **sp. nov.** *N. crurofulvus* Siliwal *et al.* 2012, *N. sahyadri* Siliwal *et al.* 2012, *N. smithi* **sp. now.**, *N. uttarakannada* Siliwal *et al.* 2012, *N. bhori* **new combination**, *N. madraspatanus* Gravely 1935.

**Diagnosis:** *Neoheterophrictus* males and females differ from all other genera known within the Theraphosidae (except for the Eumenophorinae & Theraphosinae) in possessing stridulatory setae between the coxae of legs I–II (Fig. 13). It differs from known genera within the Eumenophorinae in having the tarsi of leg IV divided by a band of short spike setae instead of normal setae (Fig. 35) and possessing pilose as well as pyriform setae on prolateral coxae. Differs from Theraphosinae in lacking urticating setae. Females differ from all other genera known within the Eumenophorinae (except for *Heterophrictus*) in possessing rastellum on the prolateral border of chelicerae. Differs from *Heterophrictus* in possessing tibial spur (Fig. 30–31), scopulae of leg III undivided and in lacking a cluster of spiniform setae on the retrolateral basal region of tibia I.

**Description:** Medium sized spiders 11–20. Carapace ovate, hirsute, with two clear (setae-less) bands on both sides of caput. Caput low. Fovea slightly procurved. Eye group sub-quadrate to wider than long, tubercle well-defined. Clypeus narrow. Chelicerae normal, with 15–18 teeth on promargin of furrow, basomesally 24–35 small teeth. Rastellum in form of small stout spines on prolateral cheliceral border in females (Fig. 33 & 34). Labium wider than long. Labiosternal grove shallow with two distinct mounds at junction. Cuspules 33–45 in sub-apical region of labium. Maxillae longer than wide, slightly setose, prolateral anterior angle distinctly produced, 140–160 cuspules distributed along proximal prolateral angle. Serrula absent. Sternum as long as wide, sigilla small oval, submarginal. Stridulatory bristles above coxal suture with horizontally aligned thick pilose setae. Base of setae with fine lines on the surface eventually turns pilose in nature. Below these are sparsely arranged thin pilose setae. Bellow suture with numerous vertically aligned pyriform setae. Pyriform setae gradually transform into filiform setae ending in a curve. Several short setae with scoop like tips intermixed with pyriform setae. Legs moderately stout, hirsute, spines present except on femora. Paired claws on legs without dentition and claw tufts well-developed. All tarsi with scopulae entire and only of leg IV divided by a band of spike setae (Fig. 35), metatarsi with ¼ scopulate. Abdomen hirsute, without pattern. PMS well-developed but small; PLS moderately long, apical segment digitiform. Males possess tibial apophysis composed of primary segment being thick and curved. Secondary segment of tibial apophysis in the form of a tubercle, with numerous erect spines upon it. Cluster of spiniform setae absent on retrolateral basal region of tibia I. Palpal organ pyriform, with embolus filiform, ending in a scoop with a sharp tip, lacking keels.

***Neoheterophrictus smithi* Mirza, Bhosale & Sanap sp. nov.** urn:lsid:zoobank.org:act:6362F451-EE39-4EF2-9D9A-9CBD97C161A1

Fig. 25–35, 45E & F

**Type specimens: INDIA–** holotype, male, 1.vii.2011, elevation 72 m, 30 km east of Bhatlak on the periphery of Sharavati Wildlife Sanctuary, Shimoga District, Karnataka (13°58′31.23″N, 74°36′15.54″E), coll. Harshal Bhosale, Sameer Hiremath & Vedant Thite, ZSI/WRC/AR/421.

Other material**–** female, 28.vi.2011, coll. Harshal Bhosale, Sameer Hiremath & Vedant Thite, ZSI/WRC/AR/422, same data as holotype.

**Diagnosis:** Males of *Neoheterophrictus smithi* **sp. nov.** differ from *Neoheterophrictus amboli* **sp. nov.** and *N. sahyadri* in possessing a long slender spine at the base of the primary tibial apophysis and in the tip of the primary tibial apophysis ending in a spine (Fig. 30–31). Additionally the primary tibial apophysis curves at apex tapering gradually and lacks the sub-apical swelling present in *N. amboli* **sp. nov.** (Fig. 30–31)**.** *Neoheterophrictus smithi* **sp. nov.** further differs from *N. amboli* **sp. nov**. in bearing many more basosomal teeth on chelicerae and also bearing spine of patella of leg IV. Differs from *N. crurofulvus* in possessing a long slender spine at the base of the primary tibial apophysis (Fig. 30–31). Female differ from those of *N. uttarakannada* in possessing lower cheliceral teeth count and in lacking 3–4 stiff bristles on the prolateral aspect of the chelicerae.

**Etymology:** The species epithet is in honor of Andrew M. Smith for his valuable contribution towards study of theraphosid spiders which enabled us to carry out the present study and his immense help to ZM & RS throughout their project.

**Description of male holotype ZSI/WRC/AR/421 (Fig. 25A):** total length 19.06, carapace 9.26 long, 6.74 wide, chelicerae 5.04 long after dissection. Sternum 3.8 long, 3.24 wide. Abdomen 9.8 long, 5.78 wide. Spinnerets: PMS 0.72 long, 0.24 wide, 0.18 apart; PLS, 0.96 basal, 0.82 middle, 0.98 distal, total length 2.76; midwidths 0.44, 0.4, 0.38, respectively, 0.58 apart.

Carapace (Fig. 26A): length to with ration.1.37; covered with a short wavy grey setae intermixed with short sparse black setae; setae more concentrated along interstitial ridges radiating from fovea. Short black setae radiating from carapace margin. Fovea slightly procurved.

Eyes (Fig. 45E): width to length ratio of ocular group 1.33; ALE clearly larger than rest, AME slightly smaller than ALE, and PME clearly smaller than rest; anterior line of eyes procurved. Eyes on ocular tubercle. Eye diameter: ALE, 0.24; AME, 0.22; PLE, 0.16; PME 0.14. Distance between eyes: AME–AME, 0.12; PME–PLE, 0.1; AME–ALE, 0.14; PME–PME, 0.76: ALE–PLE, 0.12: ALE–ALE, 0.5; PLE–PLE, 0.98; Ocular Quadrate: 0.6 long, 0.8 wide. MOQ: Length, 0.46; front width, 0.54; back width, 0.8. Clypeus narrow.

Maxillae (Fig. 26B): 2.16 long in front and 2.34 in back, 1.28 wide; more than 156 cuspules arranged in an anterior corner in triangle region. Prolateral face with a few scattered short setaes below and above maxillary suture; retrolateral face smooth and glabrous, with horizontally aligned long bristles in basal region which act against numerous horizontally aligned spike setae on coxa I.

Labium (Fig. 26B): 0.74 long, 0.78 wide. ca. 33 cuspules in band for ¼ of length anteriorly. Basal groove shallow and distinct, labiosternal groove concave with two distinct sigilla.

Chelicerae (Fig. 27A–B): 16 promarginal teeth and 35 basosomal teeth, rastellum absent.

Sternum (Fig. 26B): longer than wide, high in center, slopping gradually, covered with short black setae. Posterior edge pointed but not separating coxa IV. Long and short bristles radiate from margin. Pedicel not clearly seen.

Sigilla (Fig. 26B): three pairs, posterior oval, 0.22 diameter, 1.3 apart, distance from margin 0.32; middle, oval, 0.14 diameter, 2.44 apart, distance form margin 0.18; and anterior, 0.08 diameter, 2.4 apart, distance from marginal 0.06.

Legs: formula 4123, prograde; morphometry (femur, patella, tibia, metatarsus, tarsus, total): I: 7.40, 4.02, 6.18, 4.82, 3.04, 25.46. II: 6.22, 3.56, 5.04, 4.6, 2.9, 22.32. III: 6.24, 2.68, 4.42, 5.28, 3.06, 21.68. IV: 8.88, 3.44, 7.26, 8.56, 3.46, 31.6. Palp: 4.3, 2.6, 3.4, –, 1.2, 11.5. Midwidths: femora I – IV = 2.54, 2.4, 2.32, 2.58, palp = 1.14; tibiae I–IV = 1.3, 1.24, 1.3, 1.58, palp = 1.24.

Spines: leg I - mt v1; Leg II - mt v 3, ti p 1, v 3; Leg III - mt p 2, r 2, v 6, ti p 1, r 1, v 4; Leg IV - mt p 3, r 2, v 8, ti p 1, v 5, r 3; pa p 1. Absent elsewhere.

Leg coxae: prolateral coxa of all legs with numerous horizontally aligned pilose setae below coxal suture; above with vertically aligned pyriform setae. Retrolateral face with long black bristles on dorsal retrolateral border. Coxal bases dorsally easily seen from above. Coxae, I–II sloping forward, III–IV sloping backward, ventrally with short.

Scopulae: entire on tarsi I & II undivided, tarsi III divided by a band of 2–3 rows of short spike setae, tarsi IV divided by a band of 4–5 rows of short spike setae. Metatarsi I–IV undivided and ¼ scopulate.

Trichobothria: tarsi I – IV 25–26 clavate and 10–16 long and short filiform; palp tarsi with 11–12 clavate. Trichobothria in two rows throughout tarsi. Metatarsi I & II 10–12 long and short filiform, metatarsi III 14–15 long and short filiform, Metatarsi IV 7–8 long and short filiform; tibia I 13–14, tibia II–IV & palp tibia 5–9 long and short filiform, in two mid-dorsal rows.

Abdomen (Fig. 26B): covered with a mat of short brown setae mixed with long black as well as gray setae. Dorsal region near the pedicel with numerous black bristles. Ventrally with short brown setae and long gray ones.

Tibial apophysis (Fig. 30–31): primary segment thick, curved at apex, tapers gradually into a spine. Primary segment reddish brown at base gradually turning black towards the distal portion. Secondary segment in tubercle form, bearing upon it numerous erect spines and a stout curved spine arises from its base which terminates in a blunt tip.

Palp Bulb (Fig. 28–29): embolus filiform, broader at base gradually tapering into a fine point, which curves upwards.

**Description of immature female ZSI/WRC/AR/422 (Fig. 25B):** total length 11.36, carapace 6.8 long, 5.68 wide, chelicerae 4.94 long. Sternum 2.8 long, 2.86 wide. Abdomen 4.56 long, 9.04 wide. Spinnerets: PMS 1.74 Long, 1.16 wide, 0.14 apart; PLS, 1.2 basal, 0.7 middle, 0.98 distal, total length 2.88; midwidths 0.5, 0.44, 0.36, respectively, 1.56 apart.

Carapace (Fig. 32A): length to with ration.1.19; covered with a short wavy grey setae intermixed with short sparse black setae; setae more concentrated along interstitial ridges radiating from fovea. Short black setae radiating from carapacial margin. Fovea slightly procurved.

Eyes (Fig. 45F): width to length ratio of ocular group 2.04; AME clearly larger than rest, anterior line of eyes procurved. Eyes on ocular tubercle. Eye diameter: ALE, 0.18; AME, 0.2; PLE, 0.16. Distance between eyes: AME–AME, 0.1; AME–ALE, 0.12; ALE–PLE, 0.08; ALE–ALE, 0.54; PLE–PLE, 0.7; Ocular quadrate: 0.48 long, 0.98 wide; MOQ: Length, 0.32; front width, 0.46; back width, 0.58. Clypeus absent. PME absent, likely a deformity.

Maxillae (Fig. 32B): 2.12 long in front and 2.6 in back, 1.4 wide; more than 145 cuspules arranged in an anterior corner in triangle region. Prolateral face with a few scattered short setaes below and above maxillary suture; retrolateral face smooth and glabrous, with horizontally aligned long bristles in basal region which act against numerous horizontally aligned spike setae on coxa I.

Labium (Fig. 32B): 0.7 long, 0.9 wide. ca. 45 cuspules in band for ¼ of length anteriorly. Basal groove shallow and distinct, labiosternal groove concave with two distinct sigilla.

Chelicerae (Fig. 33–34): 18 promarginal teeth and 30 basosomal teeth; short spine present on cheliceral prolateral border in form of rastellum.

Sternum (Fig. 32B): 2.8 long, 2.86 wide; oval, high in center, sloping gradually, reddish, covering with short black setae. Long and short setae radiating from margin.

Sigilla (Fig. 32B): three pairs, posterior oval, 0.26 diameter, 1.22 apart, distance from margin 0.3; middle, oval, 0.18 diameter, 2.04 apart, distance form margin 0.14; anterior, 0.1 diameter, 2 apart, distance from margin 0.06.

Legs: formula 4123, prograde; morphometry (femur, patella, tibia, metatarsus, tarsus, total): I: 9.8, 4.66, 8.56, 5.66, 3.62, 32.3. II: 8.02, 2.92, 5.96, 5.44, 3.54, 25.88. III: 8.26, 3.18, 4.82, 6.22, 4.1, 26.58. IV: 10.74, 3.78, 7.42, 9.9, 4.22, 36.06. Palp: 5.62, 3.38, 4.8, –, 2.06, 15.86. Midwidths: femora (I, II, III, IV, Palp) 2.6, 2.04, 2.04, 2.2, 8.88; tibia (I–IV, Palp) 1.52, 1.48, 1.3, 1.4, 5.7.

Spines: leg I - mt v1, ta v 1; Leg II - mt v 4, ti v 1; Leg III - mt p 2, r 2, v 6, ti p 1, r 1, v 3; Leg IV - mt p 2, r 2, v 11, ti p 1, v 3, r 3. Absent elsewhere.

Leg coxae: prolateral coxa of all legs with numerous horizontally aligned pilose setae below coxal suture; above with vertically aligned pyriform setae. Retrolateral face with long black bristles on dorsal retrolateral border. Coxal bases dorsally seen from above. Coxae, I–II sloping forward, III–IV sloping backward, ventrally with short.

Scopulae (Fig. 35): entire on tarsi I divided by 2–3 rows of short spike setae, tarsi II divided by 4–5 rows of short spike setae, tarsi III & IV divided by a band of 3–4 rows of short spike setae. Metatarsi I–II 2/3 scopulate and III–IV ¼ scopulate and undivided.

Trichobothria: tarsi I, 16–18 clavate and 8–9 long and short filiform in distal half in two rows; tarsi II, 20–21 clavate and 8–9 long and short filiform in distal half in two rows; tarsi III, 9–10 clavate and 6–7 long and short filiform in distal half in two rows; tarsi IV, 2 clavate and 8–9 long and short filiform in distal half in two rows; palp tarsi with 22–23 clavate and 7–8 long and short filiform in distal half in two rows.

Abdomen (Fig. 32): covered with a mat of short brown setae mixed with long gray setae lost after preservation rendering the cuticle entirely exposed dorsally.

Natural history and distribution: A single male specimen was found along a road under an *Albizia saman* tree in the night. The forest type in the area of evergreen type and revives heavy rainfall. The paratype female was found under a boulder in a shallow depression without any webbing. The female tried to escape as the rock was turned. Several more immature female specimens were encountered in the same area in a similar depression under boulders. This species is presently known only from the type locality which lies on the outskirts of Sharavati Wildlife Sanctuary and it is likely that this species will also be found there in addition to the type locality as the areas shares a similar biotope.

***Neoheterophrictus amboli* Mirza & Sanap sp. nov:** urn:lsid:zoobank.org:act:A63A33C5-4310-4F89-9367-186FCF3F61AA

Fig. 36–42, 45D

**Type material:** holotype, male, 20.vi.2009, elevation 702 m, Amboli Ghat, Sindhudurg District, Southern Maharashtra (15°57′47.88″N, 74° 0′10.85″E), collected by Sushant Gavas, ZSI/WRC/AR/423.

**Diagnosis:** *Neoheterophrictus amboli* **sp. nov.** differs from *N. smithi* **sp. nov.** in possessing a distinct sub-apical swelling on the primary segment of tibial apophysis which abruptly terminates into a blunt tubercle. Further differs from *N. smithi* **sp. nov.** in possessing fewer cheliceral basosomal teeth. Differs from *N. crurofulvus* in possessing a long slender spine at the base of the primary tibial apophysis. Differs from *N. sahyadri* in possessing a stout and short spine on the secondary tibial apophysis (spine slightly longer and slender in *N. sahyadr*) (Fig. 41–42).

**Etymology:** Named after Amboli, where the holotype was collected. Amboli is treated as an invariable noun in apposition to the generic name.

**Description of male holotype ZSI/WRC/AR/423 (Fig. 36):** total length 20.2, carapace 9.78 long, 8.4 wide, chelicerae 5.6 long. Sternum 4.8 long, 3.46 wide. Abdomen 10.42 long, 4.82 wide. Spinnerets: PMS, 0.6 Long, 0.2 wide, 0.32 apart; PLS, 1.12 basal, 0.78 middle, 0.98 distal, total length 2.88; midwidths 0.5, 0.38, 0.36, respectively, 0.98 apart.

Carapace (Fig. 37A): reddish brown covered overall with gray wavy setae. Fovea slightly procurved. Length to with ratio 1.34.

Eyes (Fig. 45D): ratio of group width to length 2.27; ALE clearly larger than rest, AME slightly larger than PLE, and PME clearly smaller than rest. Eyes on ocular tubercle. Eye diameter: ALE, 0.32; AME, 0.3; PLE, 0.28; PME 0.2. Distance between eyes: AME–AME, 0.28; PME–PLE, 0.1; AME–ALE, 0.08; PME–PME, 0.68. Ocular Quadrate: 0.74 long, 1.32 wide. MOQ: Length, 0.42; front width, 0.62; back width, 0.9. Clypeus absent and eye tubercle distinct.

Maxillae (Fig. 37B, 38C): posterior ventral edge gently rounded; maxillae 2.78 long in front and 4.16 in back, 1.80 wide; ca. more than 140 cuspules sparsely arranged in an anterior corner in triangle region. Prolateral face with a few scattered short setaes below and above maxillary suture; retrolateral face smooth and glabrous.

Labium (Fig. 37B): 1.04 long, 1.16 wide. ca. 37 cuspules in band for ¼ of length anteriorly. Basal groove shallow and distinct. Labiosternal groove concave with two distinct sigillae.

Chelicerae (Fig. 38A–B): 15 promarginal teeth and 24–25 basosomal teeth; Chelicerae rastellum absent.

Sternum (Fig. 37B): 4.8 long, 3.46 wide; oval, high in center, sloping gradually, reddis h, covering with short black setae. Long and short setae radiating from margin.

Sigilla: three pairs, posterior oval, 0.32 diameter, 1.6 apart, distance from margin 0.48; middle, oval, 0.24 diameter, 2.34 apart, distance form margin 0.3; and anterior sigilla marginal.

Legs: formula 4123, prograde; morphometry (femur, patella, tibia, metatarsus, tarsus, total): I: 9.8, 4.66, 8.56, 5.66, 3.62, 32.3. II: 8.26, 3.18, 4.82, 6.22, 4.1, 26.58. III: 8.02, 2.92, 5.96, 5.44, 3.54, 25.88. IV: 10.74, 3.78, 7.42, 9.9, 4.22, 36.06. Palp: 5.62, 3.38, 4.8, –, 2.06, 15.86. Midwidths: femora (I, II, III, IV, Palp) 2.6, 2.04, 2.04, 2.2, 8.88; tibia (I, II, III, IV, Palp) 1.52, 1.48, 1.3, 1.4, 5.7.

Spines: leg I - mt v2; Leg II - mt p 1, v 5, d 4, ti v 3; leg III - mt r 2, v 7, ti p 1, r 1, v 3; leg IV - mt p 3, r 2, v 7+1 (broken), ti v 3. Absent elsewhere.

Leg coxae: prolateral coxa of leg I & II with few horizontally aligned pilose setae below coxal suture; above with vertically aligned pyriform setae. Retrolateral face with long black bristles on dorsal retrolateral border. Coxal bases dorsally easily seen from above. Coxae, I–II sloping forward, III–IV sloping backward, ventrally with short.

Scopulae: tarsi I–III, entire and undivided; tarsi IV, entire, divided centrally with 3–4 row of spike setae.

Trichobothria: tarsi I, 11–12 clavate and 9–10 long and short filiform in distal half in two rows; tarsi II, 13–14 clavate and 11–12 long and short filiform in distal half in two rows; tarsi III, 16–17 clavate and 12–13 long and short filiform in distal half in two rows; tarsi IV, 14–15 clavate and 12–13 long and short filiform in distal half in two rows; palp tarsi with 22–23 clavate and 10–11 long and short filiform in distal half in two rows. Metatarsi I–II with 4–9, metatarsi III–IV with 9–10 long and short filiform. Tibia I–II with 4–6 and tibia III–IV with 5–8 long and short filiform. Palp tarsi with 20–21 clavate.

Abdomen (Fig. 37): covered with a mat of short brown setae with numerous long gray setaes. Similar on the venter, however of the gray setae are sparse in distribution.

Tibial apophysis (Fig. 41–42): primary segment black thick, curved at apex and with a prominent bulge at sub apical region further transforming abruptly into a blunt tubercle. Secondary segment in tubercle form bearing numerous erect spines upon it and a stout curved spine arises from its base which terminates in a blunt tip.

Palp bulb (Fig. 39–40): embolus filiform, broader at base gradually tapering into a fine point, which curves upwards.

***Neoheterophrictus bhori* Gravely, 1915 new combination.** *Plesiophrictus bhori* Gravely, 1915∶277

*Heterophrictus bhori*; Siliwal et al. 2012∶3253

Fig. 43–44

**Material examined:** syntype female, BMNH 16.5.2.16, Parambikulam, Kerala, India. Collected by F. H. Gravely.

**Remarks:** Presence of rastellum on the chelicerae (Fig. 44A), horizontally aligned stridulatory setae above maxillary suture (Fig. 44B) and multi-lobed spermathecae (Fig. 44C) suggests placement of this taxa in the genus *Neoheterophrictus*. The type series contains specimens from several localities which represent more than one species [14] which also appears to be a case with several species of this subfamily from India.

***Sahydroaraneus* gen. nov. Mirza & Sanap.** *Annandaliella travancorica* Gravely 1915∶271 (in part)

urn:lsid:zoobank.org:act:4BAE0A29-6167-41FF-8FBC-2AC6E95BCAAD

**Type species:** *Sahydroraraneus hirsti* **sp. nov.**

**Species included:** *Sahydroraraneus hirsti* **sp. nov.,** *S. collinus* Pocock, 1899 **new combination**, *Sahydroaraneus raja* Gravely 1915 **new combination.**

**Figure 46:**
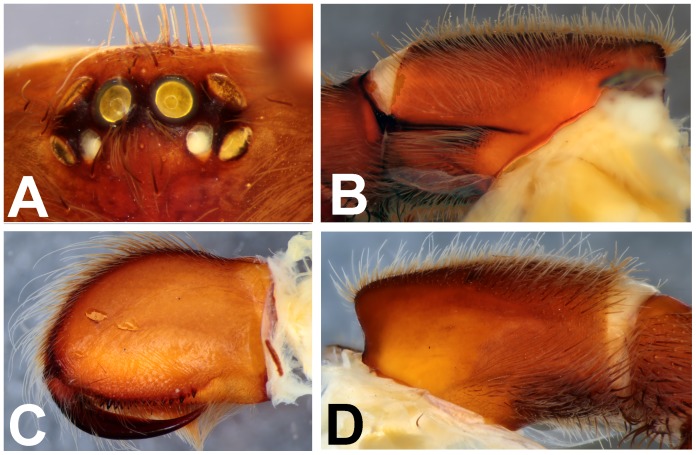
*Sahydroaraneus hirsti* sp. nov. male holotype BMNH 16.5.2.12. A. eye, B. coxa leg I prolateral view, C. chelicerae prolateral view, D. coxa leg I retrolateral view.

**Diagnosis:** ***Sahydroaraneus*** **gen. nov.** males differ from all other genera known within the Theraphosidae (except for the Eumenophorinae & Theraphosinae) in possessing stridulatory setae between the coxae of legs I–II (Fig. 13). It differs from known genera within the Eumenophorinae in having the tarsi of leg IV divided by a band of short spike setae instead of normal setae (Fig. 35) and possessing pilose as well as pyriform setae on prolateral coxae. Differs from Theraphosinae in lacking urticating setae. Differs from *Heterophrictus* in possessing tibial spur (Fig. 48A–C), scopulae of leg III undivided and in lacking a cluster of spiniform setae on the retrolateral basal region of tibia I. Males of ***Sahydroaraneus*** **gen. nov.** closely resemble *Neoheterophrictus* but can be differentiated based on absence of secondary tibial apophysis and in the primary apophysis comprises of a basal stalk which gives rise to a thick long black spine terminating in a sharp tip (Fig. 48A–C). ***Sahydroaraneus*** **gen. nov.** males further differ from those of *Heterophrictus* and *Neoheterophrictus* in possessing thick black setae on the pro-dorsal edge of chelicerae whereas these setae are short thinner in the latter two (Fig. 46C). ***Sahydroaraneus*** **gen. nov.** females possess stouter and longer rastellum tooth as opposed to short small ones seen in other two Indian eumenophorine genera (Fig. 51A).

**Figure 47:**
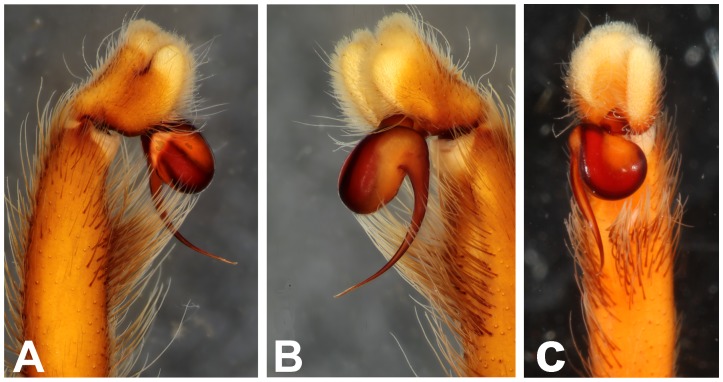
*Sahydroaraneus hirsti* sp. nov. male holotype BMNH 16.5.2.12. Palp bulb, A. retrolateral view; B. prolateral view; C. dorsal view.

**Figure 48:**
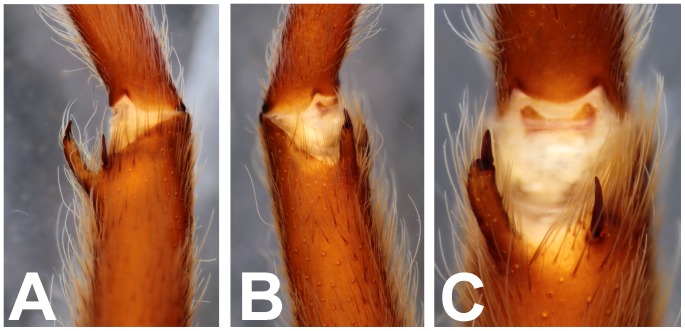
*Sahydroaraneus hirsti* sp. nov. male holotype BMNH 16.5.2.12. Tibial apophysis, A. retrolateral view; B. prolateral view; C. dorsal view.

**Figure 49:**
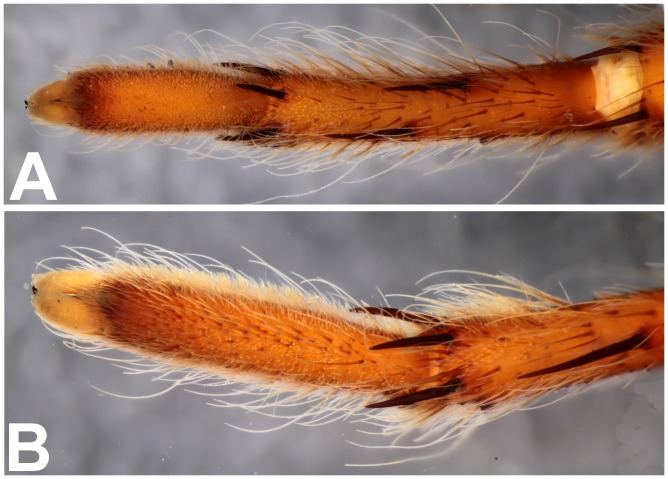
*Sahydroaraneus hirsti* sp. nov. male holotype BMNH 16.5.2.12. Leg scopulae, A. leg III, ventral view; B. leg IV, ventral view.

**Figure 50:**
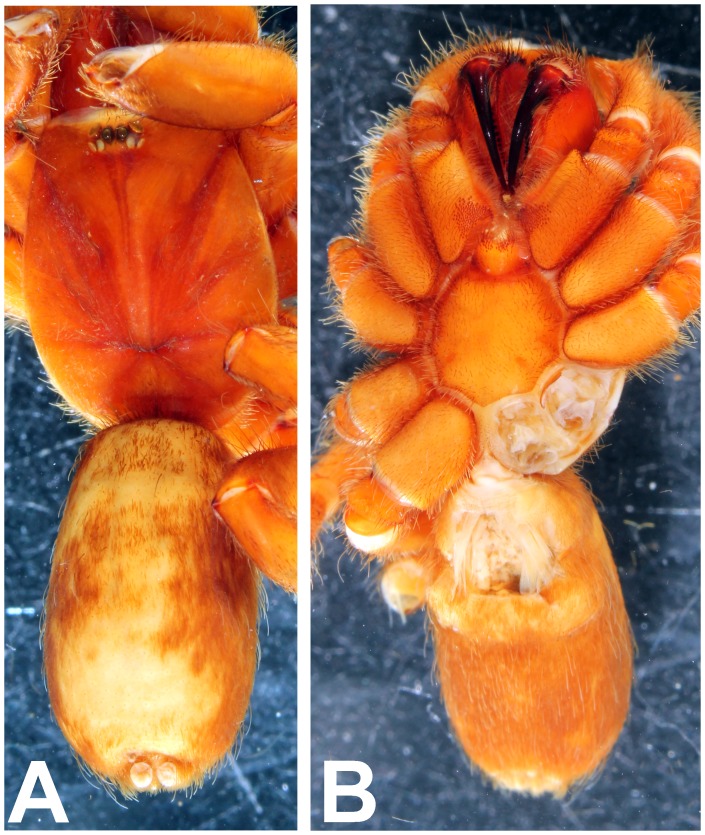
*Sahydroaraneus raja* female type BMNH 16.5.2.17. A. Cephalothorax and abdomen, dorsal view; B. Sternum, labium, maxillae, abdomen and chelicerae, ventral view.

**Figure 51:**
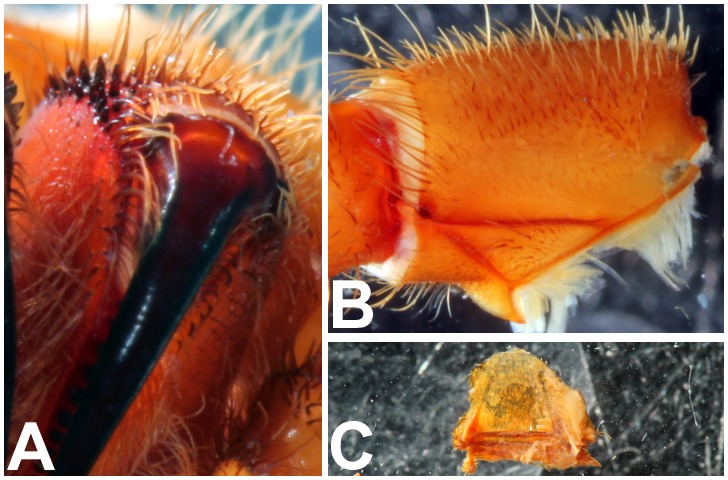
*Sahydroaraneus raja* female type BMNH 16.5.2.17. A. chelicerae pro-dorsal view, coxa leg I, proalteral view; C. absence of spermathecae indicating an immature specimen.

**Etymology:** The proposed generic name comprises of two words “*Sahydro*” referring to the Sahydri hills which is synonymous to the Western Ghats within which the type locality is located and the second word “*araneus*” refers to a spider in Latin. The generic name is treaded as masculine in gender.

**Description:** Small sized spiders 14.61. Carapace ovate, hirsute, with two clear (setae-less) bands on both sides of caput. Caput low. Fovea slightly procurved. Eye group sub-quadrate to wider than long, tubercle well-defined. Clypeus narrow. Chelicerae normal, with 13 teeth on promargin of furrow, rastellum presein in females on the pro-dorsal edge. Labium wider than long. Labiosternal grove shallow with two distinct mounds at junction. Cuspules 33–45 in sub-apical region of labium. Maxillae longer than wide, slightly setose, prolateral anterior angle distinctly produced, 140–160 cuspules distributed along proximal prolateral angle. Serrula absent. Sternum as long as wide, sigilla small oval, submarginal. Stridulatory bristles above coxal suture with horizontally aligned thick pilose setae. Base of setae with fine lines on the surface eventually turns pilose in nature. Pyriform setae absent. Legs moderately stout, hirsute, spines present except on femora. Paired claws on legs without dentition and claw tufts well-developed. All tarsi with scopulae entire and only of leg IV divided by a band of spike setae (Fig. 74& 79), metatarsi with ¼ scopulate. Abdomen hirsute, without pattern. PMS well-developed but small; PLS moderately long, apical segment digitiform. Males possess tibial apophysis composed of primary segment long, curved, of the same pigment as tibia, at its distal end gives rise to a long, thick black spine which ends in a sharp tip. Distinct collar like structure present at the base of the long spine. Secondary segment absent. A long spine present on the ventral aspect of the tibia close to the tibial apophysis. Cluster of spiniform setae absent on retrolateral basal region of tibia I. Palpal organ pyriform, with embolus filiform, ending in a scoop with a sharp tip, lacking keels. Spermathecae with single lobe on each stalk; stalk broader at base and tapers towards its distal end which gives rise to a single bud like lobe.

***Sahydroaraneuss hirsti* sp. nov. Mirza & Sanap.** *Annandaliella travancorica* Gravely 1915∶271 (in part)

urn:lsid:zoobank.org:act:69498168-ABB5-469A-BF03-6B2CE5DC564A

Figs. 46–49

**Type material:** holotype, male, Trichur, Cochin state now Trissur district in Kerala state, collected by F. H. Gravely, BMNH 16.5.2.12.

**Diagnosis:** A medium sized species in relation to members of this subfamily (14.61 mm); with ALE being the largest, posterior border of eyes on the same plane (Fig. 46A). Chelicerae with 13 promarginal teeth. Primary segment of tibial apophysis long, curved, of the same pigment as tibia, at its distal end gives rise to a long, thick black spine which ends in a sharp tip. Distinct collar like structure present at the base of the long spine. Secondary segment absent. A long spine present on the ventral aspect of the tibia close to the tibial apophysis (Fig. 48 A–C).

**Etymology:** The new species is named in honor of A. S. Hirst for his valuable contribution towards Arachnology.

**Description of male holotype BMNH 16.5.2.12:** total length 14.61, carapace 7.38 long, 6.49 wide, chelicerae 4.06 long. Sternum 3.03 long, 3.09 wide. Abdomen 7.23 long, 4.85 wide. Spinnerets: PMS, 0.49 Long, 0.28 wide, 0.23 apart; PLS, 0.96 basal, 0.72 middle, 0.98 distal, total length 2.66; midwidths 0.45, 0.39, 0.29, respectively, 0.94 apart.

Carapace: reddish brown covered overall with gray wavy setae. Fovea slightly procurved. Length to with ratio 1.13.

Eyes (Fig. 46A): ratio of group width to length 3; ALE clearly larger than rest, ALE slightly larger than AME, and PME clearly smaller than rest. Eyes on ocular tubercle. Eye diameter: ALE, 0.22; AME, 0.21; PLE, 0.18; PME 0.12. Distance between eyes: AME–AME, 0.12; PME–PLE, 0.09; AME–ALE, 0.08; PME–PME, 0.50. Ocular Quadrate: 0.40 long, 1.20 wide. MOQ: Length, 0.29; front width, 0.36; back width, 0.46. Clypeus absent and eye tubercle distinct.

Maxillae: posterior ventral edge gently rounded; maxillae 2.44 long in front and 1.01 in back, 1.99 wide; ca. more than 120 cuspules sparsely arranged in an anterior corner in triangle region. Prolateral face with a few scattered short setaes below and above maxillary suture; retrolateral face smooth and glabrous.

Labium: 0.79 long, 1.08 wide. ca. 35 cuspules in band for ¼ of length anteriorly. Basal groove shallow and distinct. Labiosternal groove concave with two distinct sigillae.

Chelicerae (Fig. 46C): 13 promarginal teeth. Cheliceraal pro-dorsal edge with thick black setae likely represent rastellum.

Sternum: 3.06 long, 3.09 wide; oval, high in center, sloping gradually, reddish, covering with short black setae. Long and short setae radiating from margin.

Sigilla: three pairs, posterior oval, 0.27 diameter, 1.42 apart, distance from margin 0.15; middle, oval, 0.15 diameter, 2.54 apart, distance form margin 0.09; anterior sigilla oval, 0.9 diameter, 2.75 apart, distance form margin 0.01.

Legs: formula 4123, prograde; morphometry (femur, patella, tibia, metatarsus, tarsus, total): I: 7.27, 3.81, 5.85, 4.76, 2.64, 24.33. II: 5.00, 2.56, 4.65, 3.54, 2.15, 17.9. III: 5.82, 1.14, 3.62, 4.11, 2.21, 16.9. IV: 6.07, 3.42, 5.57, 7.33, 3.32, 25.71. Palp: 4.43, 2.60, 3.70, –, 1.59, 12.32. Midwidths: femora (I, II, III, IV, Palp) 1.72, 1.68, 1.85, 1.67, 1.51; tibia (I, II, III, IV, Palp) 1.20, 1.05, 1.28, 1.09, 1.36.

Spines: leg I - mt v1; Leg II - mt v 4, ti v 3; leg III - mt p 2, r 3, v 6, ti p 2, r 1, v 4, pa p 1; leg IV - mt p 2, r 2, v 6, d 2, ti, p 2, r 2, v 7. Absent elsewhere.

Leg coxae (Fig. 46B & D): prolateral coxa of leg I & II with few horizontally aligned pilose setae above coxal suture; Retrolateral face with long black bristles on dorsal retrolateral border. Coxal bases dorsally easily seen from above. Coxae, I–II sloping forward, III–IV sloping backward.

Scopulae: tarsi I–III, entire and undivided; tarsi IV, entire, divided centrally with 3–4 row of spike setae.

Abdomen (Fig. 49A–B): covered with a mat of short brown setae with numerous long gray setaes. Similar on the venter, however of the gray setae are sparse in distribution.

Tibial apophysis (Fig. 48A–C): primary segment long, curved, of the same pigment as tibia, at its distal end gives rise to a long, thick black spine which ends in a sharp tip. Distinct collar like structure present at the base of the long spine. Secondary segment absent. A long spine present on the ventral aspect of the tibia close to the tibial apophysis.

Palp bulb (Fig. 47A–C): embolus filiform, broader at base gradually tapering into a fine point, which curves upwards.

***Sahydroaraneus raja* Gravely, 1915 new combination.** Plesiophrictus raja Gravely 1915∶276

Figs. 50–51

**Material examined:** holotype female, BMNH 16.5.2.17, Kavalai, Cochin state now in Kerala. Collected by F. H. Gravely. Type locality situated now in Chimmini Wildlife Sancturay landscape in Thrissur district, Kerala at an elevation of 1300–3000 feet.

**Remark:** The type specimen appears to be an immature specimen as spermathecae cannot be seen (Fig. 51C) and stridulatory setae between the coxa of legs is not well developed (51B). But the specimen shows presence of distinctly stout and long rastellar teeth (Fig. 51A) unlike the short rastellar teeth seen in *Heterophrictus* and *Neoheterophrictus*. In addition to this, based on the proximity of the type locality of *Plesiophrictus raja* to that of *Sahyroaraneus hirsti*, we provisionally place it in the genus *Sahydroaraneus* until mature females and males of the species are examined.****

***Sahydroaraneus collinus* Pocock 1899 new combination.** *Plesiophrictus collinus* Pocock 1899∶749

Figs. 52

**Figure 52:**
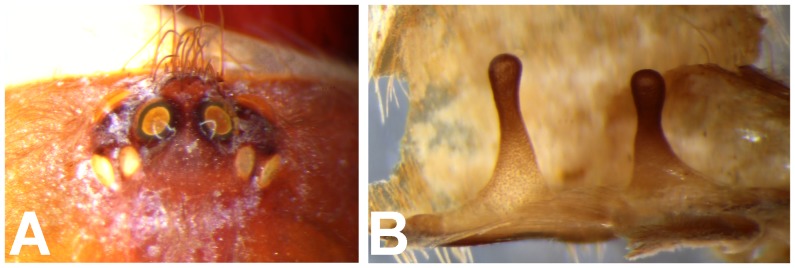
*Sahydroaraneus collinus* female type BMNH 19.16.29. A. eye; B. spermathecae.

**Material examined:** holotype female, BMNH 19.16.29, Yercaud in Shevaroy hills, Tamil Nadu. Collected by J. R. Henderson.

**Remark:** Chelicerae possesses rastellum on its pro-dorsal edge, stridulatory setae present between coxa of legs peculiar to Eumenophorinae. Given that the spermathecae does not possess multiple lobes, it cannot be placed in the genus *Neoheterophrictus*. *Heterophrictus* appears to be restricted to the northern Western Ghats of Maharashtra and hence we provisionally place this species in the newly erected genus *Sahydroaraneus* until males from the type locality are examined.

### Discussion

**Figure 53:**
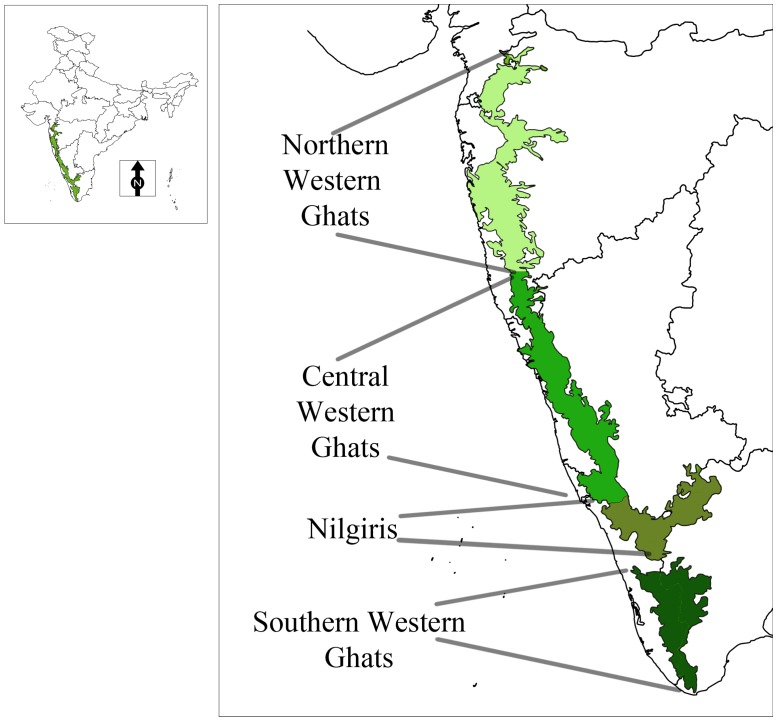
Map showing location of Western Ghats in India and biogeographic zones within Western Ghats based on floral composition [**46**].

Mygalomorph spiders include trapdoor spiders, tarantulas, funnel web spiders and their kin, represented in 15 families that containing 326 genera and 2,780 nominal species including the present work [2], [39]. Being an ancient monophyletic group mygalomorphs, which evolved nearly 300–250 MYA, [43] retain several characteristics that are considered primitive for spiders, e.g., chelicerae orientation, two pairs of book lungs, simple silk-spinning structures, etc. Many mygalomorph taxa are dispersal-limited and regionally endemic, and have long been favorites of biogeographers [39]. Considering the inability of most mygalomorph spider to disperse far distance with the exception of a few demonstrated using molecular data and opportunistic observations [44], [45], they form a vital group for the study of evolutionary history as majority of the species are restricted in distribution. Africa, Madagascar, associated islands and India share many common mygalomorph elements, most of which are presently known only from the Western Ghats in India (Fig. 53) and in Sri Lanka [see 23, 36]. Discovery of five new species of Eumenophorinae adds to growing evidence of past isolation in the biogeographical history of India. Interestingly, India shares several mygalomorph elements with its Mesozoic partner Madagascar and associated islands as opposed to absence of major groups of vertebrates, such as caecilians and representatives of the frog family Nasikabatrachidae, when evolutionary analyses indicate that they should have been there in the past [34]. Presently the following mygalomorph genera are common among Africa, India, Sri Lanka, Madagascar and associated Islands: *Scalidognathus* Karsch, 1891; *Heligmomerus* Simon, 1892; *Idiops* Perty, 1833; *Tigidia* Simon, 1892 [2], [23], [36], [40], [41]. Furthermore several subfamilies like Selenogyrinae and Eumenophorinae are distributed only in Africa and India. It is likely that many more Gondwanan mygalomorph elements will be found in Indian in the long term with detailed studies which will enhance our understanding of the biogeography of this group. Immediate efforts should be made to document other mygalomorph spider in the Western Ghats especially with the help of molecular tools to help evaluate and conserve our evolutionary history.

We hypothesize that *Heterophrictus*, *Neoheterophrictus* and *Sahydroaraneus* **gen. nov.** form a separate clade within eumenophorinae sharing synapomorphy of lacking a brush of stiffened setae on the palp femur which is presumed to be lost in these genera, an uncommon character for the sub-family and in possessing rastellum. However, Indian genera possess stridulatory setae between the coxae of legs, well developed labio-sternal mounds, lacking urticating setae and tibial spur (in *Neoheterophrictus* and *Sahydroaraneus*) supporting their inclusion in the Eumenophorinae. *Heterophrictus* and *Neoheterophrictus* (likely *Sahydroaraneus* as well) possess additional characters like presence of rastellum, pilose and pyriform stridulatory setae between the coxae and lives in shallow depression under boulders without webbing, which make them unique among the Indian theraphosids. Males of *Heterophrictus* possess a cluster of spike setae on the retrolateral basal region of tibia I. These setae have thus far only been reported from this genus and not from any theraphosid genera in the world. The function of these setae at present remains unknown and further studies must be carried out to better understand this structure as it serves as an important taxonomic character.

**Figure 54:**
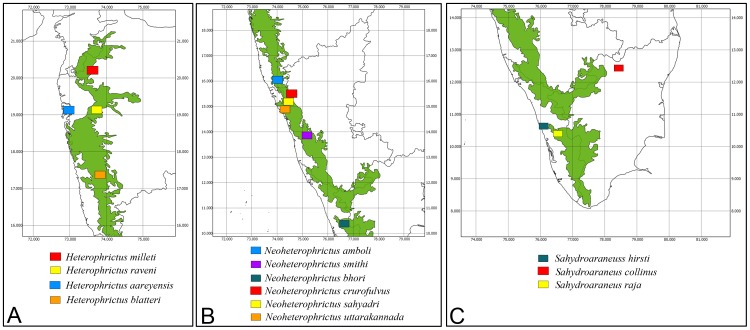
Map showing distribution of Eumenophorinae in India. (A) *Heterophrictus*, (B) *Neoheterophrictus*, (C) *Sahydroaraneus* **gen. nov.**

Pocock [6] in his description of *H*. *milleti* mentions examination of immature specimens of nearly allied species from eastern Pune and Jauli in Satara district in western Mahatashtra. Later, a damaged female specimen from Panchagani, Satara district was identified by Gravely [8] to belong to the genus *Neochilobrachyus* Hirst, 1909. After having examined material from Satara district as well as those examined by Pocock [6], we here conclude that the species from Satara district belong to the genus *Heterophrictus*. Siliwal *et al.* [5] synonymized *Heterophrictus mahabaleshwari* ( = *Plesiophrictus mahabaleshwari* Tikader, 1977 with *H. milleti* based on similar spermathecael shape. We examined several specimens of *Heterophrictus* sp. from Satara district as well as material of *H. milleti* in the collection of NHM and conclude that the species are distinct. However, *H. mahabaleshwari* possesses character seen in females of *H. blatteri* and we here synonymize *H. mahabaleshwari* with *H. blatteri* given that ‘*blatteri’* is the oldest available name. This indicates that the genus is at least widespread in the northern Western Ghats of Maharashtra (Fig. 54A). Sanap & Mirza [12] transferred *Plesiophrictus madraspatanus* Gravely, 1935 to the genus *Neoheteriphrictus* based on morphology of the tibial apophysis which indicates that this genus too is widespread however fresh material from the type locality must be examined to ascertain its generic allocation. *Neoheterophrictus* is presently represented by seven species distributed in the Western Ghats from Southern Maharashtra to Tamil Nadu and one exception of a species from Chennai eastern Tamil Nadu. Gravely [7] described the males of *Annandaliella travancorica* and while doing so also noted that the males lack the characteristic intercheliceral peg setae seen in the genus *Annandaliella*. But he failed to examine presence of stridulatory setae between the coxa of legs. Based on present collection locality data, *Sahydroaraneus* appears to be distributed in Southern Indian states of Kerala and Tamil Nadu. Thus based on the available locality data, *Neoheterophrictus* is restricted to denser, moist evergreen forest in central Western Ghats (Fig. 54B) as opposed to mixed moist deciduous, semi-evergreen forest of Northern Western Ghats of Maharashtra and *Sahydroaraneus* **gen. nov.** is restricted to southern Western Ghats (Fig. 54C). The distribution of these three genera shows a clear pattern based on flora of Western Ghats [46] which in turn is influenced by formation of the Western Ghats.

The recent discovery of the new Eumenophorinae genus *Neoheterophrictus* and *Sahydroaraneus* **gen. nov.** possessing such distinct and unique characters of this Gondwanan sub-family which remained undescribed for so long highlights our lack of knowledge and dearth of work on this group. The present work also brings forth the problems with taxonomic research of Theraphosidae and in general mygalomorph spiders in India [see 11, 12]. Thus dedicated surveys are needed to further enhance our knowledge of these spiders which will consequently enable us to contribute towards their conservation.
