# Correction: Preliminary Review of Indian Eumenophorinae (Araneae: Theraphosidae) with Description of a New Genus and Five New Species from the Western Ghats

**DOI:** 10.1371/journal.pone.0098084

**Published:** 2014-05-16

**Authors:** The PLOS ONE Staff

There are a number of errors in the legends for Figures 1, 2 and 13. The authors have provided corrected versions here.

**Figure 1:**
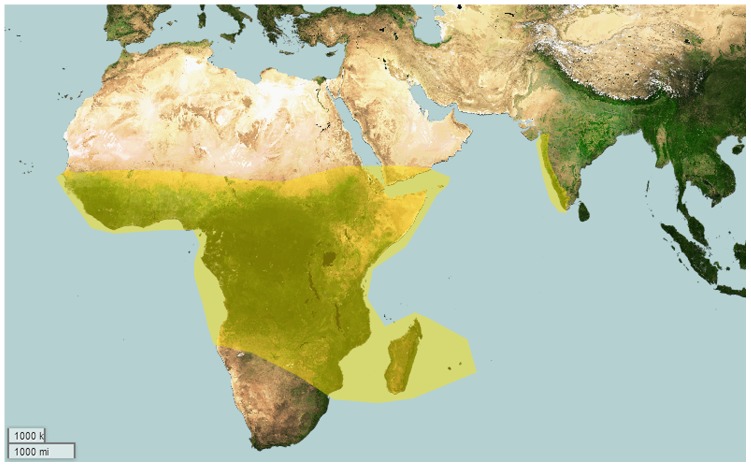
Map depicting global distribution of genera of Eumenophorinae.

**Figure 2:**
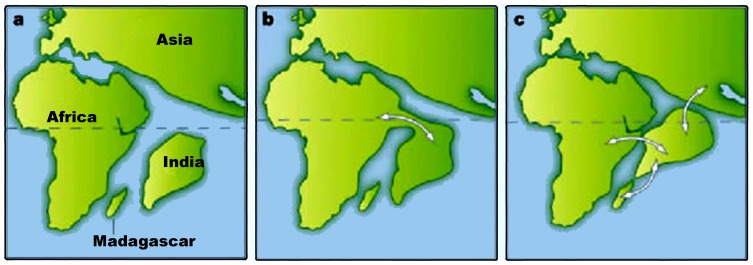
Possible Indian odysseys: different models for the position of India approximately 65 million years ago. a. The standard 'biotic ferry' model showing India isolated by large expanses of water. b. A limited 'biotic (land) bridge' model incorporating a narrow connection (Greater Somalia) with Africa. c. Another biotic bridge model assuming a different longitudinal position for India and showing connections with Madagascar, Africa and Asia (Hedges [42]).

**Figure 13:**
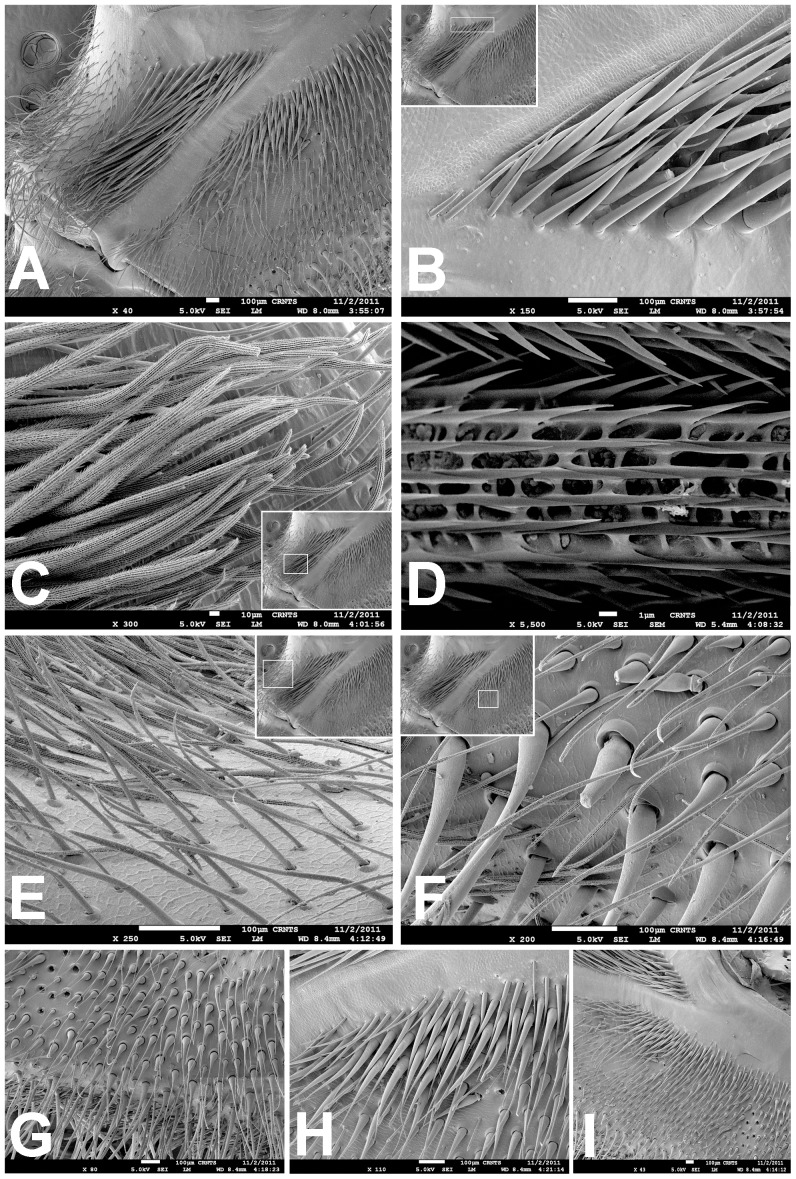
Scanning electron micrograph of *Heterophrictus raveni* **sp. nov**. female paratype (ZSI/WRC/AR/419), coxa II: A. Coxa of leg II prolateral view showing stridulatory setae; B. Basal half of horizontally aligned long pilose setae below coxal suture ; C. Distal half of horizontally aligned long pilose setae; D. Ultra-structure of the surface texture of long pilose setae; D. Short pilose setae in posterior distal region of coxa of leg II; 31. Vertically aligned pyriform setae above coxal suture of leg II; E. Vertically aligned pyriform setae above coxal suture of leg II with curved tips; F. Vertically aligned pyriform setae above coxal suture of leg II basal region; G. Junction of coxal suture of leg II; H-I. prolateral face of coxa showing pyriform setae.

Figure 45 is also incorrectly labeled. A corrected version and the figure legend can be found below.

**Figure 45:**
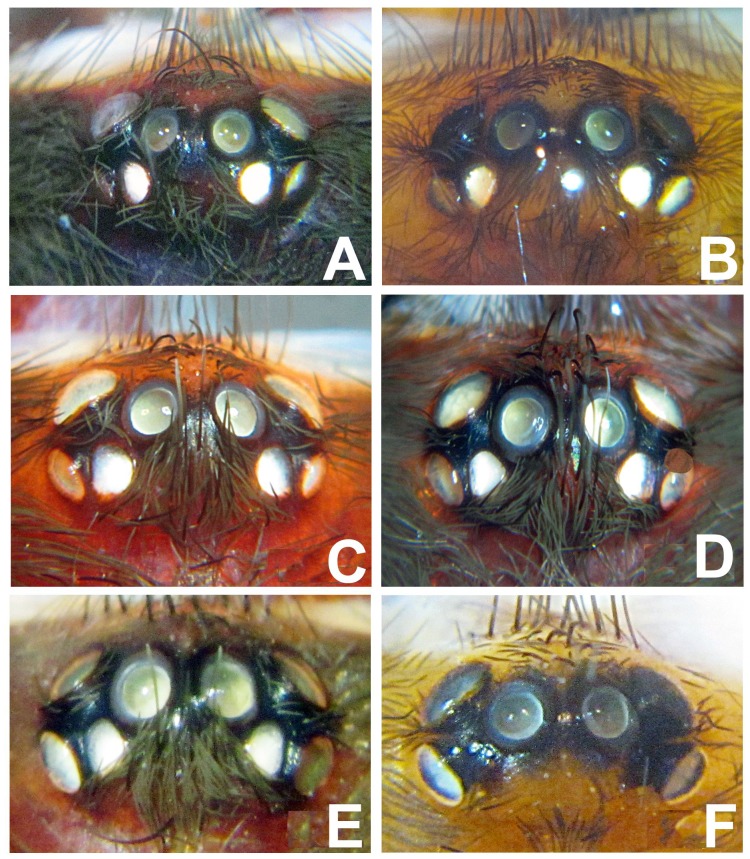
Eyes in *Heterophrictus* and *Neoheterophrictus*. A. *Heterophrictus raveni* **sp. nov.** holotype male; B. *Heterophrictus raveni* **sp. nov.** paratype female; C. *Heterophrictus aareyensis* **sp. nov.** holotype male; D. *Neoheterophrictus amboli* **sp. nov.** holotype male; E. *Neoheterophrictus smithi* **sp. nov.** holotype male; F. *Neoheterophrictus smithi* **sp. nov.** female.
